# A phase 2a double-blind, placebo-controlled, randomized clinical trial evaluating the efficacy and safety of NuGel, a novel topical GPCR19-mediated inflammasome inhibitor, in patients with mild to moderate atopic dermatitis: a proof-of-concept study with *Post-hoc* biomarker analysis

**DOI:** 10.3389/fimmu.2025.1560447

**Published:** 2025-05-19

**Authors:** Gyeong Ho Baek, Bo Ri Kim, Jung-Won Shin, Chang Hun Huh, Jungjoong Hwang, Sungmin Ko, Siwon Kim, Pil-Su Ho, Kyu-Han Kim, Chun Wook Park, Seong Jun Seo, Chang-Ook Park, Dongyoon Shin, Yeongshin Kim, Youngsoo Kim, Seung-Yong Seong, Jung-Im Na

**Affiliations:** ^1^ Department of Dermatology, Seoul National University Bundang Hospital, Seongnam, Republic of Korea; ^2^ Department of Dermatology, Seoul National University College of Medicine, Seoul, Republic of Korea; ^3^ Aimce Center, Shaperon Inc., Gangnam-gu, Seoul, Republic of Korea; ^4^ Department of Dermatology, Veterans Health Service Medical Center, Seoul, Republic of Korea; ^5^ Department of Dermatology, Kangnam Sacred Heart Hospital, Hallym University, Seoul, Republic of Korea; ^6^ Department of Dermatology, Chung-Ang University College of Medicine, Seoul, Republic of Korea; ^7^ Department of Dermatology, Severance Hospital, Yonsei University College of Medicine, Seoul, Republic of Korea; ^8^ Proteomics Research Team, CHA Institute of Future Medicine, Seongnam, Republic of Korea; ^9^ Department of Medical Science, School of Medicine, CHA University, Seongnam, Republic of Korea; ^10^ Wide River Institute of Immunology, Seoul National University College of Medicine, Hongcheon, Gangwon, Republic of Korea; ^11^ Department of Microbiology and Immunology, Seoul National University College of Medicine, Seoul, Republic of Korea; ^12^ Department of Biomedical Sciences, Seoul National University College of Medicine, Seoul, Republic of Korea

**Keywords:** atopic dermatitis, GPCR19, inflammasome, precision medicine, taurodeoxycholic acid, clinical trial

## Abstract

**Background:**

Current guidelines to treat atopic dermatitis (AD) overlook disease heterogeneity, limiting personalized care. This study assessed NuGel, a topical GPCR19 agonist, for efficacy, safety, and predictive baseline biomarkers in AD patients.

**Methods:**

In a multicenter, double-blind, randomized, placebo-controlled Phase 2a trial (August 2020–September 2021, five hospitals, 80 participants), patients received placebo, 0.3% NuGel, or 0.5% NuGel twice daily for four weeks.

**Results:**

NuGel (0.3% [Nu0.3] and 0.5% [Nu0.5]) was well-tolerated, with no adverse drug reactions or serious adverse events. Nu0.3 showed a significant decrease in EASI score from baseline (-12.2%, [-30.3%, 5.9%], p = 0.04). Treatment with Nu0.5 resulted in a numerically decreased EASI score (-11.9%, [-34.9%, 11.1%], p > 0.05), which is comparable with placebo group (-2.9%, [-21.5%, 15.6%], p > 0.05). No significant difference was observed between groups (p>0.05). Plasma proteomic analysis identified biomarkers associated with blood coagulation, complement activation, and cell adhesion as predictors of response to Nu0.5. Patients with baseline profiles characterized by K2C5^high^, ENTP6^low^, or CRK^low^ demonstrated significant clinical improvement when treated with Nu0.5 compared to the placebo group. Among these, the CRK^low^ subgroup, comprising 54.3% of the biomarker analysis set, showed a ΔEASI of -61.3% [-99.9, -22.8; p = 0.003] and a ΔIGA of -35.2% [-58.2, -12.1; p = 0.004] compared to the placebo group. The biomarker signature demonstrated high predictive accuracy (AUC = 0.92, p = 0.002). Logistic regression analysis revealed that the threshold of predicted probability derived from the baseline plasma level of K2C5 and ENTP6 successfully stratified 100% of participants who responded to Nu0.5 (ΔEASI from baseline ≤ -50%), whereas none (0%) in the placebo group responded (p = 0.035).

**Conclusion:**

Baseline biomarkers, such as K2C5, ENTP6, and CRK, may serve as predictors of clinical improvement in AD patients treated with Nu0.5, highlighting the potential for personalized treatment strategies. Further research is required to validate these findings in larger patient cohorts.

**Clinical trial registration:**

https://clinicaltrials.gov/, identifier NCT04530643.

## Introduction

Atopic dermatitis (AD) is a chronic inflammatory skin disease characterized by recurrent eczema and severe pruritus (1), affecting up to 20% of children and 10% of adults in developed countries (1). While >70% of AD patients experience mild symptoms (1, 2), the severe itching associated with the disease significantly impacts quality of life, often associated with higher total quality adjusted life-years (QALYs) loss than other chronic diseases like diabetes or heart disease (3).

Topical treatments are the first-line approach for mild-to-moderate atopic dermatitis (AD), with options such as topical corticosteroids (TCS), calcineurin inhibitors, and moisturizers (4). Systemic treatments, including biologics (e.g., dupilumab), JAK inhibitors (e.g., upadacitinib), and conventional immunosuppressants (e.g., cyclosporine), are reserved for moderate-to-severe AD or cases unresponsive to topicals, offering rapid symptom relief (5). While topical therapies are affordable and suitable for localized flares, systemic options are more effective for widespread or severe disease. Current treatments, including corticosteroids and calcineurin inhibitors, are effective, but their long-term use is limited due to adverse events (6). Newer therapies, such as monoclonal antibodies and Janus kinase (JAK) inhibitors, have shown improved outcomes (5). However, monoclonal antibodies have been associated with anti-drug antibody responses, while JAK inhibitors sometimes come with risks, including infections, cardiovascular events, and malignancies, as highlighted by their black box warnings (7, 8). Although these are currently available treatment options in clinics, there remains a critical need for new therapies that offer improved efficacy and fewer adverse events.

The recognition of heterogeneity in AD patients emerged in the latter half of the 20th century, with both clinicians and biostatisticians acknowledging its importance in treatment evaluation (9). This heterogeneity, as eloquently described by Kravitz (10), emphasized the need for personalized therapy within the framework of biomarker-driven medicine. This idea contributed to the development of precision medicine, a paradigm that optimizes treatment decisions by leveraging patient heterogeneity through biomarker-based approaches, providing the right treatment to the right patient at the right time.

The complexity and heterogeneity of AD pathophysiology, primarily driven by T-helper (Th) 2 cell-mediated inflammation (11), alongside impaired skin barrier function, dysbiosis, genetic predisposition, and environmental factors, have posed challenges in the development of novel therapeutic strategies (12, 13). While various biomarkers, such as nitric oxide synthase 2 (NOS2/iNOS), human beta-defensin-2 (hBD-2), matrix metalloproteinase 8/9 (MMP8/9), and fatty acid-binding protein 5 (FABP5), have been proposed for predicting AD endotypes (14), they have proven insufficient for personalized medicine.

Predictive biomarkers represent significant progress towards translating precision medicine into clinical practice (9). These biomarkers hold the potential to differentiate patients who are likely to benefit from a specific therapy from those who are not (9). Identifying individuals who are likely to experience improved clinical outcomes with treatment, and those who are not, is crucial for optimizing therapeutic strategies.

Keratinocytes and bone marrow-derived immune cells, alongside Th2 cells, all play significant roles in AD pathogenesis (15–17). The inflammasome pathway in these cells is activated by damage-associated molecular patterns (DAMPs) (18) and pathogen-associated molecular patterns (PAMPs) (19), contributing to the initiation and exacerbation of AD (20, 21). For instance, Malassezia and house dust mites trigger NLRP3 inflammasome activation in keratinocytes, leading to the release of inflammatory cytokines such as interleukin (IL)-1β and IL-18, thus exacerbating AD symptoms (22, 23).

Taurodeoxycholic acid (TDCA) inhibits P2X7 receptor (P2X7R)-mediated NLRP3 inflammasome activation in keratinocytes and innate immune cells as a GPCR19 agonist (24), which play a crucial role in AD pathogenesis. By modulating the NF-κB and NLRP3 pathways, TDCA inhibits inflammasome activation (24). The topical formulation of TDCA (NuGel) demonstrated efficacy in alleviating the inflammatory features of AD in multiple mouse models (24). Beyond inhibiting inflammasome activation in AD mouse models, TDCA also increased regulatory T cell (Treg) numbers in AD skin, further highlighting its anti-inflammatory efficacy in regulating Th2-mediated AD pathogenesis (24). Preclinical studies in rats and dogs showed its safety (25, 26). Phase I trials with intravenous or topical TDCA formulations did not report any serious adverse events (NCT04255979, NCT03492398).

This study utilized MRM-MS-based proteomic analysis of baseline blood samples to identify predictive biomarkers that predict patient responsiveness to NuGel treatment over a 4-week period.

### Objectives

The primary objective of this study was to compare the percentage change from baseline in the Eczema Area and Severity Index (EASI) score at the end of the 4-week treatment period between the placebo and NuGel arms. Secondary objectives included evaluating changes in the Investigator’s Global Assessment (IGA) score. Additional objectives were to assess the proportion of participants achieving EASI50 (i.e., ≥50% improvement in EASI score) at week 4, stratified by biomarkers identified from baseline plasma using MRM-MS, as well as the percentage change from baseline in EASI and IGA scores between the placebo and NuGel arms after stratification by multiple variables.

## Materials and methods

### Design and setting

This study involved a multicenter, double-blind, randomized, placebo-controlled phase 2a clinical trial (NCT04530643) to evaluate the efficacy and safety of NuGel (topical TDCA) in adult patients with mild to moderate AD. The trial was conducted in accordance with good clinical practice (GCP) guidelines and relevant regulations. The research protocol, consent forms, and related documents were approved by the Institutional Review Boards (IRBs) of the 5 participating university hospitals in Korea. Informed consent was obtained from all participants prior to enrollment. This report followed the recommendation of the Consolidated Standards of Reporting Trials (CONSORT) checklist (27).

### Participants

Enrollment occurred between 26th August 2020 and 7th September 2021. Eighty eligible participants were randomly assigned (1:1:1 ratio) to receive 0.3% NuGel (Nu0.3), 0.5% NuGel (Nu0.5), or placebo. Randomization was performed using a stratified block randomization method by institution, with block sizes of 3 and 6. An independent randomization coordinator generated the randomization codes using SAS software before the trial commenced. Blinding was maintained throughout the clinical management, data collection, and statistical analysis phases. As this was an exploratory study, the sample size was not determined based on statistical considerations.

Inclusion criteria included adult subjects aged 19 years or older with a clinical diagnosis of AD as defined by the Hanifin and Rajka criteria (28), a baseline Investigator Global Assessment (IGA) score of mild (2) or moderate (3), and a baseline body surface area (BSA) involvement of 5% to 40%. Subjects who received a detailed explanation of this clinical trial, demonstrated understanding, voluntarily agreed to participate, and provided written consent to comply with precautions, including contraception, were included. Exclusion criteria included; 1) Individuals with a history of hypersensitivity or clinically significant allergic reactions to drugs containing Taurodeoxycholate or general medications (e.g., aspirin, antibiotics). 2) Individuals with severe comorbid conditions that may adversely affect clinical trial participation, as determined by the investigator. Examples include clinically significant diseases affecting the liver, kidneys, respiratory system, endocrine system, or nervous system, as well as hematologic malignancies, psychiatric disorders, bleeding disorders (e.g., hemophilia, von Willebrand disease), and cardiovascular diseases (e.g., coronary artery disease, congestive heart failure, arrhythmias, cerebrovascular disease). 3) Individuals with systemic infection symptoms at screening. 4) Individuals with asthma at screening. 5) Individuals with a history of oral steroid, oral antibiotic, systemic photochemotherapy, or immunosuppressant use within one month before the first scheduled dose. 6) Individuals who received approved treatments for atopic dermatitis, such as Dupilumab (brand name: Dupixent^®^), within six months before the first scheduled dose. 7) Individuals with a history of topical steroid or antibiotic use within two weeks before the first scheduled dose. 8) Individuals taking contraindicated medications or those requiring prohibited drugs during the study period. 9) Individuals with renal impairment, as evidenced by creatinine levels exceeding twice the upper limit of normal (ULN) at screening. 10) Individuals with hepatic impairment, as evidenced by AST and ALT levels exceeding twice the ULN at screening. 11) Individuals who participated in another clinical trial or a bioequivalence study and received study drugs within six months before the first scheduled dose (The time since participation is based on the dosing date of the investigational product in the prior study). 12) Individuals with a known history of HIV infection or a positive HIV serological test at screening. 13) Individuals with positive or indeterminate results for hepatitis B surface antigen (HBsAg), hepatitis B core antibody (HBcAb), or hepatitis C antibody at screening. 14) Individuals with coexisting skin conditions that may interfere with clinical trial evaluations, such as acne, impetigo, chickenpox, active herpes simplex at baseline, corticosteroid-induced perioral dermatitis, tinea corporis/intertriginosa, head lice, or scabies. 15) Individuals with a history of malignancy within the past five years before the baseline visit. 16) Individuals who started treatment for atopic dermatitis using prescription moisturizers or moisturizers containing ceramides, hyaluronic acid, urea, or filaggrin additives during the screening period. 17) Individuals with a history of alcohol or substance abuse within two years before the screening visit. 18) Pregnant or breastfeeding individuals, or those planning to conceive or breastfeed during the clinical trial. 19) Any other individuals deemed unsuitable for the clinical trial by the investigator.

Participants could discontinue the clinical trial at any time upon their request or be withdrawn at any time at the discretion of the investigator or sponsor due to safety, behavioral, or administrative reasons. The investigator inquired about the reason for withdrawal, requested the return of all unused investigational products, and asked the participant to complete the final visit. If applicable, the investigator made every effort to follow up on any unresolved adverse events. Situations in which a participant could discontinue the clinical trial included: Withdrawal of consent for trial participation by the participant, violation of inclusion/exclusion criteria, non-compliance with the study protocol as specified in the clinical trial protocol, difficulty in conducting the clinical trial due to adverse events or serious adverse events, as determined by the investigator, non-compliance with the investigator’s or study staff’s instructions by the participant, issues with administering the investigational product to the participant, inability to track or follow up with the participant, any other circumstances where the investigator deems the continuation of the clinical trial inappropriate. The following were considered major protocol violations requiring participant withdrawal from the clinical trial: failure to obtain informed consent, violation of inclusion/exclusion criteria, use of prohibited concomitant medications during the clinical trial period, missing primary efficacy assessment from baseline to the end of the study, compliance with the investigational product below 80%.

### Treatment protocol

From day -21 to day 0, participants underwent screening for eligibility (**Supplementary Figure S1**). On day 1, participants were randomly assigned to one of three treatment arms. Participants were instructed to apply the assigned study gel to the affected lesions twice daily for 28 days. Treatment adherence was monitored, and those with adherence rates below 80% were considered major protocol violations and withdrawn from the study. Concomitant medications were allowed if the investigator determined they were unlikely to affect the study outcome.

### Outcomes

The primary endpoint was the percentage change from baseline in Eczema Area and Severity Index (EASI) score (29) at the end of the treatment period (4 weeks). A negative change from baseline indicated clinical improvement. Secondary endpoints included changes in IGA score. The investigator assessed EASI and IGA scores at baseline, week 2, and week 4. Additional exploratory endpoints included the proportion of participants achieving EASI50 (i.e., ≥50% improvement in EASI score, respectively) at week 4 after stratification by multiple variables.

### Safety assessments

Safety was assessed through treatment emergent adverse events (TEAEs), adverse drug reactions (ADRs), serious adverse events (SAEs), serious adverse drug reactions (SADRs), and discontinuation of study drugs due to adverse events. Vital signs, and laboratory tests including complete blood count, lipid profile, and liver and renal function were monitored. Adverse events were recorded using the Medical Dictionary for Regulatory Activities (MedDRA, version 24.0).

TEAE refers to any harmful and unintended sign (including abnormal laboratory test results), symptom, or disease occurring in a participant who has received the investigational product, regardless of whether there is a causal relationship with the investigational product. Adverse events include, but are not limited to: clinically significant abnormal laboratory findings, clinically significant symptoms or signs, changes in physical examination results, hypersensitivity reactions, and progression or worsening of a pre-existing condition. ADR refers to any harmful and unintended reaction occurring at any dose of the investigational product where a causal relationship with the investigational product cannot be ruled out. A SAE or SADR refers to any AE or ADR occurring at any dose of the investigational product that meets one of the following criteria: results in death or is life-threatening, requires hospitalization or prolongation of existing hospitalization, causes persistent or significant disability or functional impairment, results in congenital anomaly or birth defect, and other medically significant conditions. Even if an event does not fall under the categories listed above, it was considered a SAE if it was deemed to have a significant impact on the participant’s well-being and health. The determination was made based on the medical judgment of the responsible physician and relevant experts, and appropriate actions were taken accordingly.

### Plasma sample preparation for MRM-MS analysis

Plasma samples were collected from participants at baseline (day 1) and processed for mass spectrometry-based proteomic analysis (MRM-MS) to identify biomarkers predicting treatment response. Plasma was centrifuged at 10,000 × g for 10 minutes at 4°C, and the supernatant was filtered through a 0.22 µm membrane at room temperature at 12,000 × g. Protein concentrations were measured using the Pierce™ BCA Protein Assay Kit (Thermo Scientific). Samples were denatured and reduced, alkylated, and digested with trypsin according to standard protocols. Digestion was quenched with formic acid, and the supernatant was collected for further analysis.

### Synthetic internal standards and quality control for MRM-MS

Stable isotope-labeled synthetic internal standard peptides (SIS peptides) were added to the plasma samples to quantify the biomarkers. SIS peptides synthesized with neutral isotopes were mixed with the plasma samples to correct for potential variations in peptide ionization and other error sources. SIS concentrations were optimized to ensure accurate quantification and proper integration of peak areas for the target proteins. The consistency of peptide quantification was verified, and peptides with poor signal-to-noise ratios were removed.

### MRM-MS analysis

MRM-MS analysis was performed using an Agilent 6495 triple quadrupole (QqQ) mass spectrometer (Agilent Technologies, Santa Clara, CA) coupled with an Agilent 1260 Infinity HPLC system (Agilent Technologies). The HPLC solvents consisted of 0.1% formic acid (FA) in water (solvent A) and 0.1% FA in acetonitrile (solvent B). Forty microliters of the pre-treated plasma samples were injected onto a guard column (2.1 × 30 mm, 1.8 µm, 80 Å, Agilent Technologies). Samples were desalted for 10 minutes at a flow rate of 5 µL/min using 3% solvent B. The samples were then transferred to the analytical column (0.5 × 150 mm, 3.5 µm, 80 Å, Agilent Technologies), where they were eluted at a flow rate of 40 µL/min with 3% solvent B for 5 min. The column was maintained at 40°C in the column oven. Peptides (10 µg) were separated on the analytical column and eluted using a linear gradient (3% to 40%) of acetonitrile containing 0.1% FA for 125 min at a flow rate of 40 µL/min. Mass spectra were acquired in positive ion mode with the following parameters: ion spray capillary voltage 2500 V, nozzle voltage 2000 V, cell acceleration voltage 5 V, delta EMV 200 V, and fragmentor voltage 380 V. Drying gas flow was set to 15 L/min at 250°C, and the sheath gas flow was set to 12 L/min at 350°C. Collision energy (CE) was optimized by summing individual transition intensities to maximize peak areas. The base CE was calculated as follows:


$$\begin{array}{c} \text{For doubly charged precursor ions}:\;CE\\ =0.031\;x\;\left(\frac{m}{z}of\;precursor\right)+1 \end{array}$$

$$\begin{array}{c} \text{For triply charged precursor ions}:\;CE\\ =0.036\;x\;\left(\frac{m}{z}of\;precursor\right)-4.8 \end{array}$$

Block randomization was used to ensure a robust study design.

### Filtering target peptides

As shown in **Supplementary Figure S2A**, targets were selected based on consistent quantification across all samples using filtering criteria. Firstly, the intensity of the SIS or endogenous peaks was evaluated. Samples with SIS peak intensities below 100 counts, SIS peaks that failed to elute with the corresponding endogenous peptides, or elution peaks exhibiting an asymmetric profile were excluded. The remaining peaks were integrated using Skyline^®^ to calculate the peak area ratio (PAR) between the endogenous and SIS peptides. The amount of each target peptide was determined using the formula:


Endogeneous target protein (fmol)=PAR x SIS injected

### A statistical modeling to identify predictive biomarkers

To identify biomarkers predictive of patient response, a model was developed using biomarker levels as predictive variables to estimate clinical outcomes, specifically ΔEASI (Nu0.5 - Placebo) and ΔIGA (Nu0.5 - Placebo). ROC analysis was conducted to determine the optimal cut-off point for baseline plasma levels of each biomarker for predicting clinical outcomes. The cut-off point represented the value where the biomarker’s discriminatory ability was maximized. In addition, this approach aimed to balance sensitivity and specificity, maximizing the biomarker’s ability to distinguish between responders and non-responders.

The cut-off point that maximizes Youden Index (J), known as the optimal cut-off point, optimizes the biomarker’s discriminatory ability by balancing sensitivity and specificity (30, 31). The J was calculated as described:


J=sensitivity+specificty−1

Differentially expressed proteins (DEPs) between the responders and non-responders were first identified based on the change in EASI50 from baseline to week 4 after Nu0.5 treatment. EASI50 responders were defined as those with ΔEASI ≤ -50% from baseline, indicating a ≥50% improvement in EASI score compared to baseline, while non-responders had ΔEASI > -50% from baseline. Forty-two DEPs between responders and non-responders with an AUROC exceeding 0.7 after Nu0.5 treatment were selected for further analysis (**Supplementary Figure S2**, **Supplementary Table 1**). For certain biomarkers, favorable clinical outcomes were observed when plasma levels were below the cut-off point, while for others, favorable outcomes were associated with plasma levels exceeding the cut-off point.

These optimized cut-off points were then utilized to analyze clinical outcomes and evaluate the positive predictive value (PPV) and negative predictive value (NPV) of individual biomarkers, calculated as follows:


PPV=True positives/(True positives+False positives)

NPV=True negatives/(True negatives+False positive)

Univariate analysis was employed to compare ΔEASI between participants categorized into biomarker^lo^ and biomarker^hi^ groups. The treatment benefit (B) of Nu0.5 was evaluated after stratification using each biomarker, defined as:


B=I [Y(0)≤Y(1)]

where Y(0) and Y(1) represent the potential outcomes under placebo and Nu0.5 treatment, respectively. The indicator function I[Y(0) ≤ Y(1)] was considered true if the p-value was below 0.05 for both ΔEASI (Nu0.5 - Placebo) and ΔIGA (Nu0.5 - Placebo).

Five biomarkers were selected showing treatment benefit (B = true) of Nu0.5. Benefit models for the five biomarkers were developed using logistic regression. Baseline K2C5 levels were log-transformed before inclusion as covariates, while baseline levels of other biomarkers were included without transformation. These models incorporated baseline biomarker levels as predictive variables to evaluate their relationship with ΔEASI (Nu0.5 - Placebo) and ΔIGA (Nu0.5 - Placebo). Furthermore, 2 or 3 combinations involving 42 biomarkers were evaluated. The cut-off points of predicted probability (pp) of each combination were determined by logistic regression analysis. Briefly, ROC curves using the pp from the logistic regression model were generated and AUCs were calculated to assess overall discriminatory power. Youden’s index for various cut-off points of pp was calculated on the ROC curve to identify the optimal cut-off point of pp that maximizes Youden’s index.

### Heatmap cluster analysis

Baseline biomarker expression data from 20 patients treated with 0.5% NuGel was utilized to identify patterns associated with EASI50 response through hierarchical clustering. Prior to clustering, all biomarker levels were standardized using z-score normalization with scikit-learn (StandardScaler, Python) to ensure comparability between biomarkers irrespective of their original scales (32). Hierarchical clustering was performed using the Canberra distance metric and complete linkage (32), chosen for its ability to handle diverse feature scales and form well-defined clusters. Clustering and heatmap visualization were generated using the clustermap function (seaborn, Python), with subsequent formatting and customization implemented through matplotlib (33). Standardized data was represented using a diverging color map to highlight relative differences in biomarker levels between EASI50 responders and non-responders. This approach enabled unbiased identification of potential relationships between biomarker clusters and patient response groups.

### Principal component analysis

PCA was performed on the 42 baseline biomarker profiles of 20 subjects treated with Nu0.5 for dimensionality reduction and identification of principal axes of variation. Before analysis, all biomarker values were standardized (mean = 0, standard deviation = 1) using the StandardScaler function (scikit-learn, Python) to ensure equal contribution of each biomarker regardless of scale (32). Participants were categorized as responders or non-responders based on their EASI50 response. Data visualization was generated using matplotlib (33).

### Volcano plot analysis

Baseline biomarker levels were compared between clinical responders and non-responders categorized based on EASI50 response. Group-wise comparisons were performed using Student’s *t*-test to determine the p-value for each biomarker. The fold change in biomarker levels was quantified as the log2 transformation of the mean biomarker level in responders divided by the mean biomarker level in non-responders, representing the relative expression difference between groups. The results of the differential analysis were visualized using volcano plots generated in GraphPad Prism (version 10.1.1, GraphPad Software Inc., CA).

### Pathway and gene set enrichment analysis

Forty-two biomarkers were selected and ranked based on differential expression (log2 fold change) between EASI50 responders vs. non-responders. The KEGG pathway database was utilized to define pathways related to inflammation, immune responses, and skin barrier dysfunction, crucial aspects of AD pathogenesis. Plasma biomarkers were mapped to gene sets using UniProt IDs. Gene set enrichment analysis (GSEA) was performed using the ranked list of plasma biomarkers and predefined gene sets (34). The analysis employed the GSEA algorithm to assess whether members of a gene set were disproportionately represented at the top or bottom of the ranked list. Enrichment scores (ES) were calculated for each gene set to quantify the extent of overrepresentation at the extremes of the ranked list. A permutation test (1,000 permutations) was applied to calculate p-values. False discovery rate (FDR) was used to control for multiple comparisons. ES was normalized to account for variations in gene set size. Gene sets with normalized ES (NES) > 1.3 and FDR adjusted p-value < 0.05 were considered significantly enriched. The biomarkers contributing most to enrichment were identified through leading-edge analysis. Enriched pathways were interpreted within the context of AD pathogenesis, focusing on innate and adaptive immune responses.

### Statistical analysis

We used the CONSORT reporting guidelines (27). Safety Analysis Set (SAS) consists of all subjects who were randomized and received at least one dose of the investigational product. Safety analysis was based on adverse events and laboratory results within the SAS (n = 80). Full analysis set (FAS) consists of subjects who received at least one dose of the investigational product and had at least one efficacy assessment measured within four weeks after the first dose, starting from baseline. Efficacy analysis was conducted in the FAS (n = 79) otherwise denoted. Per-protocol set (PPS, n = 63) consists of subjects included in the FAS who completed the clinical trial as per the study protocol. Subjects excluded from the PPS are classified based on the dropout criteria including; EASI score not measured at the 4-week time point (± 3 days), failure to obtain informed consent, violation of inclusion/exclusion criteria, administration of prohibited concomitant medications, non-compliance with drug adherence (less than 80%), randomization error, and administration error of the investigational product. The biomarker analysis set (BAS) included participants from the FAS who had available baseline biomarker data and provided written consent for the use of plasma samples in biomarker analysis (n = 70).

Statistical comparisons between treatment groups were performed using analysis of variance (ANOVA). For pairwise comparisons, Student’s *t*-test was used when data sets that met normality assumptions based on the Shapiro–Wilk test; otherwise, the Wilcoxon signed-rank test was applied. Predictive biomarkers were evaluated using ROC curve analysis, with the optimal cut-off point determined by maximizing the Youden index (J). Odds ratios (OR) and 95% confidence intervals (CI) were derived from 2×2 contingency tables comparing subgroups defined by biomarker levels (low vs. high) to clinical outcomes (responders vs. non-responders), with p-values calculated using Fisher’s exact test. The Haldane-Anscombe correction was applied when zero counts were involved (35). All statistical analyses were performed using SAS software (version 9.4) and GraphPad Prism (version 10.1.1). The area under the curve (AUC), standard error, and p-values were determined using the ROC analysis module in GraphPad Prism version 10.1.1.

## Results

### Study population:

**Figure 1:**
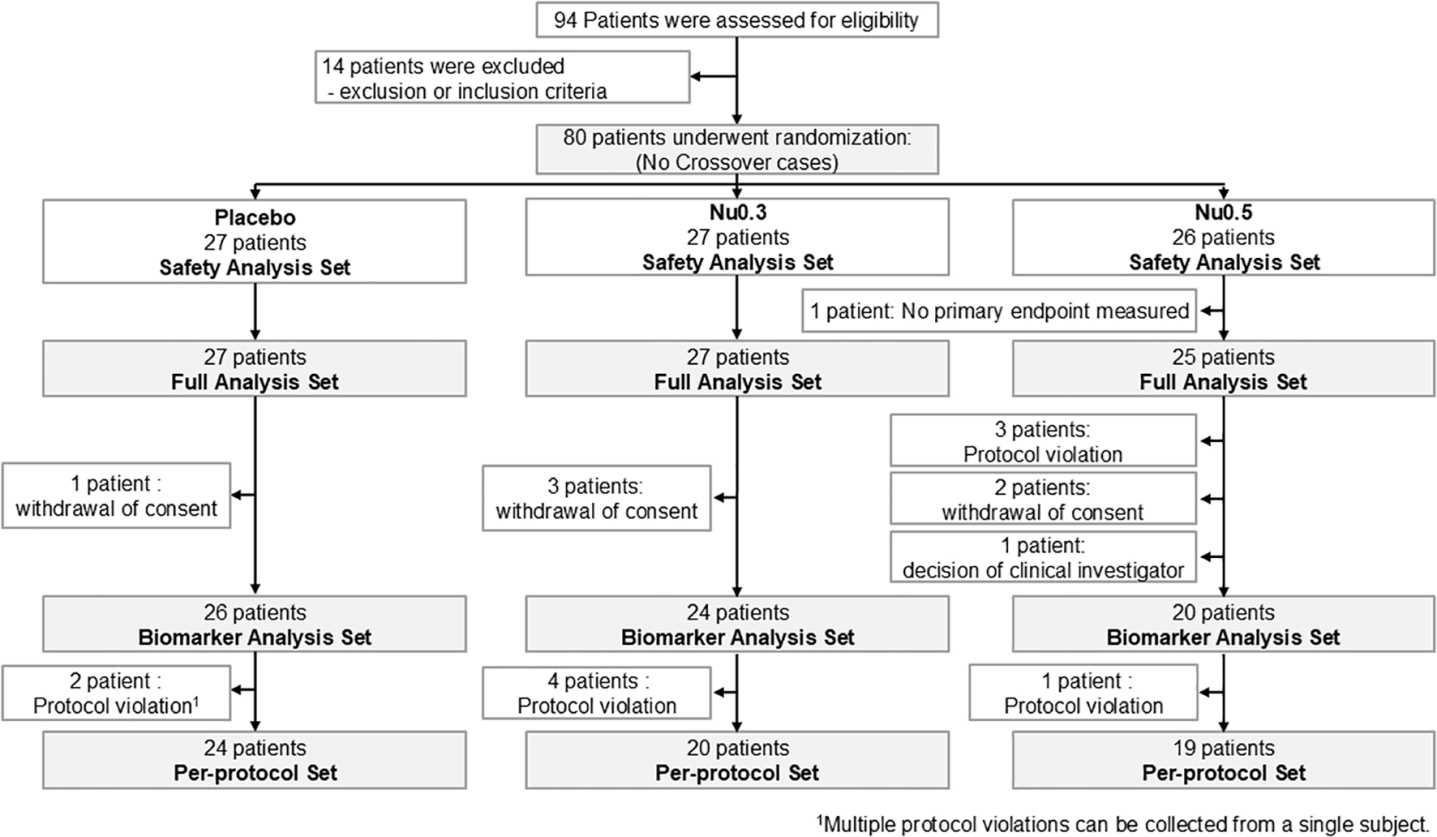
CONSORT flow diagram of patient enrollment, randomization, and follow-up throughout the study. The trial began with a screening phase to assess participant eligibility based on predefined criteria. Eligible participants were then randomized into three treatment arms (Nu0.5, Nu0.3, or placebo) to ensure balanced group distribution.

As shown in the CONSORT flow diagram (**Figure 1**), 94 patients were screened from August 13, 2020, to September 7, 2021, of whom 80 eligible patients (SAS) were randomly assigned to placebo (n = 27), Nu0.3 (n = 27), or Nu0.5 (n = 26) at five hospitals. A total of 79 patients (98.75%) were included in the FAS for efficacy evaluation. One patient in the Nu0.5 group was excluded from FAS because the primary efficacy endpoint was not assessed within 4 weeks (**Supplementary Table 2**). Seventy patients (87.5% of SAS) completed the study and were included in the BAS (**Supplementary Table 2**). Seventeen patients were excluded from the SAS group, resulting in a PPS of 63 patients (78.75%) (**Supplementary Table 2**). There were no statistically significant differences in demographic characteristics, disease characteristics, or anthropometric data between treatment groups (**Table 1**). In summary, the median age was 26 (19–65) years, and 62.0% of the participants were under 30 years old. The cohort consisted of 38 males (48.1%) and 41 females (51.9%), reflecting a balanced gender distribution. The mean baseline EASI score was 8.27 ± 4.36, and IGA scores were 2 (59.5%) and 3 (40.5%). The mean duration of atopic dermatitis was 18.4 ± 10.4 years, with no significant differences between treatment groups. Baseline comorbidities were not significantly different across the study population (**Supplementary Table 3**). Treatment adherence was generally good in all groups (**Supplementary Table 4**). There were no significant differences in the mean adherence rate (p=0.89) or the proportion of subjects with adherence exceeding 80% (p = 0.42) between treatment groups.

**Table 1:** Baseline demographic factors of FAS.

	Placebo (N = 27)	Nu0.3 (N = 27)	Nu0.5 (N = 25)	Total (N = 79)	*p* value
Age (years), *n* (%)					0.4409
Mean ± SD	28.48 ± 8.79	30.19 ± 8.81	27.96 ± 9.88	28.90 ± 9.09	
Median (min, max)	26.00 (19.00, 45.00)	28.00 (19.00, 46.00)	26.00 (19.00, 65.00)	26.00 (19.00, 65.00)	
Sex, *n* (%)					0.3498
Male	14 (51.85)	10 (37.04)	14 (56.00)	38 (48.10)	
Female	13 (48.15)	17 (62.96)	11 (44.00)	41 (51.90)	
^1^BMI (kg/m^2^)					0.5713
Mean ± SD	23.15 ± 3.26	22.63 ± 3.46	23.60 ± 3.07	23.11 ± 3.25	
Median (min, max)	23.60 (16.90, 29.70)	22.30 (17.10, 31.40)	23.50 (16.60, 30.10)	23.20 (16.60, 31.40)	
Baseline EASI					0.5024
Mean ± SD	8.50 ± 4.80	7.41 ± 3.77	8.96 ± 4.47	8.27 ± 4.36	
Median (min, max)	7.00 (1.10,18.30)	7.40 (2.20,18.80)	7.80 (3.20,22.50)	7.30 (1.10, 22.50)	
Baseline IGA, *n* (%)					0.1422
2	17 (62.96)	19 (70.37)	11 (44.00)	47 (59.49)	
3	10 (37.04)	8 (29.63)	14 (56.00)	32 (40.51)	
Baseline NRS					0.4212
Mean ± SD	5.15 ± 2.14	4.44 ± 2.12	4.80 ± 1.96	4.80 ± 2.07	
Median (min, max)	6.00 (1.00, 9.00)	4.00 (1.00, 9.00)	6.00 (1.00, 8.00)	5.00 (1.00, 9.00)	
Season at enrollment, *n* (%)					0.5577
Spring/summer	20 (74.07)	18 (66.67)	15 (60.00)	53 (67.09)	
Fall/winter	7 (25.93)	9 (33.33)	10 (40.00)	26 (32.91)	
Duration of disease, year					0.1092
Mean ± SD	15.64 ± 10.20	21.53 ± 10.68	17.90 ± 9.73	18.37 ± 10.39	
Median (min, max)	15.58 (0.00, 41.42)	23.36 (1.52, 41.46)	18.48 (0.00, 40.39)	18.48 (0.00, 41.46)	

^1^Body mass index.

### Efficacy in FAS and subgroups:

**Figure 2:**
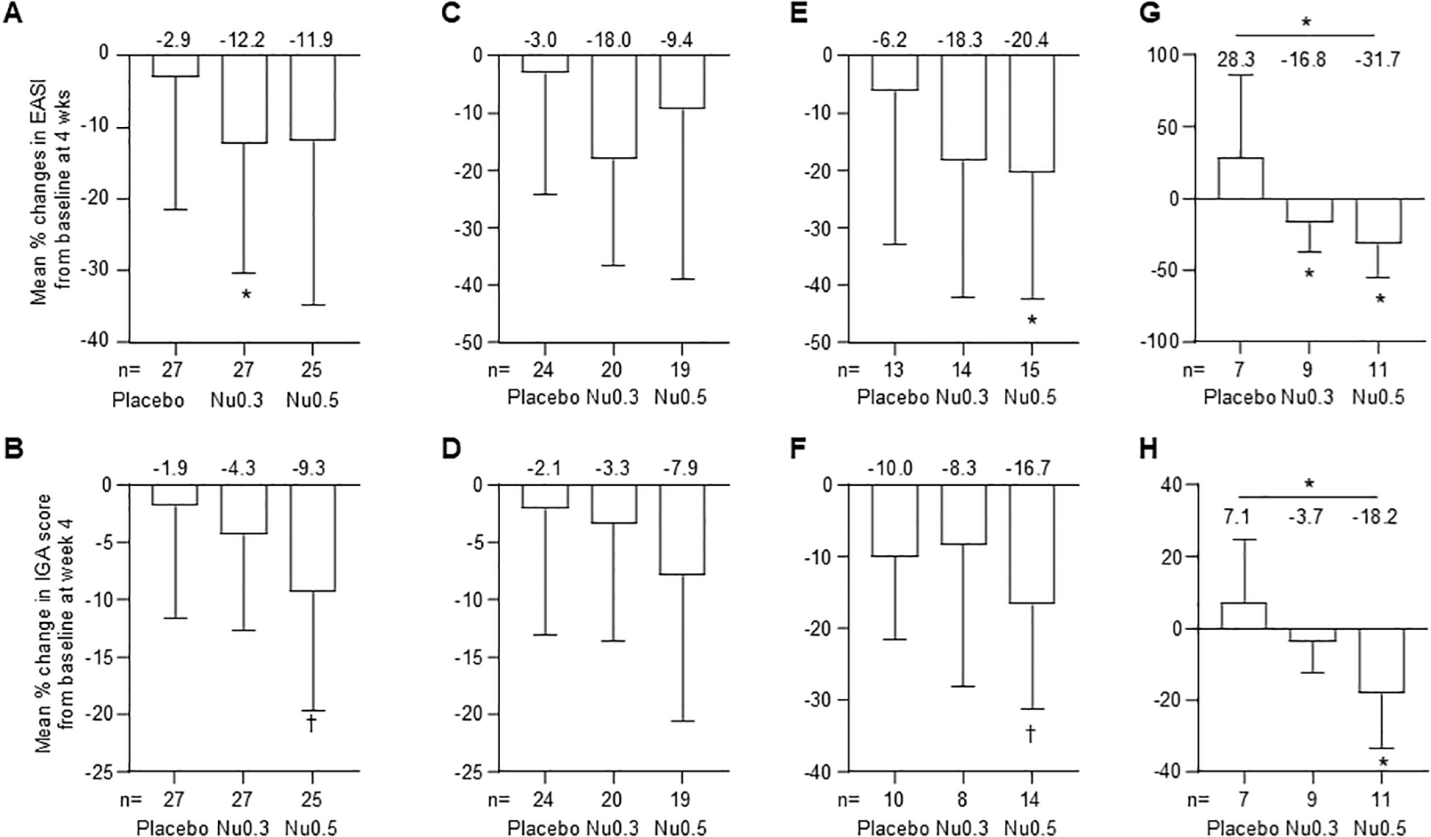
Percent change in EASI and IGA scores at week 4 compared to baseline in various patient groups. **(A, B)** FAS analysis of EASI **(A)** and IGA **(B)** scores. **(C, D)** PPS analysis of EASI **(C)** and IGA **(D)** scores. **(E, F)** Analysis in patients with moderate to severe disease (E, 7 < EASI ≤ 21; F, IGA = 3). **(G, H)** Analysis in patients enrolled from September 2020 to February 2021 for EASI **(G)** and IGA **(H)** scores. Bars represent mean ± 95% CI. (mean values at the top of bars), n = number of patients. ^*^p < 0.05 (Student’s *t*-test); ^†^0.05 < p < 0.09 (Wilcoxon signed-rank test). EASI, Eczema Area and Severity Index; IGA, Investigator’s Global Assessment.

In FAS, the Nu0.3 group showed a significant decrease in EASI score from baseline (-12.2%, 95% CI: -30.3% to 5.9%, p = 0.04) (**Figure 2A**). Treatment with Nu0.5 resulted in a numerically decreased EASI score (-11.9%, 95% CI: -34.9% to 11.1%, p > 0.05), which is comparable with placebo group (-2.9%, 95% CI: -21.5% to 15.6%, p > 0.05). Additionally, 11.1%, 14.8%, and 24.0% of participants in the placebo, Nu0.3, and Nu0.5 groups, respectively, achieved EASI50 response. The Nu0.5 group showed a numerical decrease in IGA score from baseline (-9.3%, 95% CI: -19.7% to 1.0%, p = 0.09), which is comparable with placebo group (-1.9%, 95% CI: -11.6% to 7.9%) (**Figure 2B**). However, the differences in EASI and IGA scores between groups were not statistically significant.

In PPS, the mean percentage change in EASI score from baseline was -3.0% (95% CI: -24.1% to 18.0%, p > 0.05) for the placebo group, -18.0% (95% CI: -36.6% to 0.5%, p = 0.05) for the Nu0.3 group, and -9.4% (95% CI: -39.0% to 20.1%, p > 0.05) for the Nu0.5 group (**Figure 2C**). The mean percentage change in IGA score from baseline was -2.1% (95% CI: -13.1% to 9.0%) for the placebo group, -3.3% (95% CI: -13.6% to 7.0%) for the Nu0.3 group, and -7.9% (95% CI: -20.6% to 4.8%) for the Nu0.5 group (p > 0.05 between groups, **Figure 2D**).

In the FAS subgroup of patients with moderate atopic dermatitis (baseline 7 < EASI ≤ 21), the Nu0.5 group showed a significant decrease in EASI score from baseline (-20.4%, 95% CI: -42.5% to 1.7%, p = 0.04) (**Figure 2E**). Similarly, in the subgroup of patients with moderate atopic dermatitis defined as baseline IGA = 3, the Nu0.5 group showed a borderline significant decrease in IGA score from baseline (-16.7%, 95% CI: -31.3% to -2.0%, p = 0.06) (**Figure 2F**).

Patients recruited from September 2020 to February 2021 (placebo n = 7, Nu0.3 n = 9, Nu0.5 n = 11) showed significant differences in EASI compared to placebo (p = 0.02, **Figure 2G**); At week 4, ΔEASI (Nu0.3 vs. placebo) was –45.1% (95% CI: –94.5% to –4.4%) and ΔEASI (Nu0.5 vs. placebo) was –60.1% (95% CI: –108.7% to –11.4%). Significant differences were also observed in IGA scores compared to placebo (p = 0.03, **Figure 2H**). At week 4, ΔIGA (Nu0.3 vs. placebo) was –10.8% (95% CI: –27.0% to 5.3%) and ΔIGA (Nu0.5 vs. placebo) was –25.3% (95% CI: –47.4% to –3.3%).

These clinical improvements were noted at 2 weeks after treatment (**Supplementary Figure S3**). While not reaching statistical significance, the Nu0.5 group displayed a tendency towards better EASI and IGA scores by the second week across various analysis populations.

### Filtering biomarkers predicting response to Nu0.5 treatment:

An MRM-MS analysis was conducted to evaluate 802 inflammation-related biomarkers in pooled plasma samples (**Supplementary Figure S2A**). After validating assay quality, 502 target proteins were selected based on the following exclusion criteria: (1) peak intensities (SIS or endogenous) below 100 counts, (2) failure of SIS peaks to co-elute with corresponding endogenous peptides, and (3) skewed elution peak profiles. These 502 pooled SIS peptides were combined with pooled plasma from 70 participants at concentrations of 50, 100, 200, and 400 fmol to ensure accurate quantification. Following filtering SIS peptide by linearity (50–400 fmol, with 0.1 < PAR < 1), 469 protein targets were quantified across 70 individual plasma samples. To ensure robust study design, the 70 plasma samples were block randomized and mixed with the optimized 469 SIS peptide pool. The analysis revealed a seven-step magnitude range between the lowest and highest plasma protein levels (**Supplementary Figure S2B**), with approximately 90% of targets exhibiting a five-step dynamic range. The Agilent 6495 QqQ mass spectrometer demonstrated sufficient sensitivity, detecting up to a six-step magnitude range. Principal component analysis (PCA) and evaluation of inter-hospital variation in biomarker levels validated the technical consistency of the measurements, confirming no significant differences across the five participating hospitals (**Supplementary Figures S4A, B**). Biomarkers with an area under the ROC curve (AUROC) below 0.7 were excluded, resulting in the identification of 42 DEPs that distinguished responders from non-responders based on EASI50 following Nu0.5 treatment. These DEPs exhibited high sensitivity and specificity (**Supplementary Table 1**). The 42 DEPs were categorized into two groups: the “hi” group, comprising patients with plasma levels above the cut-off point who responded favorably to Nu0.5, and the “lo” group, including patients with plasma levels below the cut-off point who also responded favorably (**Supplementary Table 1**).

Finally, five biomarkers (K2C5, ENTP6, CRK, IGHA2, and SMOC1) meeting statistical significance (p < 0.05) for ΔEASI (Nu0.5 vs. placebo), ΔIGA (Nu0.5 vs. placebo), and AUROC (> 0.7, responders vs. non-responders) were identified after stratifying patients based on baseline biomarker levels (**Table 2**).

**Table 2:** Clinical improvement of patients after stratification with baseline plasma level of biomarker.

Patient groups stratified with	ΔEASI^1^	*p* value^2^	ΔIGA^1^	*p* value^2^	% BAS^3^	ROC		PPV^4^	NPV^5^
	[95% CI]		[95% CI]			AUC	*p* value		
K2C5^hi^	-94.1 [-146.1 – -42.0]	0.003	-50.0 [-78.4 – -21.6]	0.004	20.0	0.953	0.006	1.000	0.938
ENTP6^lo^	-57.9 [-103.6 – -12.3]	0.017	-42.1 [-67.4 – -16.8]	0.004	37.1	0.893	0.010	0.800	0.933
CRK^lo^	-61.3 [-99.9 – -22.8]	0.003	-35.2 [-58.2 – -12.1]	0.004	54.3	0.923	0.002	1.000	0.846
IGHA2^hi^	-41.7 [-77.2 – -6.1]	0.024	-22.9 [-44.7 – -1.0]	0.041	52.9	0.950	0.001	0.909	1.000
SMOC1^hi^	-53.4 [-86.7 – -20.1]	0.003	-30.6 [-55.1 – -6.0]	0.017	45.7	0.859	0.008	0.875	0.750

^1^Mean (Nu0.5 – Placebo), CI, confidence interval.

^2^Student’s *t*-test (Nu0.5 vs. Placebo).

^3^% of patients in the biomarker analysis set.

^4^Positive prediction value.

^5^Negative prediction value.

### Cluster analysis of clinical response and biomarker levels:

**Figure 3:**
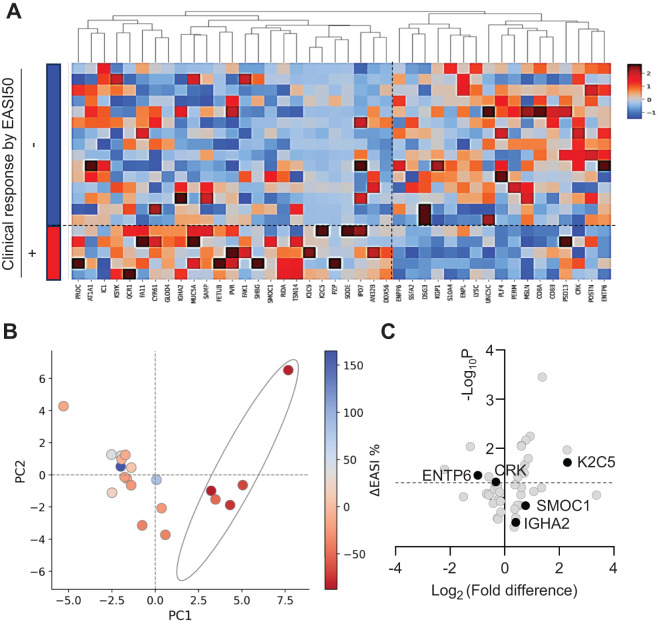
The differential expression of plasma proteins associated with treatment response. **(A)** Heat map of DEPs illustrates the hierarchical clustering of 42 DEPs identified between EASI50 responders and non-responders. Each column represents a different protein, while each row corresponds to an individual patient. The color scale reflects the expression levels, with red indicating higher expression and blue indicating lower expression. **(B)** Principal component analysis plot is based on the expression levels of the 42 DEPs and ΔEASI from baseline, demonstrating the variance in protein expression profiles between EASI50 responders and non-responders. Each point represents an individual patient, with clusters indicating similarities in changes in EASI scores (ΔEASI) and biomarker signatures. Dots within the ellipse represent participants with a ΔEASI from baseline of less than -50%, highlighting a subset of patients who exhibited substantial clinical improvement. **(C)** The volcano plot displays the statistical significance (-Log10 p-value) against fold change (Log2 fold change) for the 42 DEPs. The dashed line indicates the p < 0.05 threshold for significance. Five biomarkers are denoted with filled circles, representing those that showed significant clinical prediction power.

Clinical responses to Nu0.5, evaluated using EASI50, revealed significant clustering of baseline plasma levels among 42 DEPs (**Figure 3A**). The heatmap highlights biomarkers associated with favorable or unfavorable clinical responses, showcasing distinct expression profiles between responders and non-responders categorized by EASI50 response. The hierarchical clustering dendrogram above the heatmap groups biomarkers based on expression similarity, revealing distinct clusters associated with clinical response outcomes. Biomarkers consistently exhibiting high expression (red) or low expression (blue) in specific response groups are likely critical in distinguishing clinical responders from non-responders. These findings propose potential biomarker signatures that could predict clinical responses to Nu0.5 treatment.

### PCA and volcano analysis:

PCA was conducted on the baseline biomarker levels of 20 plasma samples from participants treated with Nu0.5 to reduce dimensionality and assess the ability of biomarkers to distinguish clinical response groups. Patients classified as responders, based on the EASI50 response, were distinctly clustered within the PCA plot (data points that fall within the area defined by the ellipse). Samples with lower -ΔEASI% values from baseline tended to segregate from those with higher -ΔEASI% values along the principal components, suggesting that these components reflect significant variations in biomarker levels correlated with clinical responses. This separation indicates that plasma biomarker levels can effectively stratify patients based on percentage improvement in EASI scores after treatment with Nu0.5, underscoring their potential utility in predicting treatment outcomes.

The volcano plot illustrates the results of a differential analysis comparing baseline plasma levels of 42 biomarkers between clinical responders and non-responders based on the EASI50 response to Nu0.5 treatment (**Figure 3C**). Several biomarkers showed significant fold changes, either upregulated or downregulated, displaying notable p-values. Among them, K2C5 was significantly upregulated (fold change = 4.77, p = 0.018), while ENTP6 was downregulated (fold change = 0.49, p = 0.033). These biomarkers could be considered potential candidates for predicting clinical response in the context of EASI50. The volcano plot effectively highlights biomarkers that exhibit significant changes between groups, providing insights into which biomarkers may act as potential predictors of clinical response.

### Functional Annotation and Gene Ontology of DEPs:

Understanding the biological pathways that differentiate responders from non-responders can inform precision medicine strategies. Functional annotation of DEPs revealed a significant overrepresentation of pathways involved in inflammation, including the complement pathway [fold enrichment (FE) = 39.3, false discovery rate (FDR) = 0.042] and blood coagulation [FE = 27.5, FDR = 0.042] (**Table 3**). These findings align with the inflammatory mechanisms underlying AD and its treatment response. Gene ontology analysis further highlighted heparin-binding proteins (HBPs) (FE = 25.0, FDR = 0.001), and protease inhibitors (FE = 14.7, FDR = 0.032) as enriched molecular functions. HBPs play critical roles in inflammatory responses by acting as chemoattractant, activating monocytes and macrophages, inducing vascular leakage and edema formation (36). Protease inhibitors, closely linked to coagulation, may also influence inflammatory processes (37, 38). Notably, some protease inhibitors regulate the complement system, a key component of the innate immune response (38). These insights suggest that targeting these pathways could enhance therapeutic strategies for atopic dermatitis.

**Table 3:** Functional annotation of 42 biomarkers by ^1^DAVID.

Functional clustering	*p* Value	^2^FE	^3^Ben	^4^FDR	Contributing genes				
Complement pathway	0.002	39.3	0.047	0.042	C8A	C8B	SERPING1		
Blood coagulation	0.005	27.5	0.047	0.042	F11	PROC	SERPING1		
Cell adhesion	0.018	4.7	0.131	0.117	PVR	CCN1	DSG3	MSLN	POSTN
Innate immunity	0.058	4.3	0.242	0.217	C8A	C8B	SERPING1	SYK	
Gene ontology clustering	*p* Value	FE	Ben	FDR	Contributing genes				
Heparin-binding	0.000	25.0	0.001	0.001	PZP		IGHA2	SERPING1	
Protease inhibitor	0.002	14.7	0.032	0.032	PZP		FETUB	SERPING1	
Serine protease inhibitor	0.014	16.0	0.137	0.137	PZP		IGHA2	SERPING1	

^1^Database for annotation, visualization, and integrated discovery.

^2^fold enrichment.

^3^significance by Benjamini-Hochberg procedure.

^4^false discovery rate.

### Gene set enrichment analysis of DEPs:

GSEA was performed to explore the biological pathways associated with the 42 DEPs identified between EASI50 responders and non-responders upon treatment with Nu0.5. The GSEA results highlighted pathways significantly enriched in EASI50 responders; positive correlation with genes up-regulated in comparison of peripheral blood mononuclear cells (PBMC) from patients with type 1 diabetes at the time of the diagnosis versus those at 4 months later [normalized enrichment score (NES) = 1.64, p = 0.000] and negative correlation with genes up-regulated in the *in vitro* follicular dendritic cells from peripheral lymph nodes: non-stimulated versus tretinoin and Pam2CSK4 group (NES = -1.42, p = 0.039) (**Table 4**).

**Table 4:** Gene set enrichment analysis.

^1^Gene sets enriched	^2^NES	^3^ *p*-val	^4^FDR q-val		Leading edge genes		
Positive correlation							
9006_TYPE_1_DIABETES_AT_DX_VS_4MONTH_POST_DX_PBMC_UP	1.64	0.000	0.027		TSPAN14	UQCRC1	PSMD13
11057_EFF_MEM_VS_CENT_MEM_CD4_TCELL_DN	1.36	0.028	0.218		PTK2	TSPAN14	ATP1A1
22601_IMMATURE_CD4_SINGLE_POSITIVE_VS_DOUBLE_POSITIVE_THYMOCYTE_UP	1.39	0.037	0.247		FETUB	DSG3	ENPP6
3982_BCELL_VS_TH1_UP	1.33	0.046	0.259		SYK	MUC5AC	FETUB
13411_NAIVE_VS_IGM_MEMORY_BCELL_DN	1.43	0.026	0.287		TSPAN14	SYK	UQCRC1
40274_CTRL_VS_LEF1_TRANSDUCED_ACTIVATED_CD4_TCELL_DN	1.40	0.027	0.306		IPO7	ATP1A1	UQCRC1
Negative correlation							
19401_UNSTIM_VS_RETINOIC_ACID_AND_PAM2CSK4_STIM_FOLLICULAR_DC_UP	-1.42	0.039	0.254		RIDA	ENTPD6	HSP90B1
37301_LYMPHOID_PRIMED_MPP_VS_RAG2_KO_NK_CELL_DN	-1.49	0.027	0.274		MPO	C8A	KRT5
43260_BTLA_POS_VS_NEG_INTRATUMORAL_CD8_TCELL_UP	-1.32	0.024	0.339		LYZ	ENTPD6	HSP90B1

^1^90006, Genes up-regulated in comparison of peripheral blood mononuclear cells (PBMC) from patients with type 1 diabetes at the time of the diagnosis versus those at 4 months later.

11057, Genes down-regulated in comparison of effector memory T cells versus central memory T cells from peripheral blood mononuclear cells.

22601, Genes up-regulated in immature CD4 single positive cells versus double positive thymocytes.

3982, Genes up-regulated in comparison of B cells versus Th1 cells.

13411, Genes down-regulated in comparison of naive B cells versus IgM-memory B cells.

40274,Genes down-regulated in CD4 T conv: control versus over-expression of LEF1.

19401, Genes up-regulated in the *in vitro* follicular dendritic cells from peripheral lymph nodes: non-stimulated versus tretinoin and Pam2CSK4 (96h).

37301 Genes down-regulated in lymphoid primed multipotent progenitors versus RAG2 knockout NK cells.

43260, Genes up-regulated in tumor-infiltrating CD8 T cells: BTLA+ versus BTLA- .

^2^Noramlized enrichment score, positive NES or negative NES suggests that the genes in the set are upregulated or downregulated, respectively.

^3^Normalized p value.

^4^False discovery rate q-value.

### Multivariable analysis of predictive biomarkers:

**Figure 4:**
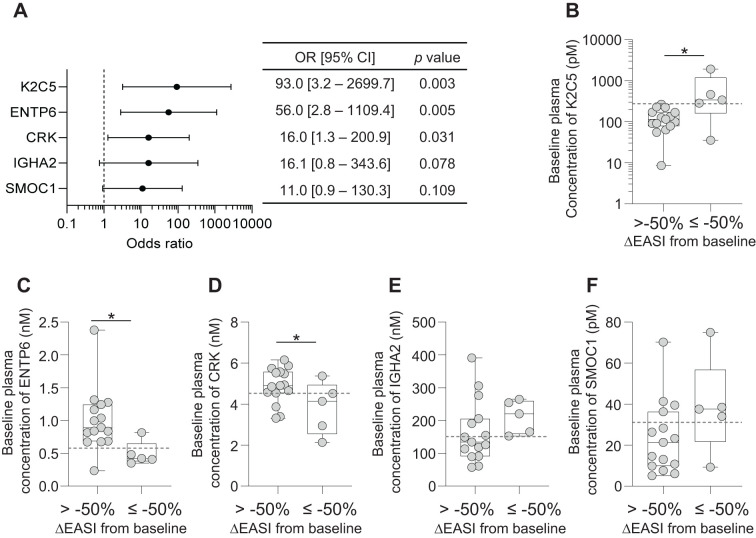
Baseline plasma expression levels of 5 biomarkers in EASI50 responders and non-responders. **(A)** Forest plot of odds ratios (OR) and 95% confidence intervals (CI) for the five biomarkers, providing their predictive value for treatment response. Each biomarker is displayed along with its corresponding OR and CI, highlighting the strength of association with clinical outcomes. **(B-F)** Box plots of baseline expression levels of the 5 biomarkers across EASI50 responders (ΔEASI from baseline≤ -50%) and non-responders (ΔEASI from baseline > -50%). **(B)** K2C5, **(C)** ENTP6, **(D)** CRK, **(E)** IGHA2 and **(F)** SMOC1. Dashed lines indicate the cut-off point values for each biomarker, which may serve as thresholds for predicting treatment response. The boxes represent the interquartile range, while the whiskers extend to the minimum and maximum values. Individual data points are also shown to illustrate variability within each group. Statistical significance is indicated with *p < 0.05, determined by Student’s *t*-test.

Significant odds ratios (ORs) were observed for three biomarkers (K2C5, ENTP6 and CRK), but not for IGHA2 and SMOC1 (**Figure 4**). Stratification based on K2C5 levels showed the highest OR (93.0, 95% CI [3.2–2699.7], p = 0.003), suggesting the strongest association between baseline K2C5, ENTP6 and CRK levels and favorable clinical outcomes.

Baseline plasma levels of K2C5, ENTP6, and CRK showed a significant association with EASI50 response (**Figures 4B–D**). Notably, baseline K2C5 levels were significantly higher in patients achieving EASI50 response, indicating a strong positive correlation between high K2C5 levels and favorable outcomes (**Figure 4B**). Conversely, baseline ENTP6 and CRK levels were significantly lower in EASI50 responders to Nu0.5, suggesting an inverse relationship between baseline levels and clinical responsiveness (**Figures 4C, D**). IGHA2 and SMOC1 did not show statistically significant differences between responders and non-responders (p > 0.05; **Figures 4E, F**). Overall, these findings suggest that baseline plasma levels of K2C5, ENTP6, and CRK are robust predictors of patient clinical response assessed by EASI50.

**Figure 5:**
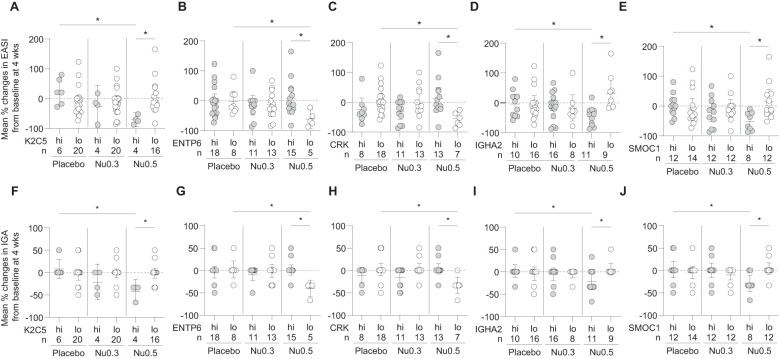
The relationship between baseline biomarker levels and treatment response. **(A-E)** The percentage change in EASI **(A-E)** and IGA **(F-J)** scores at week 4 from baseline is presented for participants stratified by baseline levels of the 5 biomarkers: **(A)** K2C5, **(B)** ENTP6, **(C)** CRK, **(D)** IGHA2, and **(E)** SMOC1. Bars represent mean percentage changes ± 95% confidence intervals (CI). Statistical significance is indicated with *p < 0.05, determined by Student’s *t*-test.

Baseline biomarker level analysis revealed distinct patterns in clinical outcomes, measured by percentage changes in EASI and IGA scores, across the placebo, Nu0.3, and Nu0.5 treatment groups (**Figure 5**). Patients were stratified into “high” (hi) and “low” (lo) biomarker groups based on cut-off points for baseline concentrations. For K2C5, patients in the “hi” group demonstrated significantly greater improvements in EASI and IGA scores with Nu0.5 treatment compared to the “lo” group (p < 0.05, **Figures 5A, F**). No clinical effects were observed in the placebo group and Nu0.3 group, regardless of stratification. Nu0.5 significantly improved outcomes compared to placebo in the “hi” group (p < 0.05, **Figures 5A, F**). For ENTP6, an inverse relationship was observed, with the “lo” group showing greater improvements in EASI and IGA scores with Nu0.5 (p < 0.05, **Figures 5B, G**), highlighting ENTP6 as a negative predictive biomarker. Similarly, CRK levels were associated with poorer outcomes in the “hi” group, while the “lo” group responded better to Nu0.5 (p < 0.05, **Figures 5C, H**). For IGHA2, slightly better clinical improvement was observed in the “hi” group following Nu0.5 treatment (p < 0.05, **Figures 5D, I**). SMOC1 showed significantly better outcomes in the “hi” group with Nu0.5 compared to the “lo” group (p < 0.05, **Figures 5E, J**). Across all biomarkers, no significant differences were observed between the “hi” and “lo” groups in the placebo arm (**Figures 5A–E**). Nu0.3 did not show clinical benefits in any subgroup (**Figures 5**).

Overall, K2C5^hi^, ENTP6^lo^, CRK^lo^, IGHA2^hi^, and SMOC1^hi^ participants showed clinical improvement (ΔEASI and ΔIGA) in response to Nu0.5 significantly better than placebo after stratification using these biomarkers (**Table 2**). Notably, K2C5^hi^ participants, representing 20% of the BAS population, showed the most substantial clinical improvement, with a ΔEASI (Nu0.5 vs. placebo) of -94.1 [95% CI: -146.1 to -42.0, p=0.003] and a ΔIGA (Nu0.5 vs. placebo) of -50.0 [95% CI: -78.4 to -21.6, p=0.004]. Baseline K2C5 levels could predict clinical response to Nu0.5 with high sensitivity and specificity (AUC = 0.953, p = 0.006). The positive Prediction value (PPV) and negative prediction value (NPV) of K2C5 were 1.0 and 0.938, respectively. Among these five biomarkers, CRK^lo^ participants, representing the largest proportion of the BAS (54.3%), demonstrated significant clinical improvement with a ΔEASI (Nu0.5 vs. placebo) of -61.3 [95% CI: -99.9 to -22.8, p=0.003] and a ΔIGA (Nu0.5 vs. placebo) of -35.2 [95% CI: -58.2 to -12.1, p=0.004]. Baseline CRK levels could predict clinical response to Nu0.5 with high sensitivity and specificity (AUC = 0.923, p = 0.002). The PPV and NPV of CRK were 1.0 and 0.846, respectively (**Table 4**).

**Figure 6:**
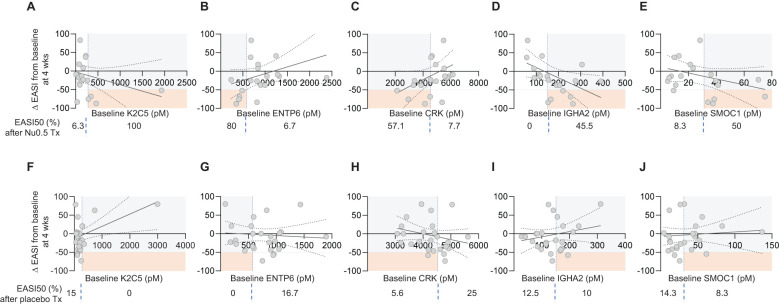
Correlation between baseline plasma biomarker levels and EASI Score percentage change. **(A-E)** The correlation between baseline plasma levels of individual biomarkers (K2C5, ENTP6, CRK, IGHA2, and SMOC1) and the percentage change in EASI scores from baseline at week 4 for participants in the Nu0.5 treatment group. Each plot illustrates the relationship between biomarker levels and ΔEASI from the baseline, with vertical dashed lines indicating biomarker cut-off points. **(F-J)** Corresponding plots for the placebo group are shown, allowing for comparison of the correlation between baseline biomarker levels and EASI score changes in participants. Horizontal dashed lines represent the EASI50 response threshold, delineating participants who achieved a clinically significant improvement (ΔEASI ≤ -50%). Orange squares highlight EASI50 responders among those predicted to respond based on biomarker cut-off points. Percentages displayed below each panel indicate the proportion of participants achieving ΔEASI ≤ -50% among those predicted to respond based on their baseline biomarker levels. Logistic probability curves (solid oblique lines) with 95% confidence intervals (dotted oblique lines) are fitted to the data, providing a statistical model of the relationship between biomarker levels and clinical response (p > 0.05).

The relationship between baseline biomarker levels and EASI score changes after 4 weeks were assessed (**Figure 6**). Although statistically insignificant (p > 0.05), positive or negative trend was observed in the Nu0.5 group with baseline levels of biomarkers associated with reductions in EASI scores (**Figure 6**), but not in the placebo group. All K2C5^hi^ patients showed ΔEASI ≤ -50% from the baseline. Conversely, 80% and 57.1% of ENTP6^lo^ patients and CRK^lo^ patients showed ΔEASI ≤ -50% from the baseline, respectively. IGHA2 and SMOC1 exhibited a slight negative trend that warrants further investigation (**Figures 6D, E**). The 45.5% and 50% of IGHA2^hi^ patients and SMOC1^hi^ patients showed ΔEASI ≤ -50% from the baseline, respectively. Although positive- or negative trends were not statistically significant, they align with the stratified analysis results, suggesting their potential as predictive biomarkers. No significant associations were observed in the placebo group (**Figures 6F-J**). This indicates that these trends are specific to Nu0.5 treatment, highlighting the potential utility of K2C5, ENTP6, and CRK in predicting treatment outcomes.

Baseline plasma concentrations of K2C5, ENTP6, CRK, IGHA2, and SMOC1 did not statistically differ significantly between the treatment groups (placebo, Nu0.3, and Nu0.5) (**Supplementary Figure S5**). Median and interquartile ranges were comparable between groups, and baseline biomarker levels showed balanced variability without systematic shifts. These results demonstrate that biomarker levels at baseline were well balanced between treatment groups, ensuring that the observed treatment-related outcomes after stratification were not confounded by pre-treatment imbalances in biomarker concentrations.

### Likelihood ratio analysis for combination of two biomarkers:

**Figure 7:**
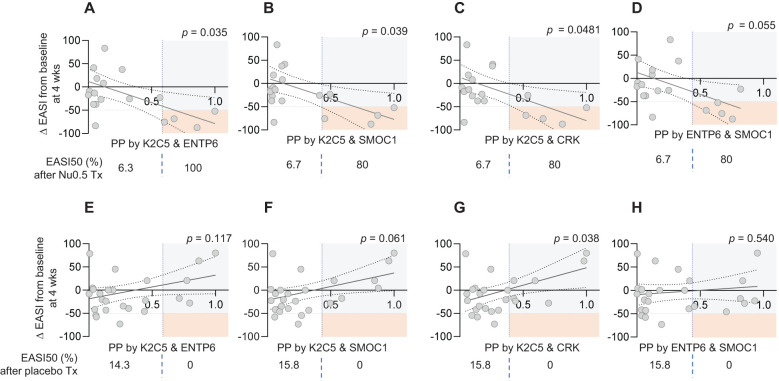
Correlation between predicted probability (pp) from two-biomarker combinations and percentage change in EASI score. The pp was calculated using logistic regression analysis based on baseline levels of two biomarkers. **(A-D)** The pp derived from combinations of two biomarkers and the percentage change in EASI scores at week 4 for participants in the Nu0.5 treatment group. The biomarker pairs analyzed include: **(A)** K2C5 and ENTP6, **(B)** K2C5 and SMOC1, **(C)** K2C5 and CRK, **(D)** ENTP6 and SMOC1. Vertical dashed lines indicate the cut-off points for pp, while horizontal dashed lines represent the EASI50 response threshold, delineating participants who achieved a clinically significant improvement (ΔEASI ≤ -50%). Orange squares highlight EASI50 responders among those predicted to respond based on pp cut-off points. **(E-H)** Corresponding placebo group analysis. Logistic probability curves (solid oblique lines) with 95% confidence intervals (dotted oblique lines) are fitted to the data, providing a statistical model of the relationship between biomarker levels and clinical response. P-values from logistic regression analysis are displayed at the top of each panel, indicating the significance of these correlations.

A logistic regression-based prediction model was developed to assess the association between the combination of baseline plasma levels of 2 biomarkers and clinical responsiveness determined by EASI score change after treatment with Nu0.5. Logistic regression analysis revealed that combinations of two biomarkers significantly predicted clinical response to Nu0.5 treatment (**Figure 7**). Predicted probabilities (pp) were calculated using logistic regression analysis. The pp of clinical response after treatment with Nu0.5 showed a negative correlation with ΔEASI, with the strongest correlation observed for the “K2C5 and ENTP6” combination (p = 0.035, **Figure 7A**), followed by “K2C5 and SMOC1” (p = 0.039, **Figure 7B**) and “K2C5 and CRK” (p = 0.048, **Figure 7C**). The “ENTP6 and SMOC1” combination exhibited a negative trend with borderline significance (p = 0.055). These findings highlight K2C5 as a central biomarker, with K2C5 combinations consistently demonstrating predictive value for clinical response. Patients with higher pp based on these biomarker combinations exhibited greater clinical improvement, supporting the utility of two-biomarker-based stratification for optimizing Nu0.5 treatment outcomes.

Using the pp threshold derived from the “K2C5 and ENTP6” combination for stratification, 100% of participants responded to Nu0.5 (ΔEASI from baseline ≤ -50%, **Figure 7A**), while 0% responded in the placebo group (**Figure 7E**). Using pp thresholds derived from other combinations, such as “K2C5 and SMOC1” (**Figure 7B**) or “K2C5 and CRK” (**Figure 7C**), 80% of participants responded to Nu0.5 (ΔEASI from baseline ≤ -50%), with no responses observed in the placebo group (**Figures 7F, G**). These results underscore the potential of pp thresholds for accurate patient stratification and effective prediction of treatment response.

After stratification with the combination of 2 biomarkers, predictive metrics such as the percentage of responders in each stratified group (%BAS), ROC-AUC, PPV, NPV and OR were calculated for each biomarker combination (**Table 5**). Biomarker combinations showing significant improvement in both EASI and IGA scores and demonstrating high predictive metrics (ROC-AUC, PPV, and NPV) were considered strong predictors of clinical response. Briefly, the combination of “K2C5^hi^ or ENTP6^lo^” exhibited the greatest improvement in ΔEASI (-65.3, 95% CI: [-113.0, -17.7], p = 0.011) and ΔIGA (-41.9, 95% CI: [-65.3, -18.4], p = 0.002) and displayed high predictive metrics, including ROC-AUC 0.893 (p = 0.01), PPV 0.8 and NPV 0.933 (**Table 5**). The combination of “K2C5^hi^ or ENTP6^lo^” exhibited the highest OR of 56.0 (95% CI: 2.8–1109.4, p = 0.005), indicating a strong association with a higher likelihood of response. Similarly, combinations of “K2C5^hi^ or CRK^lo^” (OR: 16.0, p = 0.031) also significantly predicted clinical response, albeit with lower odds ratios. These findings highlight K2C5 as a crucial biomarker for predicting clinical response to Nu0.5, particularly in combination with ENTP6, or CRK, supporting its utility in patient stratification and personalized treatment strategies.

**Table 5:** Clinical improvement of patients after stratification with two biomarkers.

Patient groups stratified with	ΔEASI^1^ Mean [95% CI]	*p* value^2^	ΔIGA^3^ Mean [95% CI]	*p* value^2^	% BAS^4^	ROC^5^		PPV^6^	NPV^7^	OR^8^ [95% CI]	*p* value
						AUC	*p* value				
K2C5^hi^ or ENTP6^lo^	-65.3 [-113.0 – -17.7]	0.011	-41.9 [-65.3 – -18.4]	0.002	41.4	0.893	0.01	0.800	0.933	56.0 [2.8 – 1109.4]	0.005
K2C5^hi^ or SMOC1^hi^	-55.7 [-87.1 – -24.2]	0.001	-30.8 [-51.9 – -9.8]	0.006	51.4	0.904	0.0024	0.889	0.818	28.1 [1.3 – 619.9]	0.023
K2C5^hi^ or CRK^lo^	-61.3 [-99.9 – -22.8]	0.003	-35.2 [-58.2 – -12.1]	0.004	58.6	0.923	0.0023	1.000	0.846	16.0 [1.3 – 200.9]	0.031
K2C5^hi^ or IGHA2^hi^	-43.9 [-77.2 – -10.6]	0.012	-26.3 [-47.0 – -5.7]	0.015	58.6	0.950	0.0007	0.909	1.000	16.1 [0.8 – 343.6]	0.078
ENTP6^lo^ or SMOC1^hi^	-44.8 [-73.5 – -16.0]	0.004	-28.6 [-49.0 – -8.2]	0.008	62.9	0.904	0.0024	0.889	0.818	28.1 [1.3 – 619.9]	0.023
ENTP6^lo^ or IGHA2^hi^	-36.4 [-67.8 – -5.0]	0.025	-26.3 [-47.0 – -5.7]	0.015	62.9	0.950	0.0007	0.909	1.000	16.1 [0.8 – 343.6]	0.078
ENTP6^lo^ or CRK^lo^	-53.9 [-89.0 – -18.7]	0.004	-35.0 [-55.2 – -14.8]	0.001	64.3	0.906	0.0026	1.000	0.833	11.0 [0.9 – 130.3]	0.109
CRK^lo^ or SMOC1^hi^	-52.7 [-85.4 – -20.0]	0.003	-26.7 [-46.4 – -6.9]	0.010	72.9	0.940	0.0009	0.900	0.900	21.0 [1 – 453.9]	0.069
CRK^lo^ or IGHA2^hi^	-48.1 [-80.0 – -16.2]	0.004	-22.9 [-42.8 – -3.0]	0.026	72.9	0.950	0.0007	0.909	1.000	16.1 [0.8 – 343.6]	0.078

^1^ΔEASI [Nu0.5 - Placebo]; ^2^Student’s *t*-test [Nu0.5 vs. Placebo]; ^3^ΔIGA [Nu0.5 - Placebo]; ^4^The percentage of participants included in the biomarker analysis set after stratification; ^5^Receiver operating characteristic; ^6^positive prediction value; ^7^negative prediction value, ^8^odd ratio.

In comparison to the placebo group, patients stratified by the three biomarkers “K2C5^hi^, CRK^lo^, or SMOC1^hi^,” representing the largest proportion of BAS at 74.3%, demonstrated clinical improvements, achieving a -52.7% change in EASI scores and a -26.7% change in IGA scores (p < 0.05; **Supplementary Table 5**). These results are further supported by **Supplementary Figure S6**, which visualizes the relationship between predicted probabilities and EASI score changes based on three-biomarker combinations in the Nu0.5 and placebo groups.

### Safety

The safety of NuGel was evaluated over a 4-week treatment period for both low-dose (Nu0.3) and high-dose (Nu0.5) formulations. Among the 80 participants included in the SAS, 10–15% of participants receiving Nu0.3 and Nu0.5 reported treatment-emergent adverse events (TEAEs, **Table 6**). Specifically, TEAEs were reported in 8 participants (10.00%, 12 events). In the placebo group, 1 individual (3.70%) reported 1 mild event. In the Nu0.3 group, 4 individuals (14.81%) reported 8 mild events. In the Nu0.5 group, 2 individuals (7.69%) reported 2 mild events, and 1 individual (3.85%) reported 1 moderate event. Overall, 11 events (91.67%) were classified as mild, occurring in 7 participants (8.75%), while 1 event (8.33%) was classified as moderate, occurring in 1 participant (1.25%). The number of cases was insufficient to analyze severity differences between treatment groups, but no dose-dependent trend in severity was observed. The causality of all adverse events was assessed as “not related.” Headache was the most common adverse event, reported in 2 cases (2 participants). No cases of adverse drug reactions (ADRs) were reported. Furthermore, no serious adverse events, serious adverse drug reactions, events leading to permanent discontinuation, or dropouts due to adverse events were observed. Most laboratory test parameters showed minimal changes from baseline, with no clinically significant abnormalities or adverse findings reported. Similarly, no clinically significant abnormalities in vital signs or transitions to clinically meaningful abnormal values were observed. Finally, physical examinations and electrocardiogram (ECG) evaluations revealed no clinically significant abnormal findings after treatment. In conclusion, the comprehensive assessment of adverse events, drug reactions, laboratory test results, vital signs, physical examinations, and ECG findings suggests that NuGel demonstrates a favorable safety profile at both low (0.3%) and high (0.5%) doses.

**Table 6:** Summary of TEAEs^1^.

System Organ Class	Placebo (N=27)		Nu0.3 (N=27)		Nu0.5 (N=26)		Total (N=80)	
	n (%)	[events]	n (%)	[events]	n (%)	[events]	n (%)	[events]
Infections and infestations	0 (0.00)	[0]	1(3.70)	[1]	1(3.85)	[1]	2(2.50)	[2]
Acute sinusitis	0 (0.00)	[0]	0 (0.00)	[0]	1(3.85)	[1]	1(1.25)	[1]
Nasopharyngitis	0 (0.00)	[0]	1(3.70)	[1]	0 (0.00)	[0]	1(1.25)	[1]
Injury, poisoning and procedural complications	1(3.70)	[1]	0 (0.00)	[0]	1(3.85)	[1]	2(2.50)	[2]
Facial bones fracture	0 (0.00)	[0]	0 (0.00)	[0]	1(3.85)	[1]	1(1.25)	[1]
Thermal burn	1(3.70)	[1]	0 (0.00)	[0]	0 (0.00)	[0]	1(1.25)	[1]
Musculoskeletal and connective tissue disorders	0 (0.00)	[0]	1(3.70)	[1]	1(3.85)	[1]	2(2.50)	[2]
Arthralgia	0 (0.00)	[0]	0 (0.00)	[0]	1(3.85)	[1]	1(1.25)	[1]
Myalgia	0 (0.00)	[0]	1(3.70)	[1]	0 (0.00)	[0]	1(1.25)	[1]
Nervous system disorders	0 (0.00)	[0]	2(7.41)	[2]	0 (0.00)	[0]	2(2.50)	[2]
Headache	0 (0.00)	[0]	2(7.41)	[2]	0 (0.00)	[0]	2(2.50)	[2]
General disorders and administration site conditions	0 (0.00)	[0]	1(3.70)	[2]	0 (0.00)	[0]	1(1.25)	[2]
Chills	0 (0.00)	[0]	1(3.70)	[1]	0 (0.00)	[0]	1(1.25)	[1]
Pyrexia	0 (0.00)	[0]	1(3.70)	[1]	0 (0.00)	[0]	1(1.25)	[1]
Gastrointestinal disorders	0 (0.00)	[0]	1(3.70)	[1]	0 (0.00)	[0]	1(1.25)	[1]
Dyspepsia	0 (0.00)	[0]	1(3.70)	[1]	0 (0.00)	[0]	1(1.25)	[1]
Respiratory, thoracic and mediastinal disorders	0 (0.00)	[0]	1(3.70)	[1]	0 (0.00)	[0]	1(1.25)	[1]
Cough	0 (0.00)	[0]	1(3.70)	[1]	0 (0.00)	[0]	1(1.25)	[1]
Summary								
Patients with ≥1 adverse event, *n* (%)	1(3.70)	[1]	4(14.81)	[8]	3(11.54)	[3]	8(10.00)	[12]
Serious adverse event (SAE), *n* (%)	0 (0.00)	[0]	0 (0.00)	[0]	0 (0.00)	[0]	0 (0.00)	[0]

^1^TEAE, treatment-emergent adverse event.

## Discussion

This study explores the potential of biomarker-based precision medicine in managing atopic dermatitis (AD), a condition with considerable heterogeneity in clinical presentation and treatment response. By identifying five candidate biomarkers—K2C5, ENTP6, CRK, IGHA2, and SMOC1—associated with the efficacy of Nu0.5, this work aims to contribute to the framework for patient stratification and personalized therapeutic approaches. While these findings are promising, further research is essential to substantiate their clinical utility.

The identified biomarkers may offer insights into the pathophysiology of AD and the mechanisms underlying Nu0.5 efficacy. For instance, elevated expression of type II keratin 5 (K2C5^hi^) was associated with improved treatment outcomes, suggesting a potential role for K2C5 in maintaining epidermal integrity during skin repair and regeneration (39–42). Previous studies have highlighted its immunological significance in preserving epithelial barrier functions and regulating immune cell recruitment to the epidermis (39–42). However, the precise mechanisms through which K2C5 influences AD pathogenesis and efficacy of Nu0.5 require further investigation.

Similarly, lower levels of ENTP6 correlated with better clinical outcomes, possibly due to reduced dysregulation of ATP metabolism and the modulation of the ATP-P2X7R-NLRP3 inflammasome axis (24). ENTP6, which is involved in nucleotide metabolism and purinergic signaling, may indirectly influence skin inflammation by inflammasomal activation (43). Although ENTP6’s direct role in AD remains uncertain, its involvement in purinergic signaling pathways linked to inflammasomal activation may provide a potential mechanistic link to AD pathophysiology and efficacy of Nu0.5 (44).

CRK, an adaptor protein involved in immune cell migration, also emerged as a candidate biomarker (45). Its role in regulating intracellular signaling pathways critical for T-cell migration and keratinocyte dynamics suggests a potential connection to inflammatory processes in AD and Nu0.5 efficacy (45–48). However, further studies are necessary to establish its relevance in this context.

The association of SMOC1 with Nu0.5 efficacy highlights its potential involvement in modulating inflammatory microenvironments and influencing keratinocyte behavior through TGF-β and calcium signaling pathways (49, 50). Additionally, SMOC1 is involved in tissue repair and regeneration processes and may affect keratinocyte behavior through calcium signaling pathways critical for differentiation and skin barrier function (51). While the exact role of SMOC1 in AD remains unclear, its dysregulation in other inflammatory skin conditions supports the need for additional research (51, 52).

The observed seasonal variation in treatment efficacy, with greater improvements during fall and winter, underscores the influence of environmental factors, such as cold weather and low humidity, on AD pathogenesis (53, 54). This finding emphasizes the importance of accounting for external variables in the design of clinical trials and treatment strategies targeting inflammasome pathways in keratinocytes and immune cells.

While current therapies for AD, including PDE4 inhibitors, JAK inhibitors, and biologics, have demonstrated efficacy, limitations such as long-term safety concerns remain (5, 55–59). Integrating biomarker-guided patient selection into clinical practice might offer a promising approach to addressing these challenges by reducing adverse outcomes and enabling targeted treatment. However, further validation in larger, more diverse cohorts is necessary to confirm these preliminary findings. Mechanistic studies investigating the interplay between Nu0.5, GPCR19, and the identified biomarkers could deepen our understanding of molecular pathways and guide the development of next-generation precision therapies. Moreover, the development of cost-effective diagnostic tools will be critical for translating biomarker-based approaches into routine clinical practice. These findings may contribute to the ongoing efforts to advance personalized therapeutic strategies while laying a foundation for future research into novel targeted interventions for AD.

### Limitations and future directions

This study has several limitations that must be considered. The relatively small cohort size limited the statistical power and may have affected the generalizability of the findings. Additionally, the short 4-week treatment duration may not fully capture the long-term efficacy and safety of Nu0.5, particularly for chronic management. Moreover, the study did not explore dose escalation, which might provide further insights into the dose-response relationship and optimize therapeutic outcomes.

Future studies should prioritize larger, multicenter cohorts with extended follow-up periods to validate these findings and assess the durability of NuGel’s clinical benefits. Mechanistic research focusing on the identified biomarkers and their associated pathways is also essential to elucidate their roles in AD pathogenesis and Nu0.5 efficacy. Such efforts could validate the clinical utility of these biomarkers and identify novel therapeutic targets, advancing our understanding of AD biology and paving the way for innovative treatments.

## Conclusion

This study highlights the potential of biomarker-based stratification to optimize treatment outcomes in AD. Nu0.5 demonstrated notable efficacy in biomarker-defined subgroups, particularly in patients with specific baseline profiles, suggesting its utility as a personalized therapeutic agent. Its favorable safety profile further supports its potential clinical application. These findings, while preliminary, provide a basis for advancing precision medicine in AD management. Continued research is needed to confirm these results and further refine tailored therapeutic strategies to improve outcomes for individuals affected by this chronic inflammatory condition.

### Transparency, rigor, and reproducibility summary

The study design and analysis plan were preregistered on January 13, 2020 at https://clinicaltrials.gov/study/NCT04530643?term=NCT04530643&rank=1. Prespecified sample size was 75. All subjects were assigned to 0.3% NuGel, 0.5% NuGel or placebo using a random number generator, yielding groups that did not differ in baseline characteristics. Eighty participants were randomized, and primary outcomes were assessed in 79 participants (FAS) due to one participant having an incomplete assessment. All primary outcomes were assessed by blinded investigators who could not guess group assignments better than chance. Key inclusion criteria were evaluated by investigators. Dermatologists assessed clinical outcomes. An experienced researcher conducted statistical analysis for the clinical trial.

## Data Availability

De-identified data from this study are not available in a public archive. The original contributions presented in the study are included in the article/**Supplementary Material**. Further inquiries can be directed to the corresponding author.

## Supplementary material

Supplementary Figure 1 Clinical Trial Design. The clinical trial was a randomized, double-blind, placebo-controlled, three-arm study conducted over a 4-week period. Participants attended four clinic visits: Visit 1 (screening): conducted -21 to -1 days prior to baseline (day 1), this visit assessed eligibility criteria through screening procedures. Visit 2 (baseline): occurred on day 1, marking the trial’s initiation. Baseline assessments included physical examinations and initial blood sampling. Visit 3 (midpoint): scheduled for day 15 (± 3 days), this visit focused on interim evaluations of safety and efficacy. Visit 4 (study end): Conducted on day 29 (± 3 days), concluding the trial with final assessments of trial outcomes, including physical examinations and blood sampling.

Supplementary Figure 2 Workflow for target selection and ranked analysis of target protein levels. **(A)** The workflow begins with the mixing of 802 stable isotope standard (SIS) peptides with pooled plasma from 70 patients. Quantitative analysis was conducted using MRM-MS with a 6495 QQQ system. Assay quality was validated by ensuring that the intensity of peaks (either SIS or endogenous) exceeded 100 counts, SIS peaks co-eluted with corresponding endogenous peptides, and elution peaks displayed no skewed profiles. After validation, 502 targets were selected for further analysis, and linearity was assessed using variable amounts of SIS peptides (50–400 fmol). Targets were included when the predictive accuracy ratio (PAR) fell between 0.1 and 1, particularly when PAR approached 1. Additional testing reduced this number to 469 targets, which were validated for dynamic range of quantification, technical variation across different sites, and predictive power for clinical responses. The predictive power for EASI50 responses was determined by an area under the receiver operating characteristic curve (AUROC) threshold = 0.7 in ROC analysis following stratification using the highest Youden Index cut-off point based on plasma concentration. Biomarkers were excluded from analysis if their AUROC was less than 0.7. Clinical responses were assessed by EASI50 after treatment with Nu0.5. Technical variation was validated through PCA across individual hospitals and individual SIS samples. Of the 469 targets, 42 DEPs were identified based on AUROC > 0.7. To further evaluate predictive power, the statistical significance (p < 0.05) of changes in EASI scores (ΔEASI) and IGA scores (ΔIGA) between the Nu0.5 and placebo groups was determined after stratifying patients based on cut-off points derived from AUROC analysis and the highest Youden index. A final subset of five DEPs with p-values < 0.05 for both ΔEASI (Nu0.5 vs. placebo) and ΔIGA (Nu0.5 vs. placebo) was selected. **(B)** Ranked analysis of target protein levels: The graph ranks target proteins by their plasma levels on the x-axis and plots these levels on a log10 scale on the y-axis. Key markers such as ALBU (albumin) and IGHG1 (immunoglobulin gamma 1) are labeled at the top of the distribution. The upper 5% region represents highly abundant plasma proteins, while the majority of proteins fall within the central dynamic range of plasma levels, reflecting lower abundance proteins relevant for biomarker discovery. The lower 5% region represents very low-abundance targets that may be challenging to detect or quantify reliably. The “90% of targets analyzed” zone focuses on biomarker discovery, where proteins show intermediate abundance, offering higher specificity for disease states. Starting with a broad peptide panel and filtering down based on assay performance and biological relevance ensures that only the most reliable and clinically significant DEPs are included. The ranked peptide data demonstrate effective selection of proteins across a dynamic range, prioritizing those with sufficient detectability and clinical relevance.

Supplementary Figure 3 Percent change in EASI and IGA scores at week 2 compared to baseline in various patient groups. **(A, B)** FAS analysis of EASI **(A)** and IGA **(B)** scores. **(C, D)** PPS analysis of EASI **(C)** and IGA **(D)** scores. **(E, F)** Analysis in patients with moderate to severe disease at baseline (**(E)** 7 < EASI ≤ 21; F, IGA = 3). **(G, H)** Analysis in patients enrolled from September 2020 to February 2021 for EASI **(G)** and IGA **(H)** scores. Bars represent mean ± 95% CI. (mean values at the top of bars), n = number of patients. *p < 0.05; _†_0.05 < p < 0.09. EASI, Eczema Area and Severity Index; IGA, Investigator’s Global Assessment.

Supplementary Figure 4 PCA analysis and plasma protein concentrations across five hospitals. **(A)** The PCA plot demonstrates the variability in plasma biomarkers across samples collected from five hospitals, revealing no apparent clustering by site. This observation suggests minimal site-related biases and supports the robustness of the data collection and processing methods employed in the study. **(B)** This boxplot illustrates the plasma concentrations of target proteins across 70 patients, grouped by the site of collection from five hospitals. Each box represents the plasma protein concentrations for individual patients, categorized by site: cyan for site 1, blue for site 2, red for site 3, yellow for site 4, and green for site 5. The plasma concentrations are plotted on a log10 scale, with boxes representing the interquartile range (IQR) and the median line displayed within each box. Whiskers extend to 1.5 × IQR from the box, while dots represent outliers beyond the whiskers. The distributions of plasma protein concentrations are similar across all five sites, as indicated by overlapping IQRs and median values. This similarity suggests a lack of significant site-specific variability in plasma protein measurements. Numerous outliers are present across patients, likely reflecting biological variability in protein levels or assay noise for certain proteins. Notably, these outliers are uniformly distributed across sites, further supporting consistency in data quality. The log10 scale captures a wide dynamic range of plasma protein levels, spanning approximately five orders of magnitude. This range is consistent across sites, indicating reproducibility in detection limits and assay sensitivity. Within each site, the boxplots show a relatively uniform distribution of protein levels, with no dramatic differences between individual patients. This consistency suggests minimal site-specific biases in patient selection or sample handling. Overall, the consistent distributions across sites indicate that hospital-specific factors do not significantly affect protein measurements. The dynamic range of protein levels highlights the assay’s capability to detect both high- and low-abundance proteins, making it well-suited for biomarker discovery. The similar distributions within each site further indicate minimal variability in patient populations recruited from different hospitals.

Supplementary Figure 5 Baseline plasma concentrations of 5 biomarkers across three groups: placebo, Nu0.3, and Nu0.5. **(A)** The boxplot illustrates the baseline concentrations of K2C5 (pM), spanning a wide range from approximately 10 pM to 10,000 pM on a logarithmic scale. Median concentrations are comparable across the three groups, indicating no significant differences. However, variability is high, with several outliers present in all groups. **(B)** The concentrations of ENTP6 (nM) range from about 0.5 nM to 2 nM. Variability is consistent across groups, with some outliers observed. **(C)** The baseline concentrations of CRK (nM) range from approximately 2 nM to 8 nM. Median values remain consistent across the groups, with no notable differences observed. The distribution of CRK concentrations is narrower compared to other biomarkers. **(D)** The concentrations of IGHA2 (nM) range from around 50 nM to 500 nM, showing a broad distribution but similar median values across groups. **(E)** SMOC1 (pM) concentrations range from about 10 pM to 150 pM, with median values comparable across the three groups.

Supplementary Figure 6 Correlation between pp calculated from baseline plasma levels of 3 biomarkers and percentage change in EASI Score. **(A)** Forest plot of odds ratios (OR) and 95% confidence intervals (CI). This forest plot illustrates the ORs for combinations of three biomarkers in predicting treatment response. Each biomarker combination is represented, highlighting its predictive value for achieving a clinically significant improvement in EASI scores. **(B-E)** Correlation analysis in the Nu0.5 treatment group: The correlation between pp derived from the logistic regression model and the mean percentage change in EASI score is presented for the following three-biomarker combinations: **(B)** K2C5, ENTP6, and SMOC1, **(C)** K2C5, ENTP6, and CRK, **(D)** K2C5, CRK, and SMOC1, **(E)** ENTP6, CRK, and SMOC1. Vertical dashed lines indicate the cut-off points for pp, while horizontal dashed lines denote a ΔEASI of -50%, serving as the threshold for clinical responders. Among patients predicted as responders based on pp cut-off levels, those achieving ΔEASI ≤ -50% are highlighted in orange. **(F-I)** Corresponding analysis in the placebo group: This section presents similar analyses for the placebo group using the same biomarker combinations, allowing for comparison of treatment effects. Logistic probability curves (solid oblique lines) with 95% confidence intervals (dotted oblique lines) were generated using a logistic regression model fitted to the pp and clinical response (ΔEASI from baseline). Circles represent predicted data points from individual patients. P-values for logistic regression analysis are displayed at the top of each panel, indicating the significance of these correlations in Nu0.5 groups. In placebo groups, p > 0.05 for all combinations.

## Glossary

AD: atopic dermatitis
AEs: adverse events
AN32B: Acidic leucine-rich nuclear phosphoprotein 32 family member B
ANOVA: analysis of variance
AUC: area under the curve
BAS: biomarker analysis set
BSA: body surface area
CRK: Adapter molecule crk
DAMPs: damage-associated molecular patterns
EASI: Eczema Area and Severity Index
ENPL: Endoplasmin
ENTP6: Ectonucleoside triphosphate diphosphohydrolase 6
FA11: Coagulation factor XI
FABP5: fatty-acid-binding protein
FAS: full analysis set
GPCR19: G-protein coupled receptor 19
hBD-2: human beta-defensin-2
IGA: Investigator’s Global Assessment
IGHA2: Immunoglobulin heavy constant alpha 2
iNOS: inducible nitric oxide synthase
IPO7: Importin-7
JAKi: Janus kinase inhibitors
K2C5: Keratin, type II cytoskeletal 5
MMP8/9: matrix metalloproteinases 8/9
MRM-MS: multiple reaction monitoring mass spectrometry
MUC5A: Mucin-5AC
NOS2: nitric oxide synthase 2
NRS: Numerical Rating Scale
PAMPs: pathogen-associated molecular patterns
PCA: principal component analysis
PPS: per-protocol set
PP-NRS: Peak Pruritus Numerical Rating Scale
QqQ: triple quadrupole
ROC: receiver operating characteristic
SAS: safety analysis set
SEM: standard error of mean
SIS: stable isotope-labeled standard
SMOC1: SPARC-related modular calcium-binding protein 1
TDCA: taurodeoxycholate
TEAEs: treatment-emergent adverse events
Th: T helper.
