# Supplementary material for: A phase 2a double-blind, placebo-controlled, randomized clinical trial evaluating the efficacy and safety of NuGel, a novel topical GPCR19-mediated inflammasome inhibitor, in patients with mild to moderate atopic dermatitis: a proof-of-concept study with Post-hoc biomarker analysis
Source: Front Immunol. 2025 May 19;16:1560447. doi: 10.3389/fimmu.2025.1560447 (PMC12127193; doi:10.3389/fimmu.2025.1560447)
Supplement: Supplementary Figure 1 — Clinical Trial Design. The clinical trial was a randomized, double-blind, placebo-controlled, three-arm study conducted over a 4-week period. Participants attended four clinic visits: Visit 1 (screening): conducted -21 to -1 days prior to baseline (day 1), this visit assessed eligibility criteria through screening procedures. Visit 2 (baseline): occurred on day 1, marking the trial’s initiation. Baseline assessments included physical examinations and initial blood sampling. Visit 3 (midpoint): scheduled for day 15 (± 3 days), this visit focused on interim evaluations of safety and efficacy. Visit 4 (study end): Conducted on day 29 (± 3 days), concluding the trial with final assessments of trial outcomes, including physical examinations and blood sampling. [file Presentation1.pptx]

## Slide 1
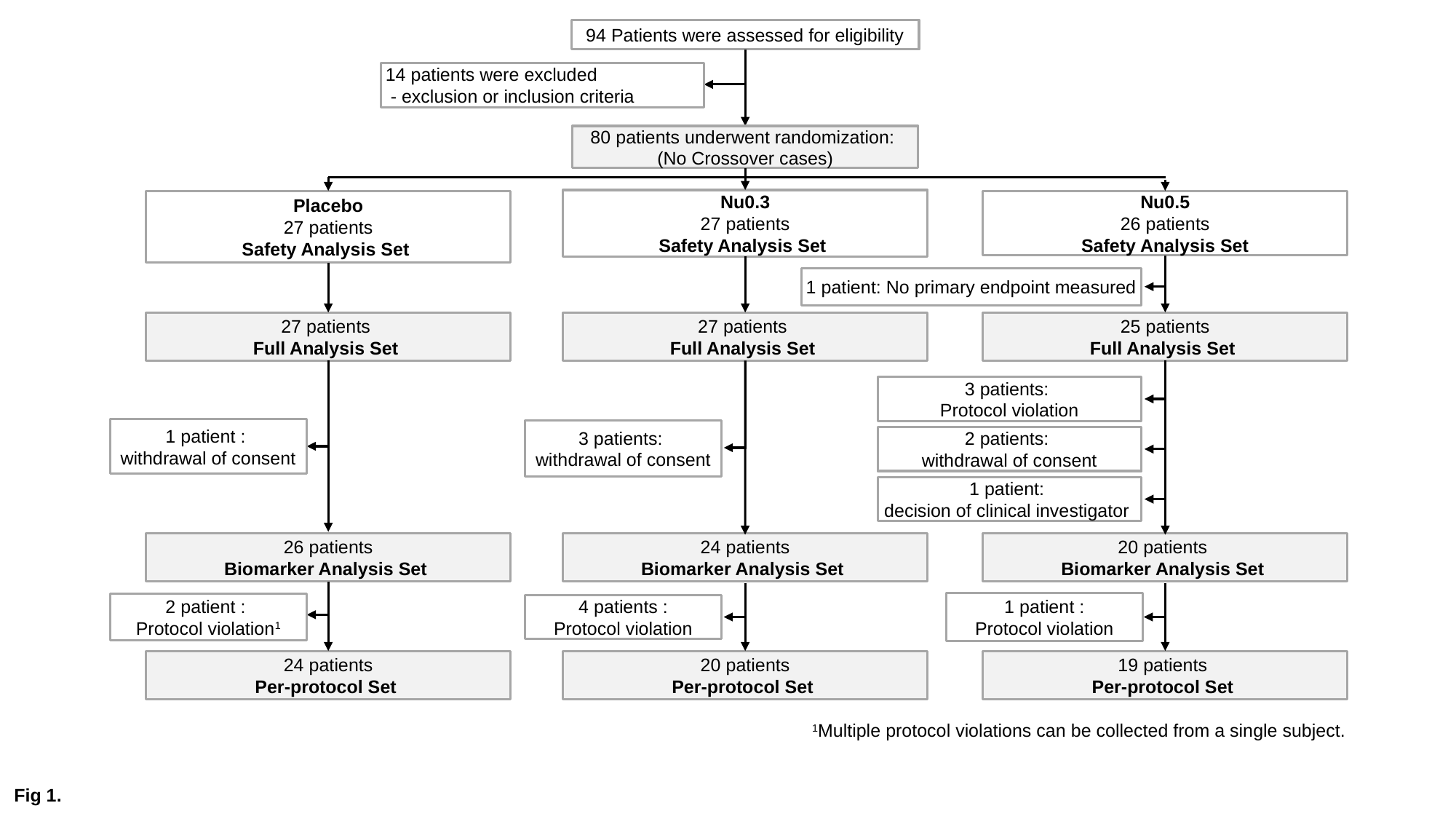

94 Patients were assessed for eligibility
14 patients were excluded
 - exclusion or inclusion criteria
80 patients underwent randomization:
(No Crossover cases)
Nu0.3
27 patients
Safety Analysis Set
Placebo
27 patients
Safety Analysis Set
Nu0.5
26 patients
Safety Analysis Set
1 patient: No primary endpoint measured
27 patients
Full Analysis Set
27 patients
Full Analysis Set
25 patients
Full Analysis Set
3 patients:
Protocol violation
1 patient :
withdrawal of consent
3 patients:
withdrawal of consent
2 patients:
withdrawal of consent
1 patient:
decision of clinical investigator
26 patients
Biomarker Analysis Set
24 patients
Biomarker Analysis Set
20 patients
Biomarker Analysis Set
 1 patient :
Protocol violation
2 patient :
Protocol violation1
 4 patients :
Protocol violation
24 patients
Per-protocol Set
20 patients
Per-protocol Set
19 patients
Per-protocol Set
1Multiple protocol violations can be collected from a single subject.
Fig 1.

## Slide 2
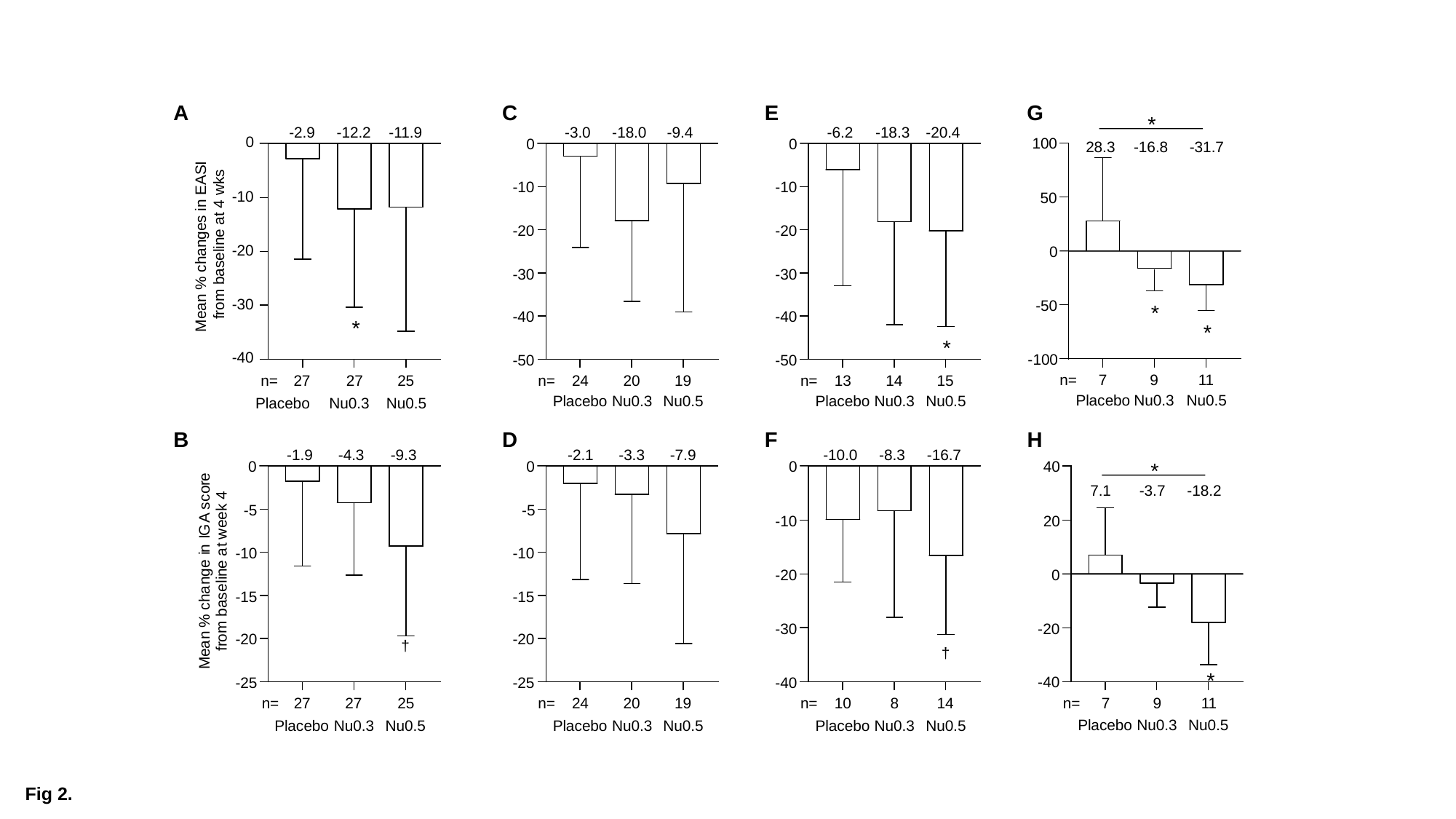

A
C
E
G
*
-2.9
-12.2
-11.9
-3.0
-18.0
-9.4
-6.2
-18.3
-20.4
0
100
0
0
28.3
-16.8
-31.7
-10
-10
-10
50
-20
-20
Mean % changes in EASI
 from baseline at 4 wks
-20
0
-30
-30
-30
-50
*
-40
-40
*
*
*
-40
-100
-50
-50
n=
7
9
11
n=
27
27
25
n=
24
20
19
n=
13
14
15
Placebo
Nu0.3
Nu0.5
Placebo
Nu0.3
Nu0.5
Placebo
Nu0.3
Nu0.5
Nu0.3
Nu0.5
Placebo
B
D
F
H
-1.9
-4.3
-9.3
-2.1
-3.3
-7.9
-10.0
-8.3
-16.7
40
0
0
0
*
e
r
o
7.1
-3.7
-18.2
4
c
s
k
-5
-5
e
A
20
-10
e
G
w
I
t
n
a
-10
-10
i
e
e
g
n
0
-20
i
n
l
e
a
s
-15
-15
h
a
c
b
%
m
-20
-30
n
o
-20
-20
r
a
†
f
†
e
M
-40
-25
-25
-40
n=
7
9
11
n=
27
27
25
n=
24
20
19
n=
10
8
14
Placebo
Nu0.3
Nu0.5
Placebo
Nu0.3
Nu0.5
Placebo
Nu0.3
Nu0.5
Placebo
Nu0.3
Nu0.5
*
Fig 2.

## Slide 3
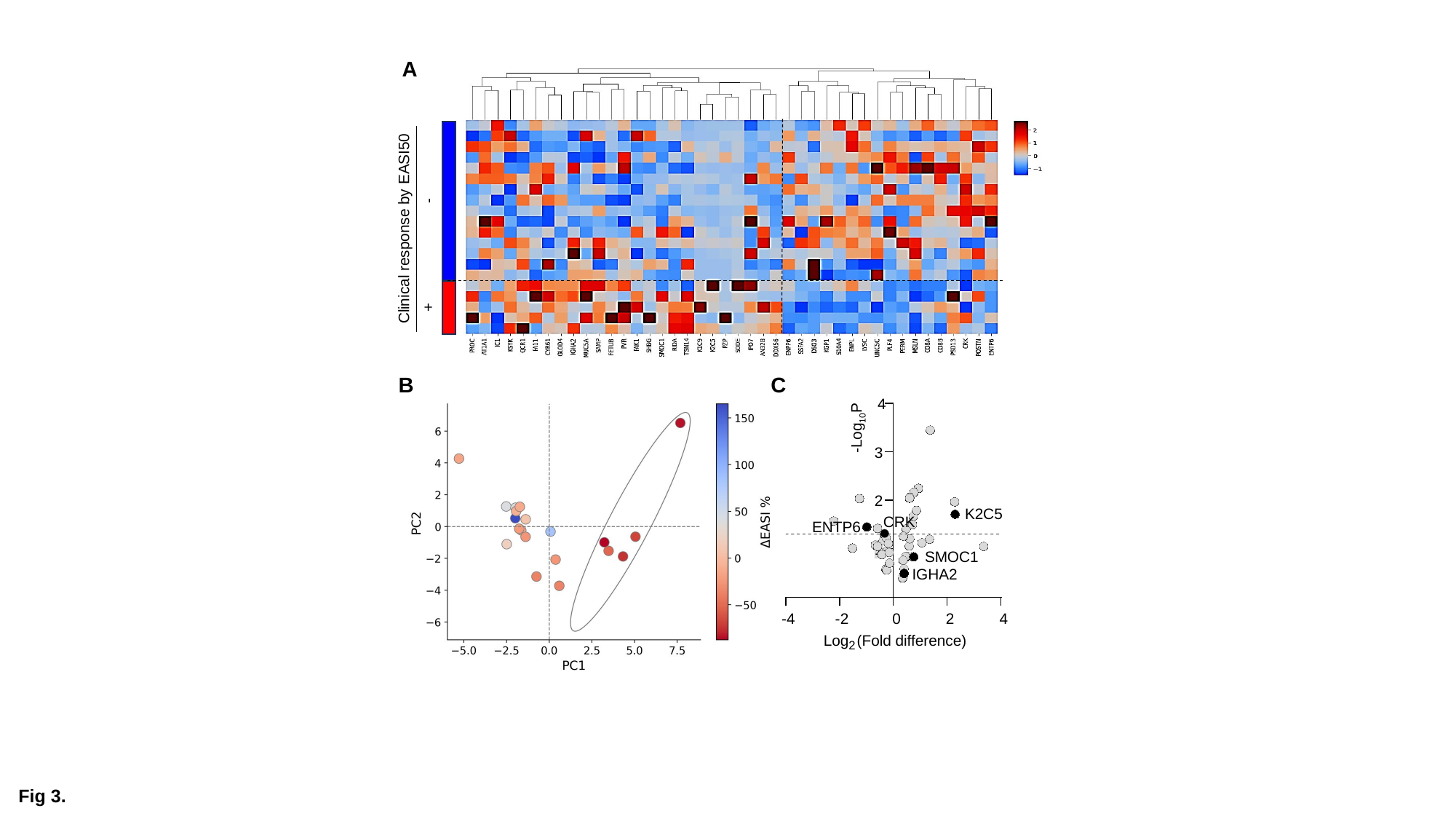

A
2
1
0
-1
-
Clinical response by EASI50
+
B
C
4
-Log10P
3
2
K2C5
CRK
ENTP6
1
SMOC1
IGHA2
-4
-2
0
2
4
Log
(Fold difference)
2
Fig 3.

## Slide 4
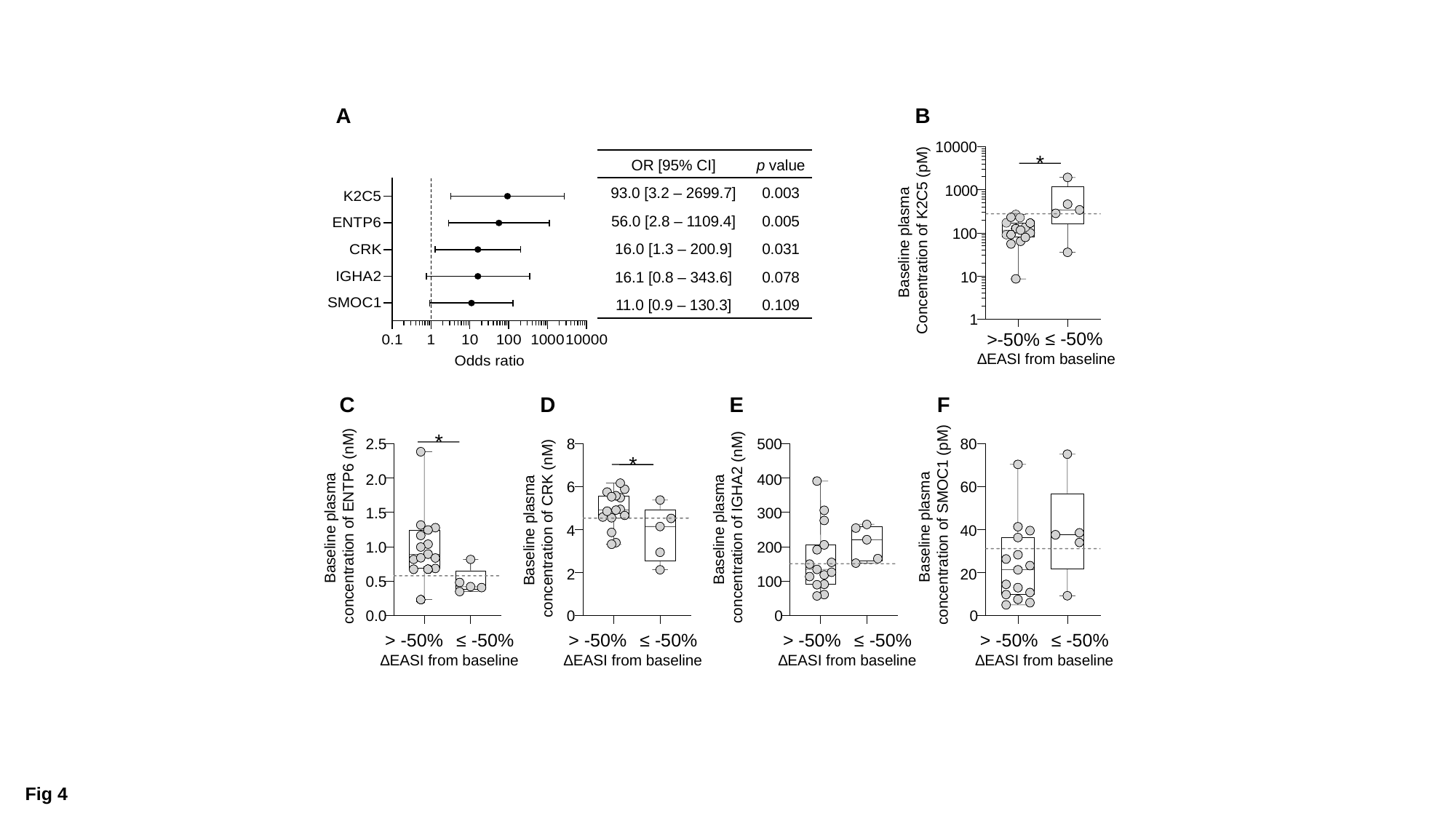

A
B
10000
*
1000
Baseline plasma
Concentration of K2C5 (pM)
100
10
1
≤ -50%
>-50%
∆EASI from baseline
| OR [95% CI] | p value |
| --- | --- |
| 93.0 [3.2 – 2699.7] | 0.003 |
| 56.0 [2.8 – 1109.4] | 0.005 |
| 16.0 [1.3 – 200.9] | 0.031 |
| 16.1 [0.8 – 343.6] | 0.078 |
| 11.0 [0.9 – 130.3] | 0.109 |
C
D
E
F
*
2.5
500
8
80
*
2.0
400
6
60
Baseline plasma
concentration of ENTP6 (nM)
Baseline plasma
concentration of IGHA2 (nM)
1.5
300
Baseline plasma
concentration of CRK (nM)
4
40
1.0
200
2
20
0.5
100
0.0
0
0
0
Baseline plasma
concentration of SMOC1 (pM)
> -50%
≤ -50%
> -50%
≤ -50%
> -50%
≤ -50%
> -50%
≤ -50%
∆EASI from baseline
∆EASI from baseline
∆EASI from baseline
∆EASI from baseline
Fig 4
Fig 3. Differentially expressed proteins between responders and non-responders

## Slide 5
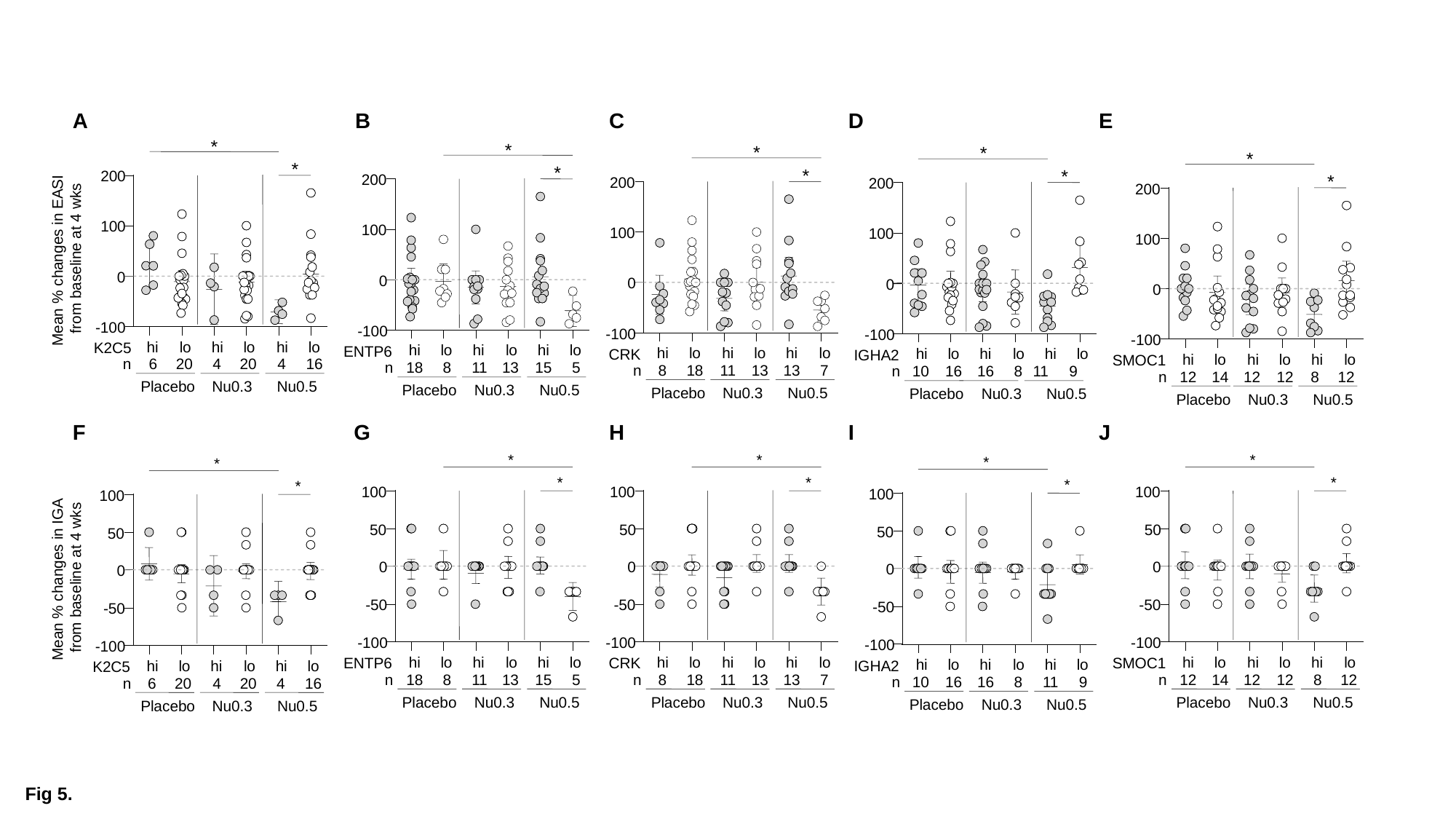

A
B
C
D
E
*
*
200
100
Mean % changes in EASI
 from baseline at 4 wks
0
-100
hi
lo
hi
lo
hi
lo
K2C5
n
6
20
4
20
4
16
Placebo
Nu0.3
Nu0.5
*
*
200
100
0
-100
hi
lo
hi
lo
hi
lo
ENTP6
n
18
8
11
13
15
5
Placebo
Nu0.3
Nu0.5
*
*
200
100
0
-100
hi
lo
hi
lo
hi
lo
CRK
n
8
18
11
13
13
7
Placebo
Nu0.3
Nu0.5
*
*
200
100
0
-100
hi
lo
hi
lo
hi
lo
IGHA2
n
10
16
16
8
Placebo
Nu0.3
Nu0.5
*
*
200
100
0
-100
hi
lo
hi
lo
hi
lo
SMOC1
n
12
14
12
12
Placebo
Nu0.3
Nu0.5
11
9
8
12
F
G
H
I
J
*
*
100
50
0
-50
-100
hi
lo
hi
lo
hi
lo
ENTP6
n
18
8
11
13
15
5
Placebo
Nu0.3
Nu0.5
*
*
100
50
0
-50
-100
hi
lo
hi
lo
hi
lo
CRK
n
8
18
11
13
13
7
Placebo
Nu0.3
Nu0.5
*
*
100
50
0
-50
-100
hi
lo
hi
lo
hi
lo
SMOC1
n
12
14
12
12
8
12
Placebo
Nu0.3
Nu0.5
*
*
100
50
0
-50
-100
hi
lo
hi
lo
hi
lo
IGHA2
n
10
16
16
8
11
9
Placebo
Nu0.3
Nu0.5
*
*
100
50
Mean % changes in IGA
 from baseline at 4 wks
0
-50
-100
hi
lo
hi
lo
hi
lo
K2C5
n
6
20
4
20
4
16
Placebo
Nu0.3
Nu0.5
Fig 5.

## Slide 6
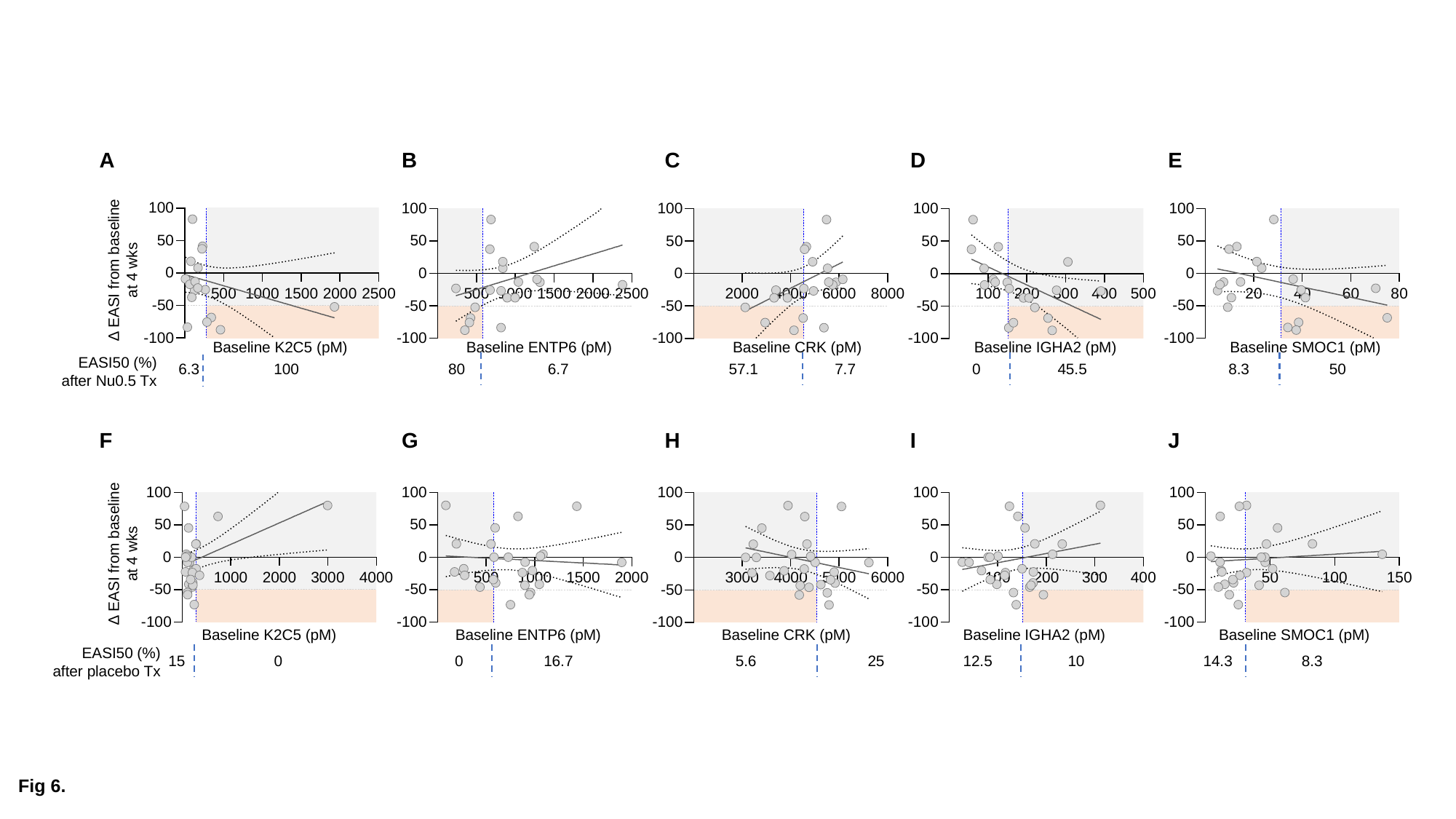

A
B
C
D
E
∆ EASI from baseline
at 4 wks
Baseline K2C5 (pM)
Baseline ENTP6 (pM)
Baseline CRK (pM)
Baseline IGHA2 (pM)
Baseline SMOC1 (pM)
EASI50 (%)
after Nu0.5 Tx
6.3
100
80
6.7
57.1
7.7
0
45.5
8.3
50
F
G
H
I
J
∆ EASI from baseline
at 4 wks
Baseline K2C5 (pM)
Baseline ENTP6 (pM)
Baseline CRK (pM)
Baseline IGHA2 (pM)
Baseline SMOC1 (pM)
EASI50 (%)
after placebo Tx
15
0
0
16.7
5.6
25
12.5
10
14.3
8.3
Fig 6.

## Slide 7
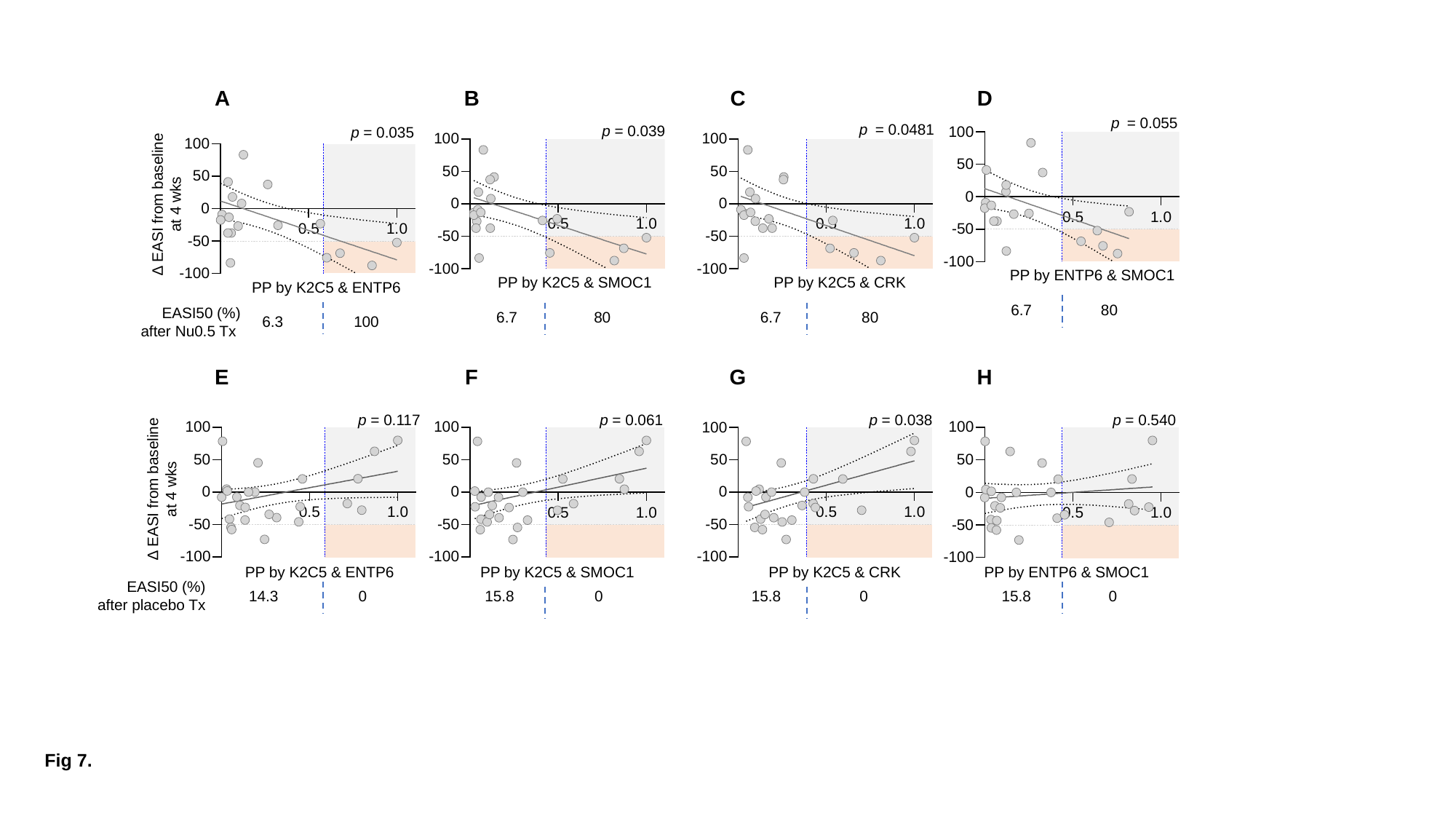

A
B
C
D
 p = 0.055
 p = 0.0481
 p = 0.039
 p = 0.035
∆ EASI from baseline
at 4 wks
PP by ENTP6 & SMOC1
PP by K2C5 & SMOC1
PP by K2C5 & CRK
PP by K2C5 & ENTP6
6.7
80
 EASI50 (%)
after Nu0.5 Tx
6.7
80
6.7
80
6.3
100
E
F
G
H
∆ EASI from baseline
at 4 wks
PP by K2C5 & ENTP6
EASI50 (%)
after placebo Tx
14.3
0
p = 0.540
PP by ENTP6 & SMOC1
15.8
0
p = 0.061
PP by K2C5 & SMOC1
15.8
0
p = 0.038
PP by K2C5 & CRK
15.8
0
p = 0.117
Fig 7.

## Slide 8
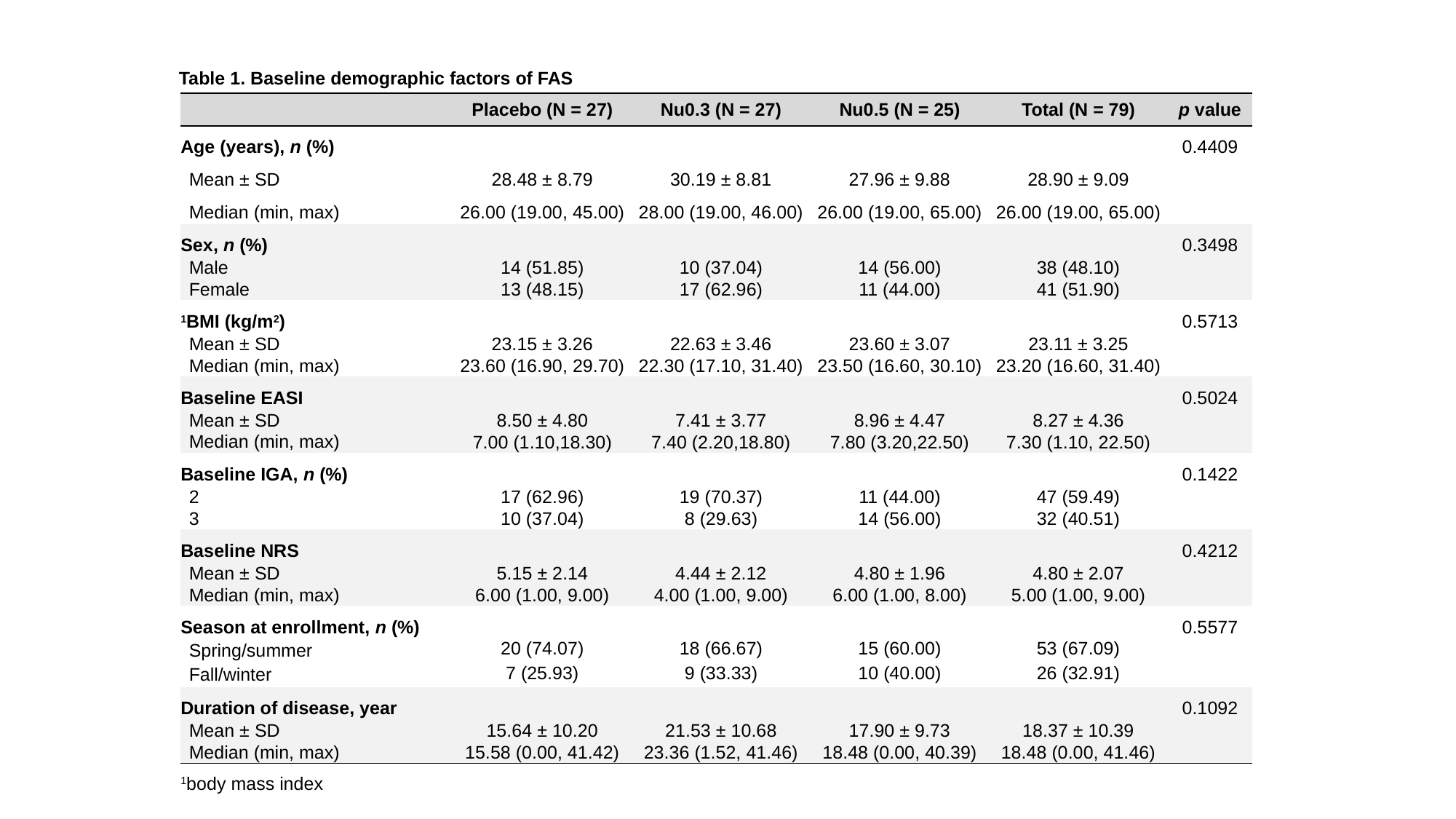

Table 1. Baseline demographic factors of FAS
| | Placebo (N = 27) | Nu0.3 (N = 27) | Nu0.5 (N = 25) | Total (N = 79) | p value |
| --- | --- | --- | --- | --- | --- |
| Age (years), n (%) | | | | | 0.4409 |
| Mean ± SD | 28.48 ± 8.79 | 30.19 ± 8.81 | 27.96 ± 9.88 | 28.90 ± 9.09 | |
| Median (min, max) | 26.00 (19.00, 45.00) | 28.00 (19.00, 46.00) | 26.00 (19.00, 65.00) | 26.00 (19.00, 65.00) | |
| Sex, n (%) | | | | | 0.3498 |
| Male | 14 (51.85) | 10 (37.04) | 14 (56.00) | 38 (48.10) | |
| Female | 13 (48.15) | 17 (62.96) | 11 (44.00) | 41 (51.90) | |
| 1BMI (kg/m2) | | | | | 0.5713 |
| Mean ± SD | 23.15 ± 3.26 | 22.63 ± 3.46 | 23.60 ± 3.07 | 23.11 ± 3.25 | |
| Median (min, max) | 23.60 (16.90, 29.70) | 22.30 (17.10, 31.40) | 23.50 (16.60, 30.10) | 23.20 (16.60, 31.40) | |
| Baseline EASI | | | | | 0.5024 |
| Mean ± SD | 8.50 ± 4.80 | 7.41 ± 3.77 | 8.96 ± 4.47 | 8.27 ± 4.36 | |
| Median (min, max) | 7.00 (1.10,18.30) | 7.40 (2.20,18.80) | 7.80 (3.20,22.50) | 7.30 (1.10, 22.50) | |
| Baseline IGA, n (%) | | | | | 0.1422 |
| 2 | 17 (62.96) | 19 (70.37) | 11 (44.00) | 47 (59.49) | |
| 3 | 10 (37.04) | 8 (29.63) | 14 (56.00) | 32 (40.51) | |
| Baseline NRS | | | | | 0.4212 |
| Mean ± SD | 5.15 ± 2.14 | 4.44 ± 2.12 | 4.80 ± 1.96 | 4.80 ± 2.07 | |
| Median (min, max) | 6.00 (1.00, 9.00) | 4.00 (1.00, 9.00) | 6.00 (1.00, 8.00) | 5.00 (1.00, 9.00) | |
| Season at enrollment, n (%) | | | | | 0.5577 |
| Spring/summer | 20 (74.07) | 18 (66.67) | 15 (60.00) | 53 (67.09) | |
| Fall/winter | 7 (25.93) | 9 (33.33) | 10 (40.00) | 26 (32.91) | |
| Duration of disease, year | | | | | 0.1092 |
| Mean ± SD | 15.64 ± 10.20 | 21.53 ± 10.68 | 17.90 ± 9.73 | 18.37 ± 10.39 | |
| Median (min, max) | 15.58 (0.00, 41.42) | 23.36 (1.52, 41.46) | 18.48 (0.00, 40.39) | 18.48 (0.00, 41.46) | |
| 1body mass index | | | | | |

## Slide 9
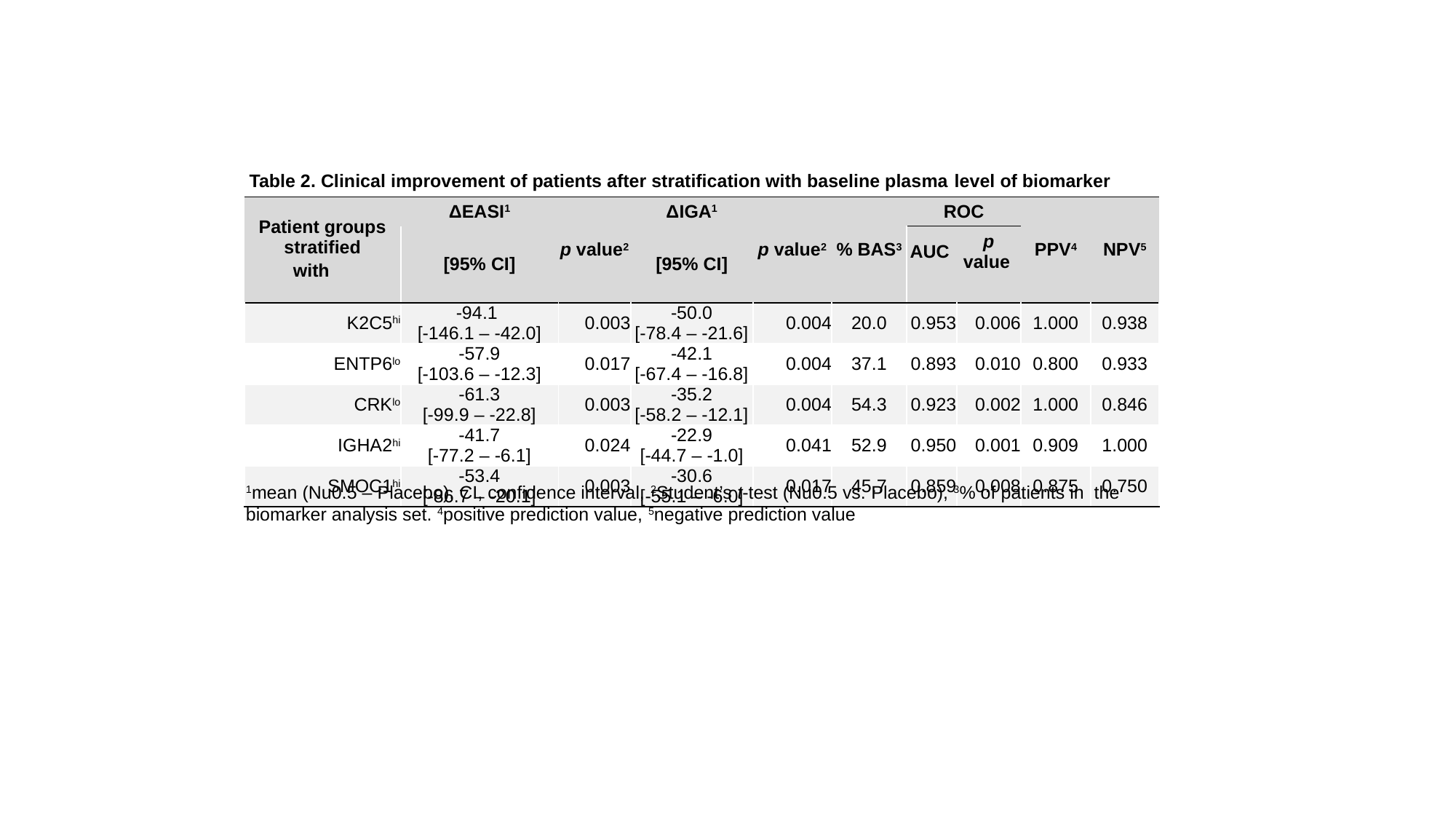

Table 2. Clinical improvement of patients after stratification with baseline plasma level of biomarker
| Patient groups stratified with | ΔEASI1 | p value2 | ΔIGA1 | p value2 | % BAS3 | ROC | | PPV4 | NPV5 |
| --- | --- | --- | --- | --- | --- | --- | --- | --- | --- |
| | [95% CI] | | [95% CI] | | | AUC | p value | | |
| K2C5hi | -94.1 [-146.1 – -42.0] | 0.003 | -50.0 [-78.4 – -21.6] | 0.004 | 20.0 | 0.953 | 0.006 | 1.000 | 0.938 |
| ENTP6lo | -57.9 [-103.6 – -12.3] | 0.017 | -42.1 [-67.4 – -16.8] | 0.004 | 37.1 | 0.893 | 0.010 | 0.800 | 0.933 |
| CRKlo | -61.3 [-99.9 – -22.8] | 0.003 | -35.2 [-58.2 – -12.1] | 0.004 | 54.3 | 0.923 | 0.002 | 1.000 | 0.846 |
| IGHA2hi | -41.7 [-77.2 – -6.1] | 0.024 | -22.9 [-44.7 – -1.0] | 0.041 | 52.9 | 0.950 | 0.001 | 0.909 | 1.000 |
| SMOC1hi | -53.4 [-86.7 – -20.1] | 0.003 | -30.6 [-55.1 – -6.0] | 0.017 | 45.7 | 0.859 | 0.008 | 0.875 | 0.750 |
1mean (Nu0.5 – Placebo), CI, confidence interval, 2Student’s t-test (Nu0.5 vs. Placebo), 3% of patients in the biomarker analysis set. 4positive prediction value, 5negative prediction value

## Slide 10
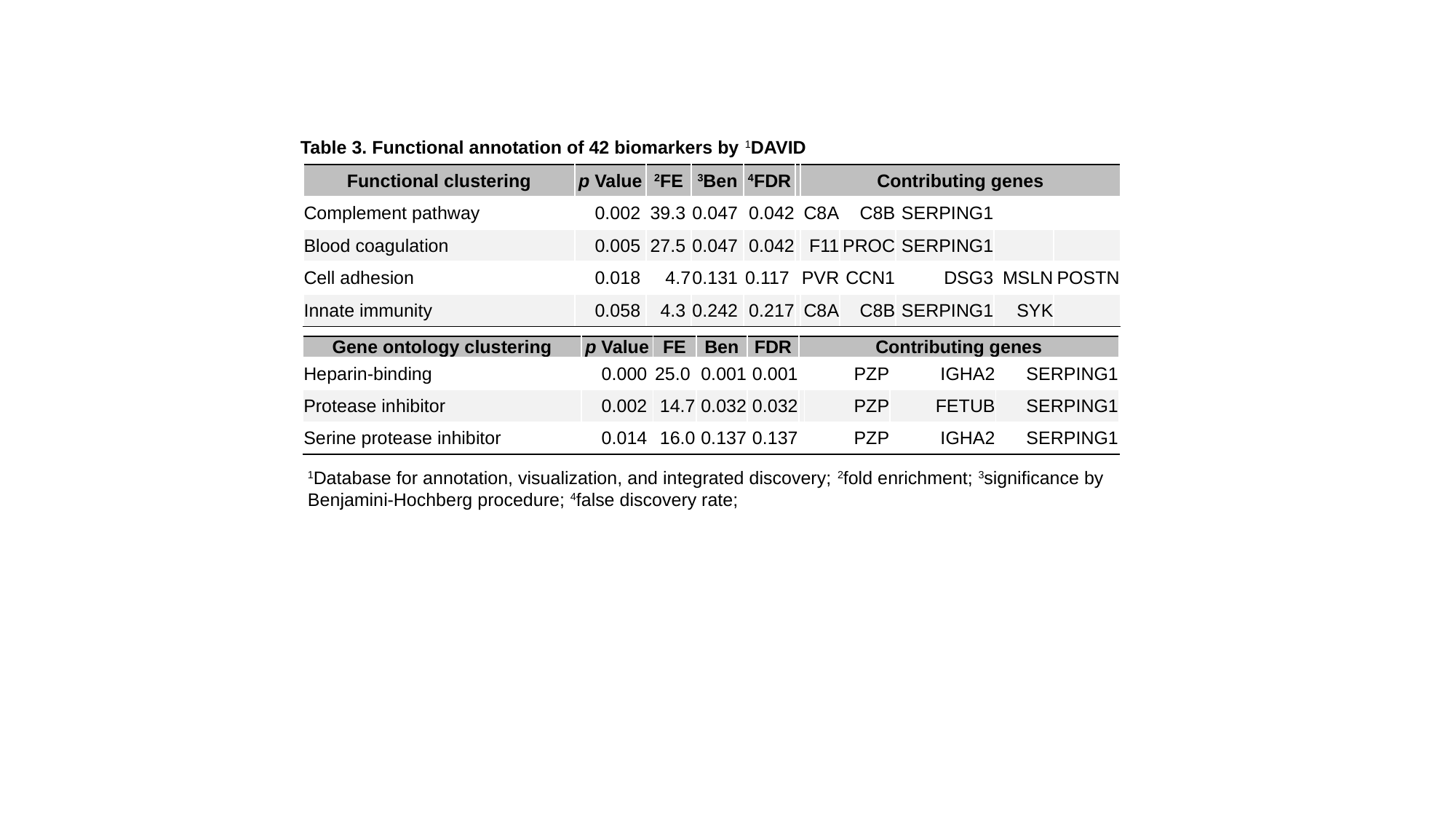

Table 3. Functional annotation of 42 biomarkers by 1DAVID
| Functional clustering | p Value | 2FE | 3Ben | 4FDR | | Contributing genes | | | | |
| --- | --- | --- | --- | --- | --- | --- | --- | --- | --- | --- |
| Complement pathway | 0.002 | 39.3 | 0.047 | 0.042 | | C8A | C8B | SERPING1 | | |
| Blood coagulation | 0.005 | 27.5 | 0.047 | 0.042 | | F11 | PROC | SERPING1 | | |
| Cell adhesion | 0.018 | 4.7 | 0.131 | 0.117 | | PVR | CCN1 | DSG3 | MSLN | POSTN |
| Innate immunity | 0.058 | 4.3 | 0.242 | 0.217 | | C8A | C8B | SERPING1 | SYK | |
| Gene ontology clustering | p Value | FE | Ben | FDR | Contributing genes | | | |
| --- | --- | --- | --- | --- | --- | --- | --- | --- |
| Heparin-binding | 0.000 | 25.0 | 0.001 | 0.001 | | PZP | IGHA2 | SERPING1 |
| Protease inhibitor | 0.002 | 14.7 | 0.032 | 0.032 | | PZP | FETUB | SERPING1 |
| Serine protease inhibitor | 0.014 | 16.0 | 0.137 | 0.137 | | PZP | IGHA2 | SERPING1 |
1Database for annotation, visualization, and integrated discovery; 2fold enrichment; 3significance by Benjamini-Hochberg procedure; 4false discovery rate;

## Slide 11
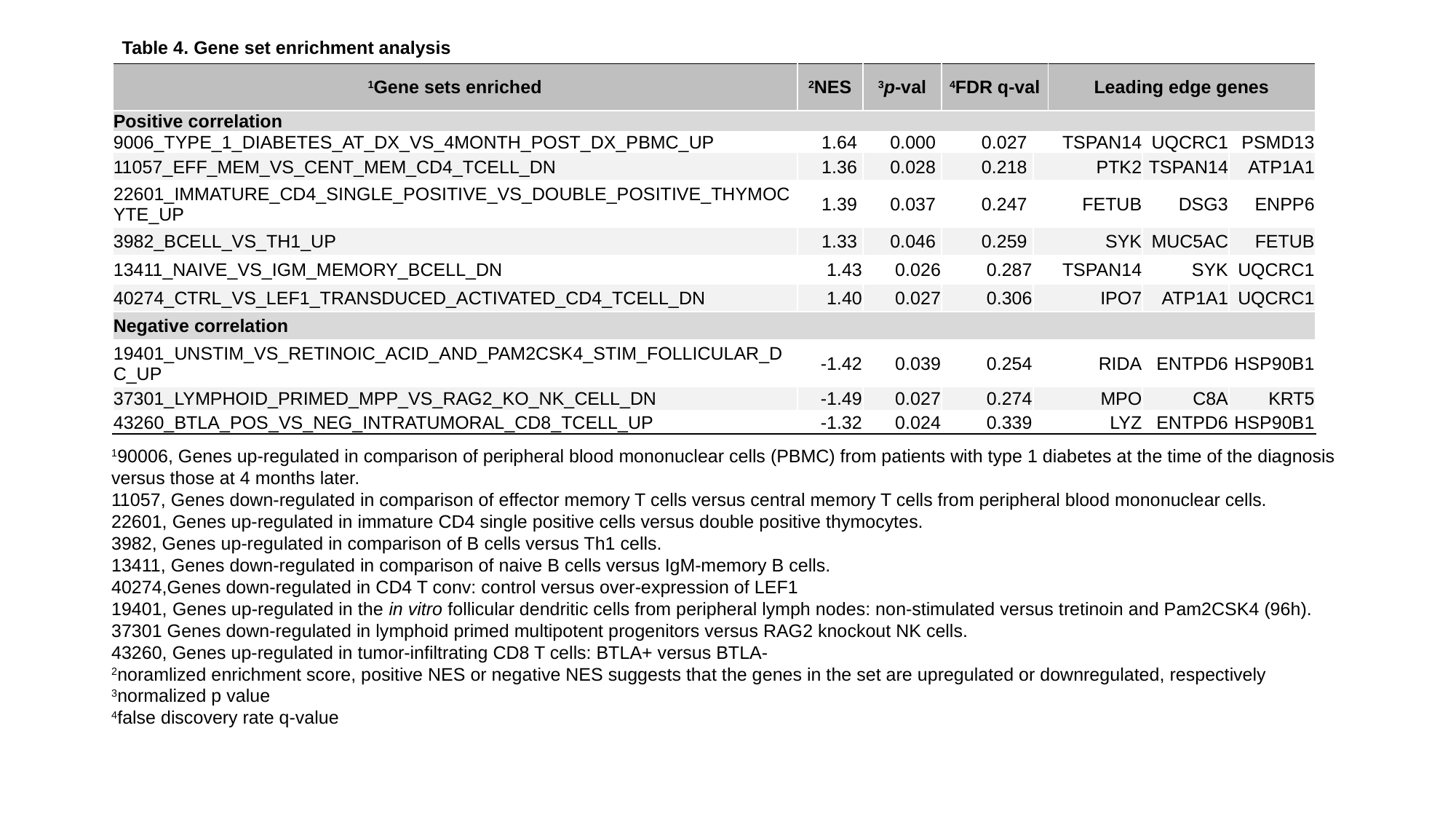

Table 4. Gene set enrichment analysis
| 1Gene sets enriched | 2NES | 3p-val | 4FDR q-val | Leading edge genes | Leading edge genes | | |
| --- | --- | --- | --- | --- | --- | --- | --- |
| Positive correlation | | | | | | | |
| 9006\_TYPE\_1\_DIABETES\_AT\_DX\_VS\_4MONTH\_POST\_DX\_PBMC\_UP | 1.64 | 0.000 | 0.027 | TSPAN14 | | UQCRC1 | PSMD13 |
| 11057\_EFF\_MEM\_VS\_CENT\_MEM\_CD4\_TCELL\_DN | 1.36 | 0.028 | 0.218 | PTK2 | | TSPAN14 | ATP1A1 |
| 22601\_IMMATURE\_CD4\_SINGLE\_POSITIVE\_VS\_DOUBLE\_POSITIVE\_THYMOCYTE\_UP | 1.39 | 0.037 | 0.247 | FETUB | | DSG3 | ENPP6 |
| 3982\_BCELL\_VS\_TH1\_UP | 1.33 | 0.046 | 0.259 | SYK | | MUC5AC | FETUB |
| 13411\_NAIVE\_VS\_IGM\_MEMORY\_BCELL\_DN | 1.43 | 0.026 | 0.287 | TSPAN14 | | SYK | UQCRC1 |
| 40274\_CTRL\_VS\_LEF1\_TRANSDUCED\_ACTIVATED\_CD4\_TCELL\_DN | 1.40 | 0.027 | 0.306 | IPO7 | | ATP1A1 | UQCRC1 |
| Negative correlation | | | | | | | |
| 19401\_UNSTIM\_VS\_RETINOIC\_ACID\_AND\_PAM2CSK4\_STIM\_FOLLICULAR\_DC\_UP | -1.42 | 0.039 | 0.254 | RIDA | | ENTPD6 | HSP90B1 |
| 37301\_LYMPHOID\_PRIMED\_MPP\_VS\_RAG2\_KO\_NK\_CELL\_DN | -1.49 | 0.027 | 0.274 | MPO | | C8A | KRT5 |
| 43260\_BTLA\_POS\_VS\_NEG\_INTRATUMORAL\_CD8\_TCELL\_UP | -1.32 | 0.024 | 0.339 | LYZ | | ENTPD6 | HSP90B1 |
190006, Genes up-regulated in comparison of peripheral blood mononuclear cells (PBMC) from patients with type 1 diabetes at the time of the diagnosis versus those at 4 months later.
11057, Genes down-regulated in comparison of effector memory T cells versus central memory T cells from peripheral blood mononuclear cells.
22601, Genes up-regulated in immature CD4 single positive cells versus double positive thymocytes.
3982, Genes up-regulated in comparison of B cells versus Th1 cells.
13411, Genes down-regulated in comparison of naive B cells versus IgM-memory B cells.
40274,Genes down-regulated in CD4 T conv: control versus over-expression of LEF1
19401, Genes up-regulated in the in vitro follicular dendritic cells from peripheral lymph nodes: non-stimulated versus tretinoin and Pam2CSK4 (96h).
37301 Genes down-regulated in lymphoid primed multipotent progenitors versus RAG2 knockout NK cells.
43260, Genes up-regulated in tumor-infiltrating CD8 T cells: BTLA+ versus BTLA-
2noramlized enrichment score, positive NES or negative NES suggests that the genes in the set are upregulated or downregulated, respectively
3normalized p value
4false discovery rate q-value

## Slide 12
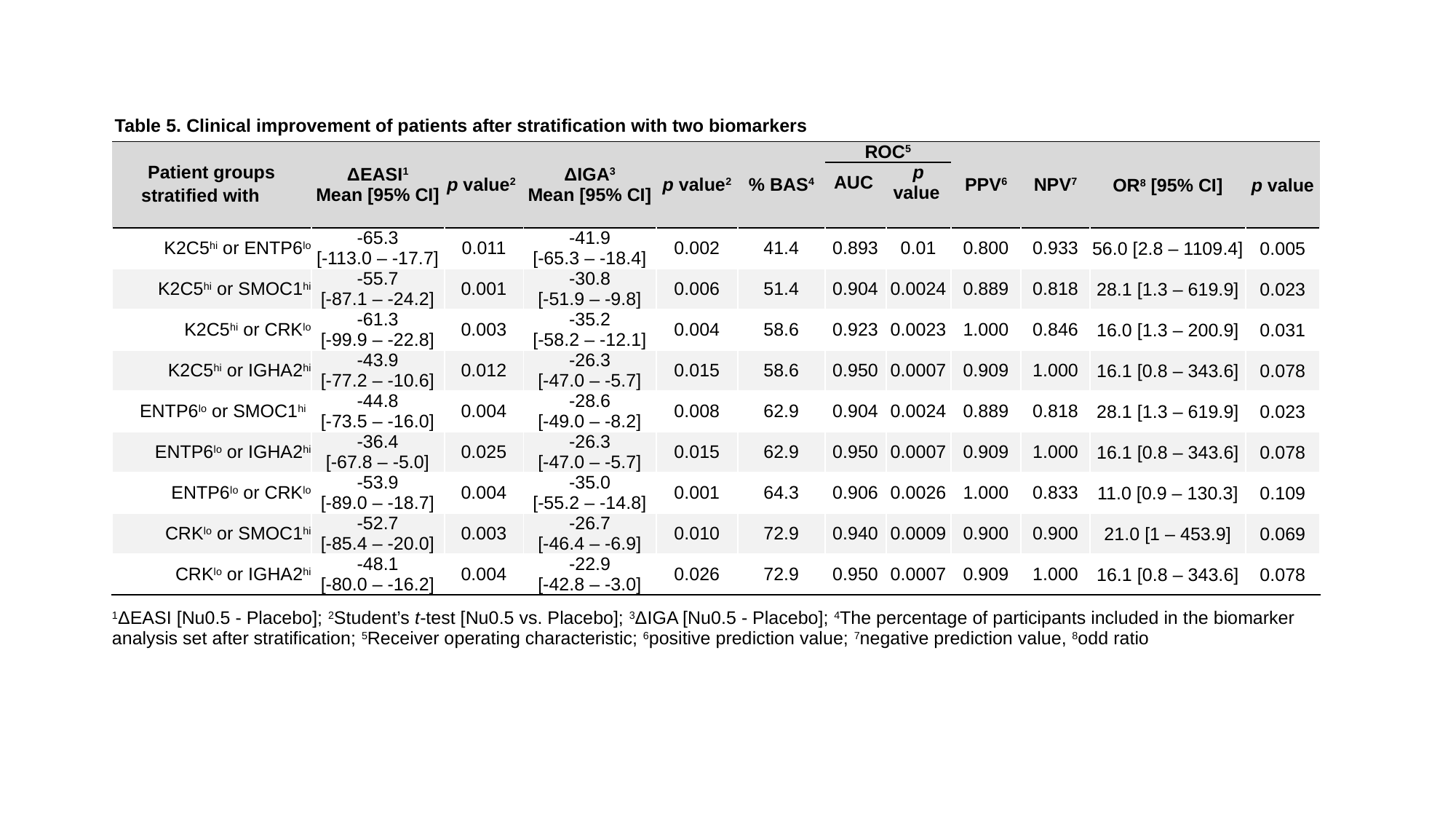

Table 5. Clinical improvement of patients after stratification with two biomarkers
| Patient groups stratified with | ΔEASI1 Mean [95% CI] | p value2 | ΔIGA3 Mean [95% CI] | p value2 | % BAS4 | ROC5 | | PPV6 | NPV7 | OR8 [95% CI] | p value |
| --- | --- | --- | --- | --- | --- | --- | --- | --- | --- | --- | --- |
| | | | | | | AUC | p value | | | | |
| K2C5hi or ENTP6lo | -65.3 [-113.0 – -17.7] | 0.011 | -41.9 [-65.3 – -18.4] | 0.002 | 41.4 | 0.893 | 0.01 | 0.800 | 0.933 | 56.0 [2.8 – 1109.4] | 0.005 |
| K2C5hi or SMOC1hi | -55.7 [-87.1 – -24.2] | 0.001 | -30.8 [-51.9 – -9.8] | 0.006 | 51.4 | 0.904 | 0.0024 | 0.889 | 0.818 | 28.1 [1.3 – 619.9] | 0.023 |
| K2C5hi or CRKlo | -61.3 [-99.9 – -22.8] | 0.003 | -35.2 [-58.2 – -12.1] | 0.004 | 58.6 | 0.923 | 0.0023 | 1.000 | 0.846 | 16.0 [1.3 – 200.9] | 0.031 |
| K2C5hi or IGHA2hi | -43.9 [-77.2 – -10.6] | 0.012 | -26.3 [-47.0 – -5.7] | 0.015 | 58.6 | 0.950 | 0.0007 | 0.909 | 1.000 | 16.1 [0.8 – 343.6] | 0.078 |
| ENTP6lo or SMOC1hi | -44.8 [-73.5 – -16.0] | 0.004 | -28.6 [-49.0 – -8.2] | 0.008 | 62.9 | 0.904 | 0.0024 | 0.889 | 0.818 | 28.1 [1.3 – 619.9] | 0.023 |
| ENTP6lo or IGHA2hi | -36.4 [-67.8 – -5.0] | 0.025 | -26.3 [-47.0 – -5.7] | 0.015 | 62.9 | 0.950 | 0.0007 | 0.909 | 1.000 | 16.1 [0.8 – 343.6] | 0.078 |
| ENTP6lo or CRKlo | -53.9 [-89.0 – -18.7] | 0.004 | -35.0 [-55.2 – -14.8] | 0.001 | 64.3 | 0.906 | 0.0026 | 1.000 | 0.833 | 11.0 [0.9 – 130.3] | 0.109 |
| CRKlo or SMOC1hi | -52.7 [-85.4 – -20.0] | 0.003 | -26.7 [-46.4 – -6.9] | 0.010 | 72.9 | 0.940 | 0.0009 | 0.900 | 0.900 | 21.0 [1 – 453.9] | 0.069 |
| CRKlo or IGHA2hi | -48.1 [-80.0 – -16.2] | 0.004 | -22.9 [-42.8 – -3.0] | 0.026 | 72.9 | 0.950 | 0.0007 | 0.909 | 1.000 | 16.1 [0.8 – 343.6] | 0.078 |
| 1ΔEASI [Nu0.5 - Placebo]; 2Student’s t-test [Nu0.5 vs. Placebo]; 3ΔIGA [Nu0.5 - Placebo]; 4The percentage of participants included in the biomarker analysis set after stratification; 5Receiver operating characteristic; 6positive prediction value; 7negative prediction value, 8odd ratio |
| --- |

## Slide 13
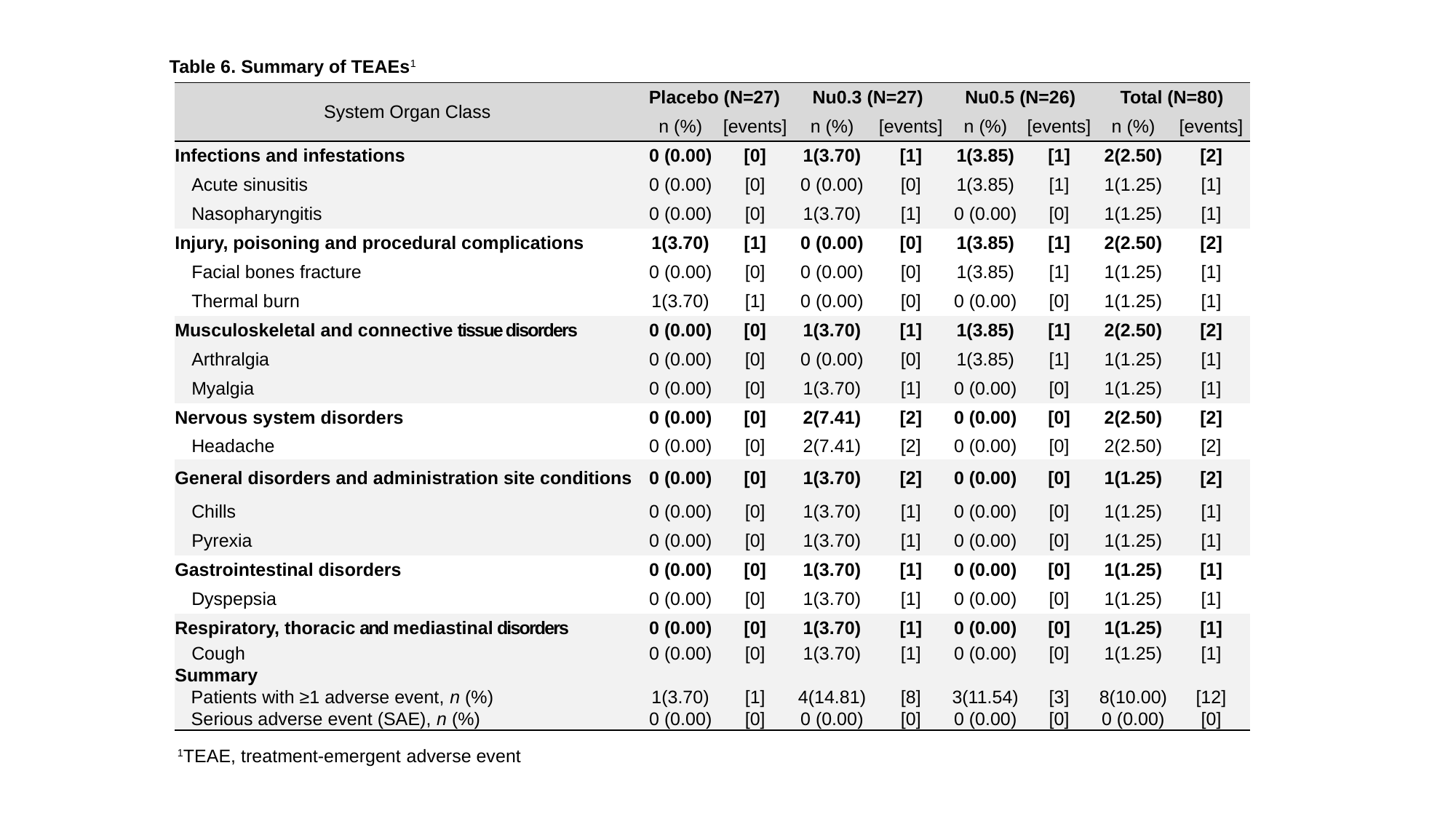

Table 6. Summary of TEAEs1
| System Organ Class | Placebo (N=27) | | Nu0.3 (N=27) | | Nu0.5 (N=26) | | Total (N=80) | |
| --- | --- | --- | --- | --- | --- | --- | --- | --- |
| | n (%) | [events] | n (%) | [events] | n (%) | [events] | n (%) | [events] |
| Infections and infestations | 0 (0.00) | [0] | 1(3.70) | [1] | 1(3.85) | [1] | 2(2.50) | [2] |
| Acute sinusitis | 0 (0.00) | [0] | 0 (0.00) | [0] | 1(3.85) | [1] | 1(1.25) | [1] |
| Nasopharyngitis | 0 (0.00) | [0] | 1(3.70) | [1] | 0 (0.00) | [0] | 1(1.25) | [1] |
| Injury, poisoning and procedural complications | 1(3.70) | [1] | 0 (0.00) | [0] | 1(3.85) | [1] | 2(2.50) | [2] |
| Facial bones fracture | 0 (0.00) | [0] | 0 (0.00) | [0] | 1(3.85) | [1] | 1(1.25) | [1] |
| Thermal burn | 1(3.70) | [1] | 0 (0.00) | [0] | 0 (0.00) | [0] | 1(1.25) | [1] |
| Musculoskeletal and connective tissue disorders | 0 (0.00) | [0] | 1(3.70) | [1] | 1(3.85) | [1] | 2(2.50) | [2] |
| Arthralgia | 0 (0.00) | [0] | 0 (0.00) | [0] | 1(3.85) | [1] | 1(1.25) | [1] |
| Myalgia | 0 (0.00) | [0] | 1(3.70) | [1] | 0 (0.00) | [0] | 1(1.25) | [1] |
| Nervous system disorders | 0 (0.00) | [0] | 2(7.41) | [2] | 0 (0.00) | [0] | 2(2.50) | [2] |
| Headache | 0 (0.00) | [0] | 2(7.41) | [2] | 0 (0.00) | [0] | 2(2.50) | [2] |
| General disorders and administration site conditions | 0 (0.00) | [0] | 1(3.70) | [2] | 0 (0.00) | [0] | 1(1.25) | [2] |
| Chills | 0 (0.00) | [0] | 1(3.70) | [1] | 0 (0.00) | [0] | 1(1.25) | [1] |
| Pyrexia | 0 (0.00) | [0] | 1(3.70) | [1] | 0 (0.00) | [0] | 1(1.25) | [1] |
| Gastrointestinal disorders | 0 (0.00) | [0] | 1(3.70) | [1] | 0 (0.00) | [0] | 1(1.25) | [1] |
| Dyspepsia | 0 (0.00) | [0] | 1(3.70) | [1] | 0 (0.00) | [0] | 1(1.25) | [1] |
| Respiratory, thoracic and mediastinal disorders | 0 (0.00) | [0] | 1(3.70) | [1] | 0 (0.00) | [0] | 1(1.25) | [1] |
| Cough | 0 (0.00) | [0] | 1(3.70) | [1] | 0 (0.00) | [0] | 1(1.25) | [1] |
| Summary | | | | | | | | |
| Patients with ≥1 adverse event, n (%) | 1(3.70) | [1] | 4(14.81) | [8] | 3(11.54) | [3] | 8(10.00) | [12] |
| Serious adverse event (SAE), n (%) | 0 (0.00) | [0] | 0 (0.00) | [0] | 0 (0.00) | [0] | 0 (0.00) | [0] |
1TEAE, treatment-emergent adverse event

## Slide 14
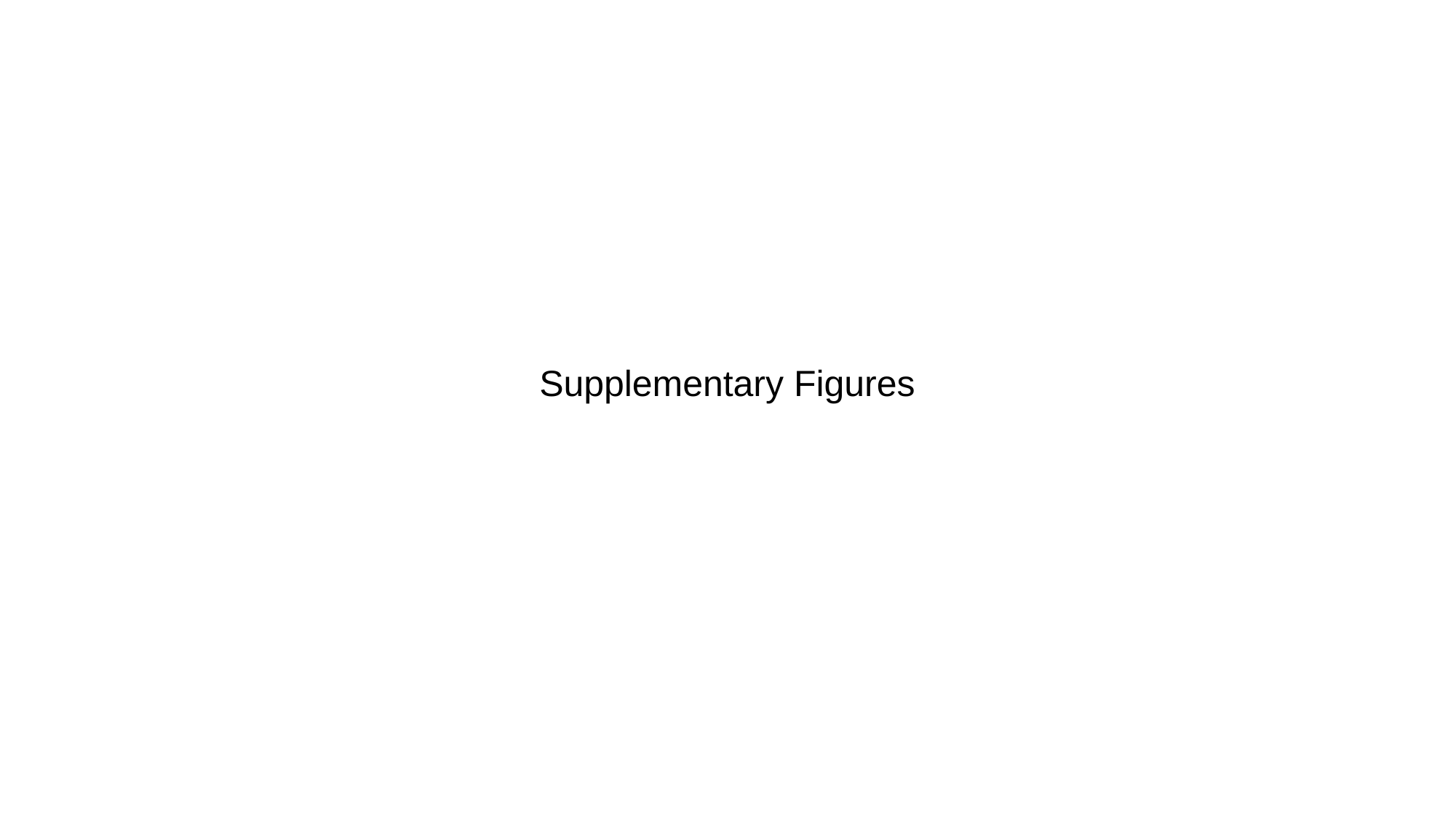

Supplementary Figures

## Slide 15
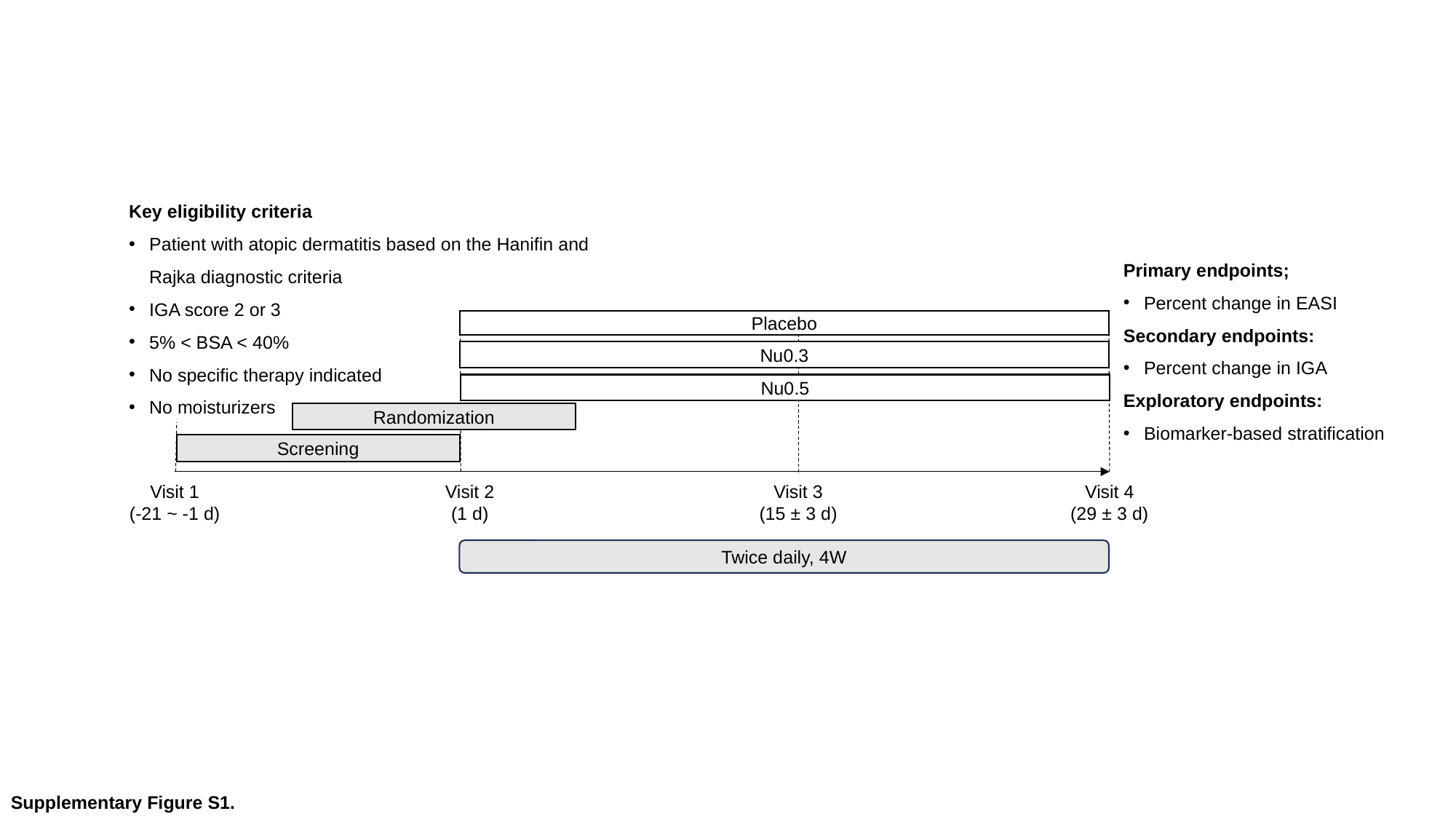

Key eligibility criteria
Patient with atopic dermatitis based on the Hanifin and Rajka diagnostic criteria
IGA score 2 or 3
5% < BSA < 40%
No specific therapy indicated
No moisturizers
Primary endpoints;
Percent change in EASI
Secondary endpoints:
Percent change in IGA
Exploratory endpoints:
Biomarker-based stratification
Placebo
Nu0.3
Nu0.5
Randomization
Screening
Visit 1
(-21 ~ -1 d)
Visit 2
(1 d)
Visit 3
(15 ± 3 d)
Visit 4
(29 ± 3 d)
Twice daily, 4W
Supplementary Figure S1.

## Slide 16
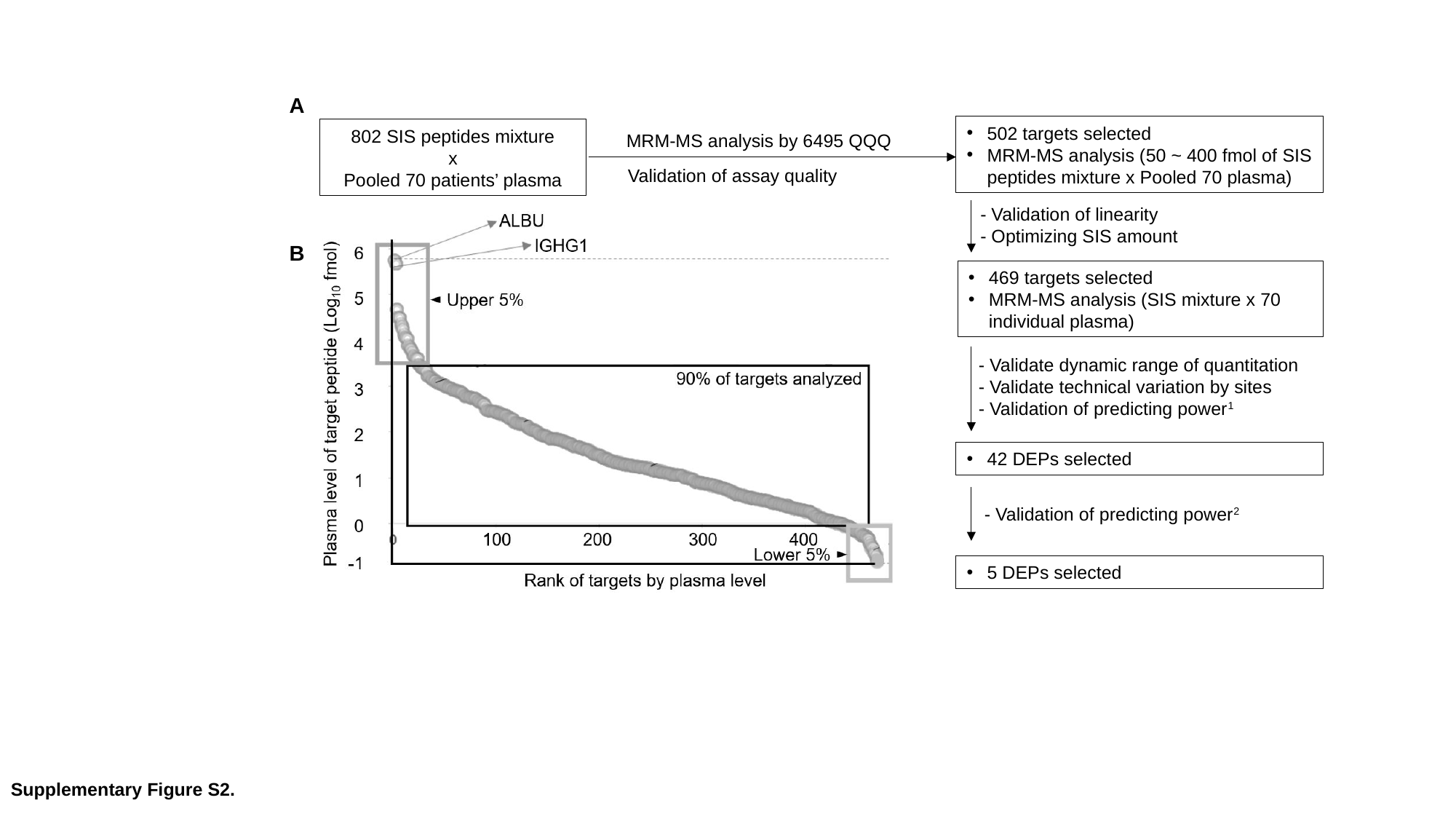

A
502 targets selected
MRM-MS analysis (50 ~ 400 fmol of SIS peptides mixture x Pooled 70 plasma)
802 SIS peptides mixture
x
Pooled 70 patients’ plasma
MRM-MS analysis by 6495 QQQ
Validation of assay quality
- Validation of linearity
- Optimizing SIS amount
B
469 targets selected
MRM-MS analysis (SIS mixture x 70 individual plasma)
- Validate dynamic range of quantitation
- Validate technical variation by sites
- Validation of predicting power1
42 DEPs selected
- Validation of predicting power2
5 DEPs selected
Supplementary Figure S2.

## Slide 17
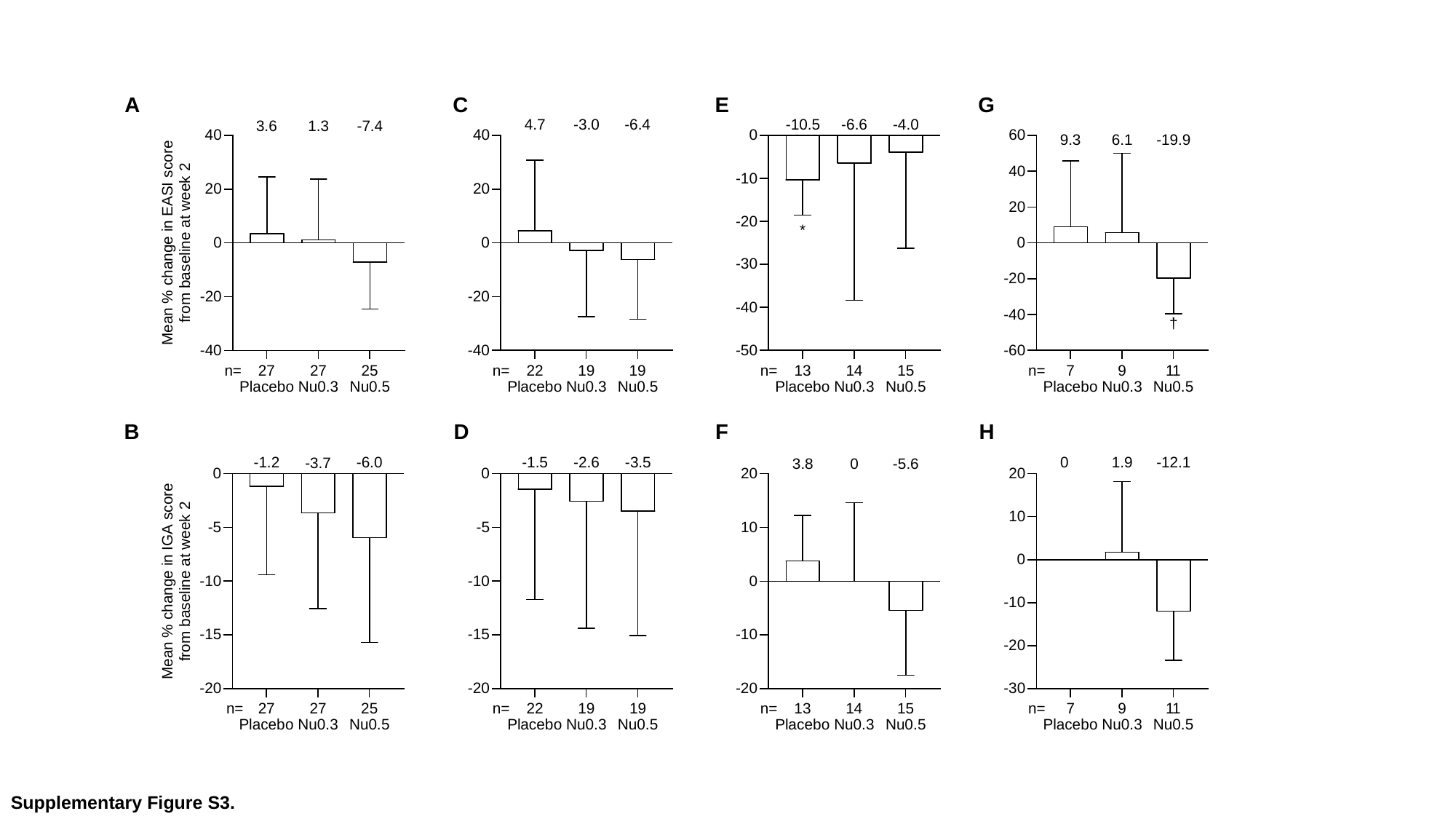

A
C
E
G
B
D
F
H
Supplementary Figure S3.

## Slide 18
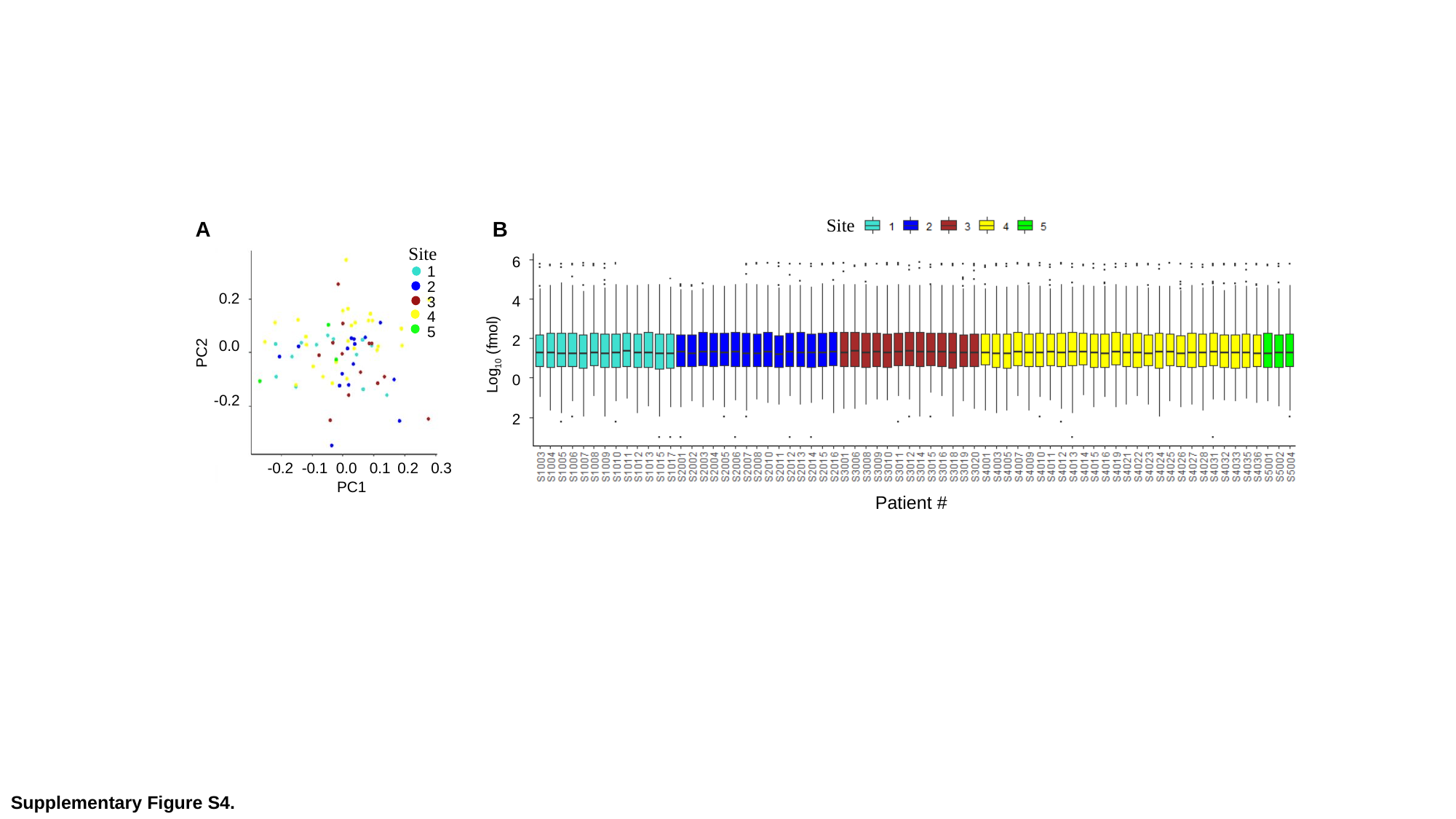

Site
A
B
Site
6
4
2
0
2
1
2
3
4
5
0.2
0.0
-0.2
Log10 (fmol)
PC2
Log10 (fmol)
-0.2 -0.1 0.0 0.1 0.2 0.3
PC1
Patient #
Supplementary Figure S4.

## Slide 19
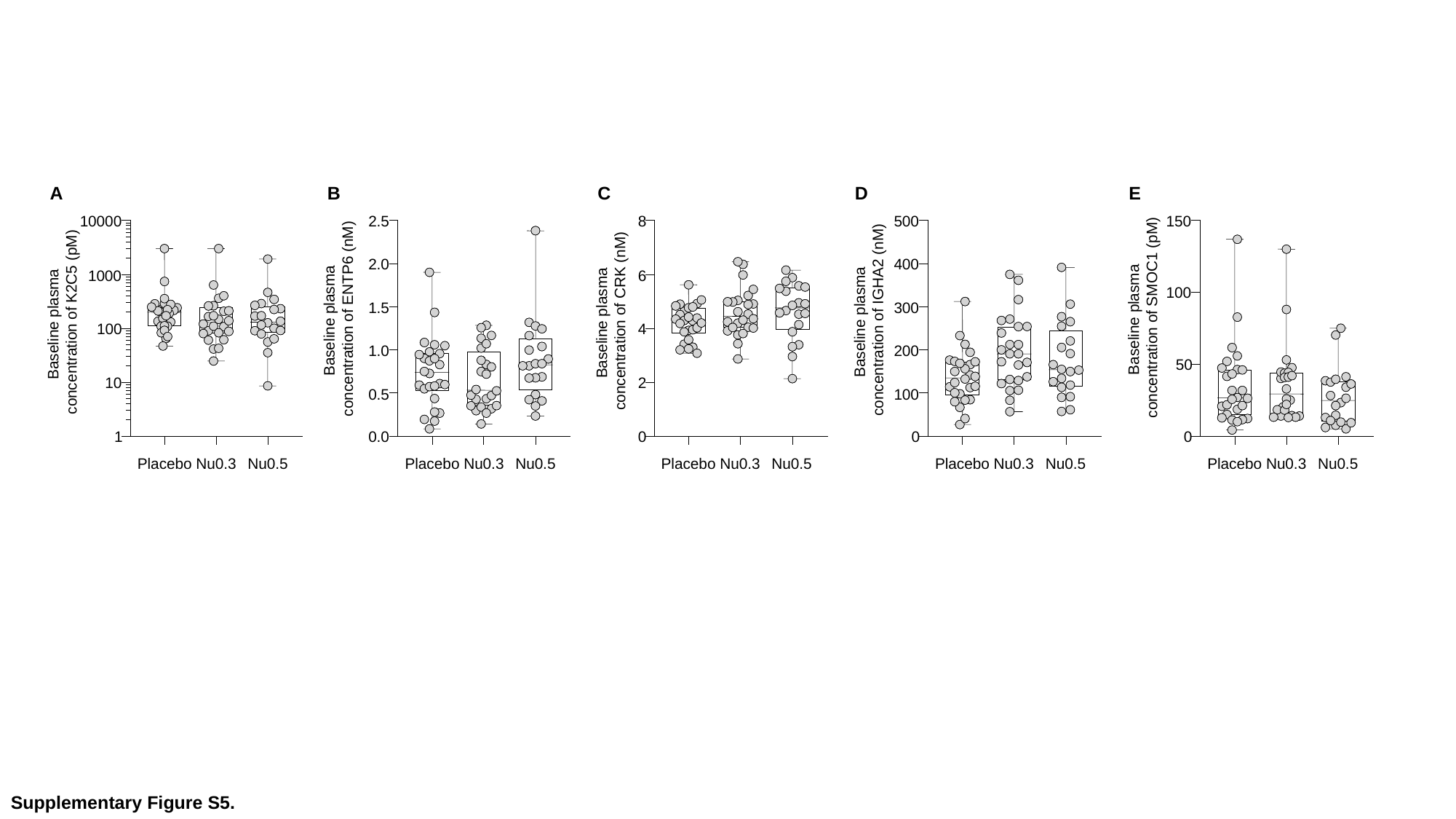

A
B
C
D
E
10000
1000
100
10
1
2.5
2.0
1.5
1.0
0.5
0.0
8
6
4
2
0
500
400
300
200
100
0
150
100
50
0
Baseline plasma
concentration of SMOC1 (pM)
Baseline plasma
concentration of ENTP6 (nM)
Baseline plasma
concentration of IGHA2 (nM)
Baseline plasma
concentration of CRK (nM)
Baseline plasma
concentration of K2C5 (pM)
Placebo
Nu0.3
Nu0.5
Placebo
Nu0.3
Nu0.5
Placebo
Nu0.3
Nu0.5
Placebo
Nu0.3
Nu0.5
Placebo
Nu0.3
Nu0.5
Supplementary Figure S5.

## Slide 20
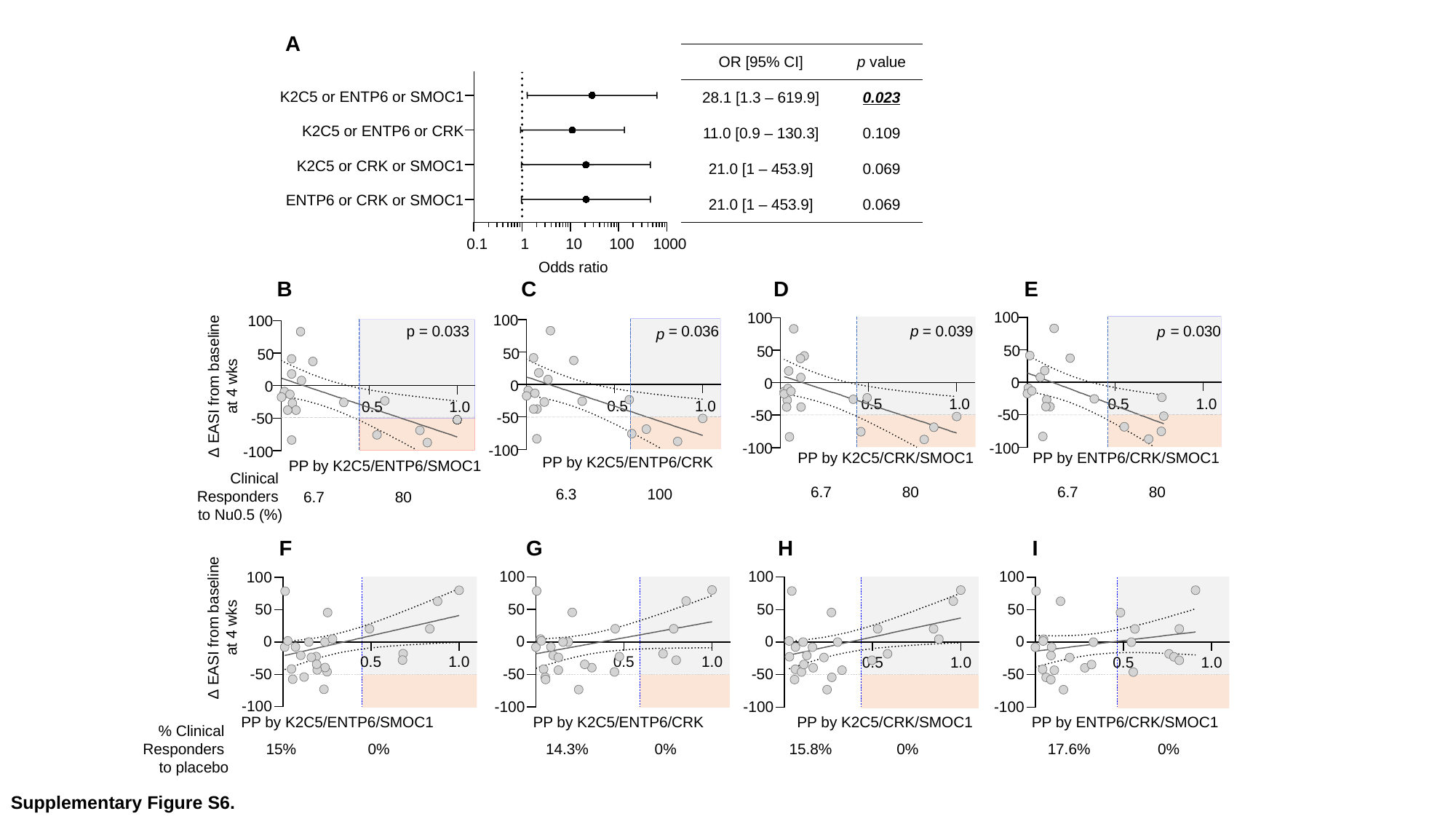

A
K2C5 or ENTP6 or SMOC1
K2C5 or ENTP6 or CRK
K2C5 or CRK or SMOC1
ENTP6 or CRK or SMOC1
0.1
1
10
100
Odds ratio
| OR [95% CI] | p value |
| --- | --- |
| 28.1 [1.3 – 619.9] | 0.023 |
| 11.0 [0.9 – 130.3] | 0.109 |
| 21.0 [1 – 453.9] | 0.069 |
| 21.0 [1 – 453.9] | 0.069 |
1000
B
C
D
E
100
100
100
p
50
0
0.5
1.0
-50
-100
100
50
0
0.5
1.0
-50
-100
 p = 0.033
 = 0.036
p
 = 0.039
 = 0.030
p
50
50
∆ EASI from baseline
at 4 wks
0
0
0.5
1.0
0.5
1.0
-50
-50
-100
-100
PP by K2C5/CRK/SMOC1
PP by ENTP6/CRK/SMOC1
PP by K2C5/ENTP6/CRK
PP by K2C5/ENTP6/SMOC1
Clinical
Responders
to Nu0.5 (%)
6.7
80
6.7
80
6.3
100
6.7
80
F
G
H
I
∆ EASI from baseline
at 4 wks
PP by K2C5/ENTP6/SMOC1
PP by K2C5/ENTP6/CRK
PP by K2C5/CRK/SMOC1
PP by ENTP6/CRK/SMOC1
% Clinical
Responders
to placebo
15%
0%
14.3%
0%
15.8%
0%
17.6%
0%
Supplementary Figure S6.

## Slide 21
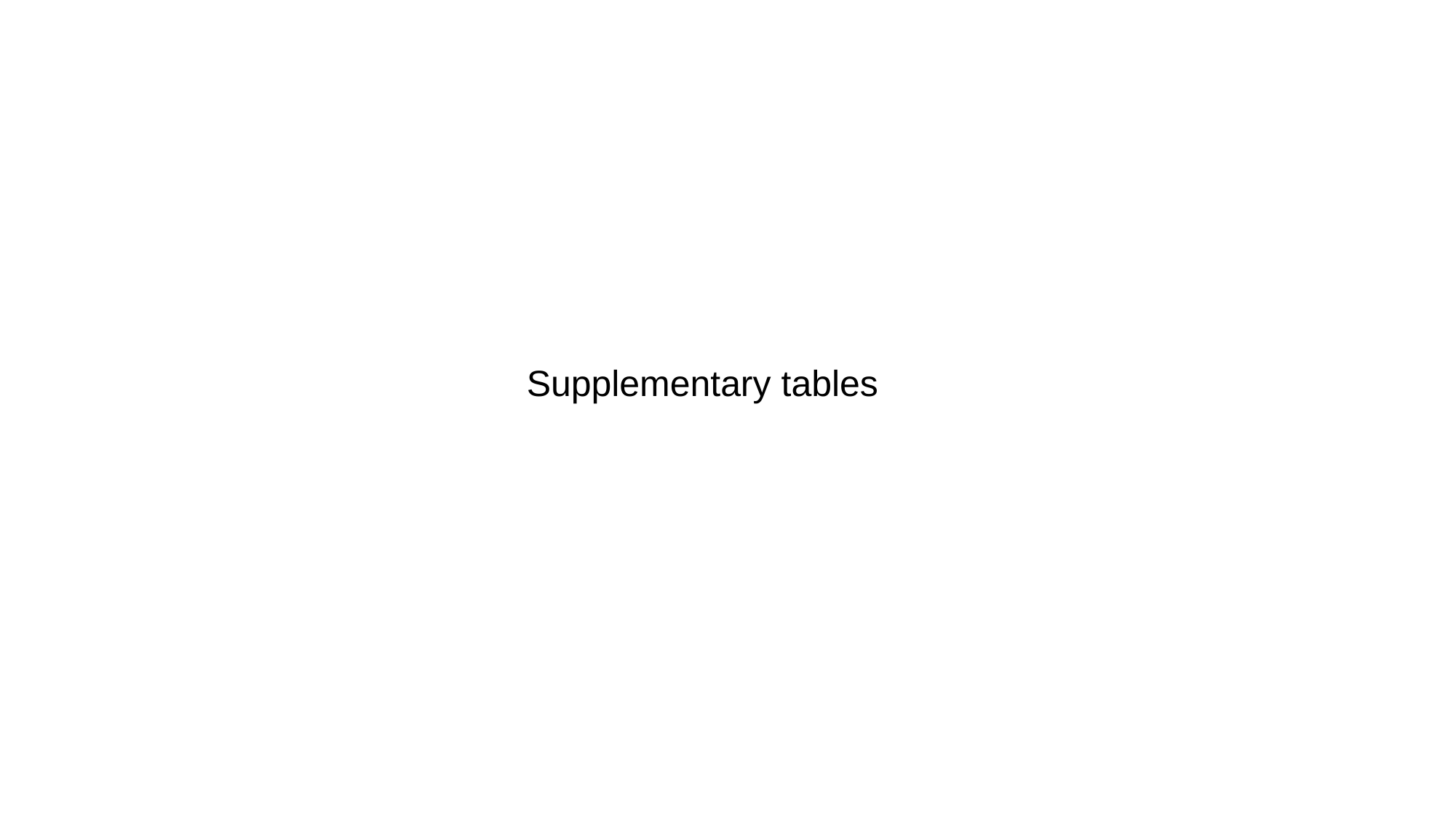

Supplementary tables

## Slide 22
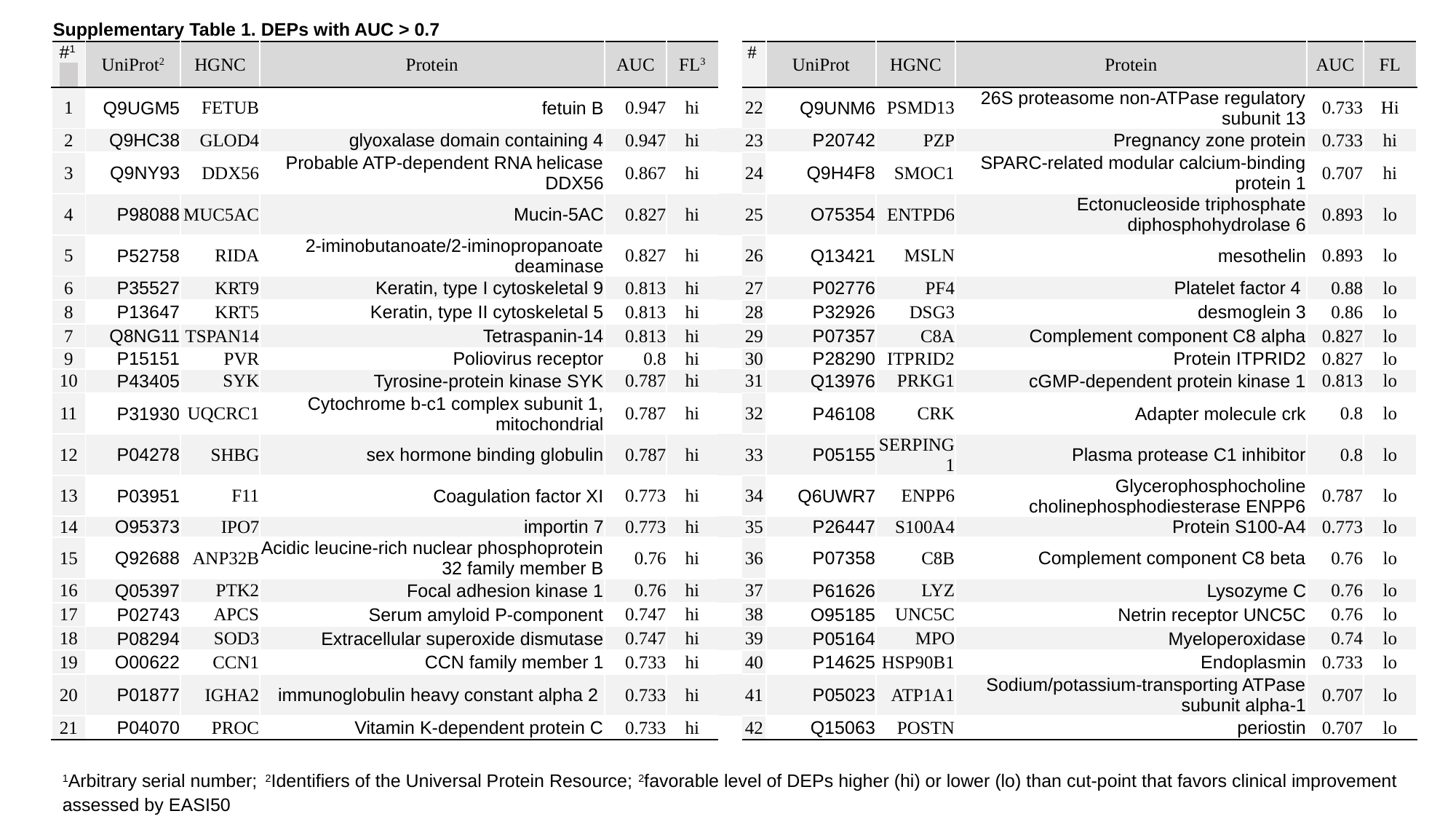

Supplementary Table 1. DEPs with AUC > 0.7
| #1 | UniProt2 | HGNC | Protein | AUC | FL3 | | # | UniProt | HGNC | Protein | AUC | FL |
| --- | --- | --- | --- | --- | --- | --- | --- | --- | --- | --- | --- | --- |
| 1 | Q9UGM5 | FETUB | fetuin B | 0.947 | hi | | 22 | Q9UNM6 | PSMD13 | 26S proteasome non-ATPase regulatory subunit 13 | 0.733 | Hi |
| 2 | Q9HC38 | GLOD4 | glyoxalase domain containing 4 | 0.947 | hi | | 23 | P20742 | PZP | Pregnancy zone protein | 0.733 | hi |
| 3 | Q9NY93 | DDX56 | Probable ATP-dependent RNA helicase DDX56 | 0.867 | hi | | 24 | Q9H4F8 | SMOC1 | SPARC-related modular calcium-binding protein 1 | 0.707 | hi |
| 4 | P98088 | MUC5AC | Mucin-5AC | 0.827 | hi | | 25 | O75354 | ENTPD6 | Ectonucleoside triphosphate diphosphohydrolase 6 | 0.893 | lo |
| 5 | P52758 | RIDA | 2-iminobutanoate/2-iminopropanoate deaminase | 0.827 | hi | | 26 | Q13421 | MSLN | mesothelin | 0.893 | lo |
| 6 | P35527 | KRT9 | Keratin, type I cytoskeletal 9 | 0.813 | hi | | 27 | P02776 | PF4 | Platelet factor 4 | 0.88 | lo |
| 8 | P13647 | KRT5 | Keratin, type II cytoskeletal 5 | 0.813 | hi | | 28 | P32926 | DSG3 | desmoglein 3 | 0.86 | lo |
| 7 | Q8NG11 | TSPAN14 | Tetraspanin-14 | 0.813 | hi | | 29 | P07357 | C8A | Complement component C8 alpha | 0.827 | lo |
| 9 | P15151 | PVR | Poliovirus receptor | 0.8 | hi | | 30 | P28290 | ITPRID2 | Protein ITPRID2 | 0.827 | lo |
| 10 | P43405 | SYK | Tyrosine-protein kinase SYK | 0.787 | hi | | 31 | Q13976 | PRKG1 | cGMP-dependent protein kinase 1 | 0.813 | lo |
| 11 | P31930 | UQCRC1 | Cytochrome b-c1 complex subunit 1, mitochondrial | 0.787 | hi | | 32 | P46108 | CRK | Adapter molecule crk | 0.8 | lo |
| 12 | P04278 | SHBG | sex hormone binding globulin | 0.787 | hi | | 33 | P05155 | SERPING1 | Plasma protease C1 inhibitor | 0.8 | lo |
| 13 | P03951 | F11 | Coagulation factor XI | 0.773 | hi | | 34 | Q6UWR7 | ENPP6 | Glycerophosphocholine cholinephosphodiesterase ENPP6 | 0.787 | lo |
| 14 | O95373 | IPO7 | importin 7 | 0.773 | hi | | 35 | P26447 | S100A4 | Protein S100-A4 | 0.773 | lo |
| 15 | Q92688 | ANP32B | Acidic leucine-rich nuclear phosphoprotein 32 family member B | 0.76 | hi | | 36 | P07358 | C8B | Complement component C8 beta | 0.76 | lo |
| 16 | Q05397 | PTK2 | Focal adhesion kinase 1 | 0.76 | hi | | 37 | P61626 | LYZ | Lysozyme C | 0.76 | lo |
| 17 | P02743 | APCS | Serum amyloid P-component | 0.747 | hi | | 38 | O95185 | UNC5C | Netrin receptor UNC5C | 0.76 | lo |
| 18 | P08294 | SOD3 | Extracellular superoxide dismutase | 0.747 | hi | | 39 | P05164 | MPO | Myeloperoxidase | 0.74 | lo |
| 19 | O00622 | CCN1 | CCN family member 1 | 0.733 | hi | | 40 | P14625 | HSP90B1 | Endoplasmin | 0.733 | lo |
| 20 | P01877 | IGHA2 | immunoglobulin heavy constant alpha 2 | 0.733 | hi | | 41 | P05023 | ATP1A1 | Sodium/potassium-transporting ATPase subunit alpha-1 | 0.707 | lo |
| 21 | P04070 | PROC | Vitamin K-dependent protein C | 0.733 | hi | | 42 | Q15063 | POSTN | periostin | 0.707 | lo |
1Arbitrary serial number; 2Identifiers of the Universal Protein Resource; 2favorable level of DEPs higher (hi) or lower (lo) than cut-point that favors clinical improvement assessed by EASI50

## Slide 23
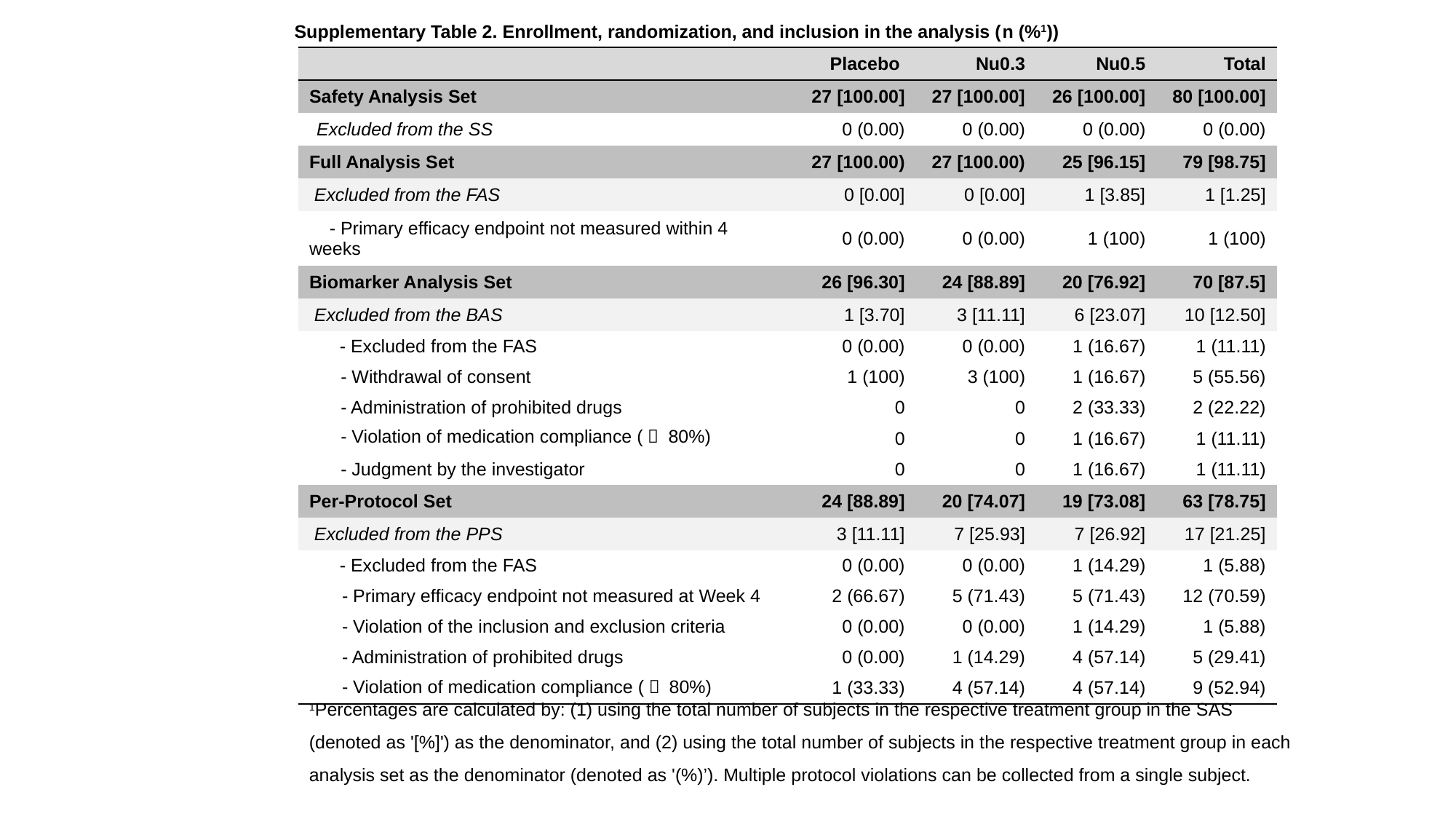

Supplementary Table 2. Enrollment, randomization, and inclusion in the analysis (n (%1))
| | Placebo | Nu0.3 | Nu0.5 | Total |
| --- | --- | --- | --- | --- |
| Safety Analysis Set | 27 [100.00] | 27 [100.00] | 26 [100.00] | 80 [100.00] |
| Excluded from the SS | 0 (0.00) | 0 (0.00) | 0 (0.00) | 0 (0.00) |
| Full Analysis Set | 27 [100.00) | 27 [100.00) | 25 [96.15] | 79 [98.75] |
| Excluded from the FAS | 0 [0.00] | 0 [0.00] | 1 [3.85] | 1 [1.25] |
| - Primary efficacy endpoint not measured within 4 weeks | 0 (0.00) | 0 (0.00) | 1 (100) | 1 (100) |
| Biomarker Analysis Set | 26 [96.30] | 24 [88.89] | 20 [76.92] | 70 [87.5] |
| Excluded from the BAS | 1 [3.70] | 3 [11.11] | 6 [23.07] | 10 [12.50] |
| - Excluded from the FAS | 0 (0.00) | 0 (0.00) | 1 (16.67) | 1 (11.11) |
| - Withdrawal of consent | 1 (100) | 3 (100) | 1 (16.67) | 5 (55.56) |
| - Administration of prohibited drugs | 0 | 0 | 2 (33.33) | 2 (22.22) |
| - Violation of medication compliance (＜ 80%) | 0 | 0 | 1 (16.67) | 1 (11.11) |
| - Judgment by the investigator | 0 | 0 | 1 (16.67) | 1 (11.11) |
| Per-Protocol Set | 24 [88.89] | 20 [74.07] | 19 [73.08] | 63 [78.75] |
| Excluded from the PPS | 3 [11.11] | 7 [25.93] | 7 [26.92] | 17 [21.25] |
| - Excluded from the FAS | 0 (0.00) | 0 (0.00) | 1 (14.29) | 1 (5.88) |
| - Primary efficacy endpoint not measured at Week 4 | 2 (66.67) | 5 (71.43) | 5 (71.43) | 12 (70.59) |
| - Violation of the inclusion and exclusion criteria | 0 (0.00) | 0 (0.00) | 1 (14.29) | 1 (5.88) |
| - Administration of prohibited drugs | 0 (0.00) | 1 (14.29) | 4 (57.14) | 5 (29.41) |
| - Violation of medication compliance (＜ 80%) | 1 (33.33) | 4 (57.14) | 4 (57.14) | 9 (52.94) |
1Percentages are calculated by: (1) using the total number of subjects in the respective treatment group in the SAS (denoted as '[%]') as the denominator, and (2) using the total number of subjects in the respective treatment group in each analysis set as the denominator (denoted as '(%)’). Multiple protocol violations can be collected from a single subject.

## Slide 24
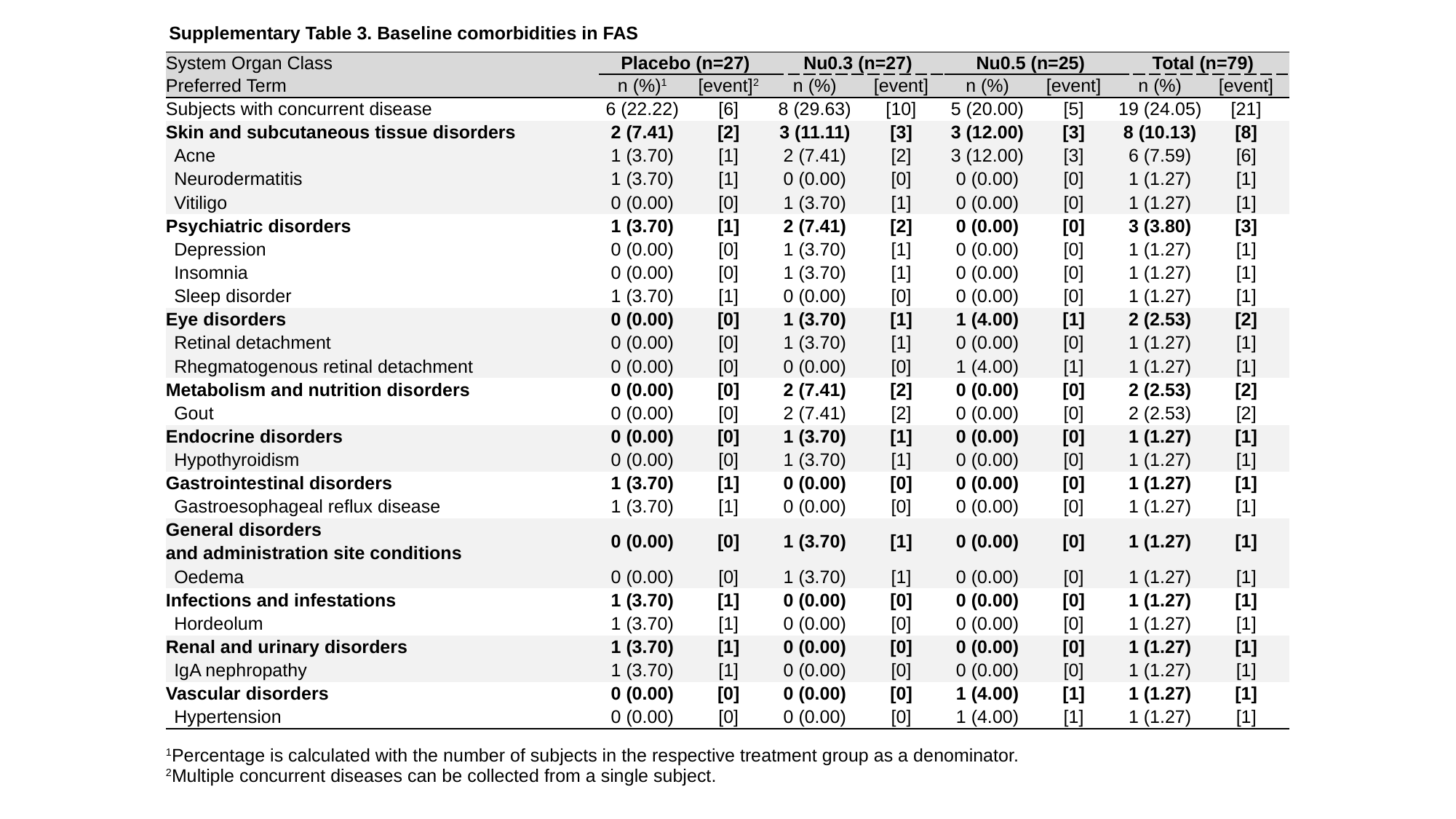

Supplementary Table 3. Baseline comorbidities in FAS
| System Organ Class | Placebo (n=27) | | Nu0.3 (n=27) | | Nu0.5 (n=25) | | Total (n=79) | |
| --- | --- | --- | --- | --- | --- | --- | --- | --- |
| Preferred Term | n (%)1 | [event]2 | n (%) | [event] | n (%) | [event] | n (%) | [event] |
| Subjects with concurrent disease | 6 (22.22) | [6] | 8 (29.63) | [10] | 5 (20.00) | [5] | 19 (24.05) | [21] |
| Skin and subcutaneous tissue disorders | 2 (7.41) | [2] | 3 (11.11) | [3] | 3 (12.00) | [3] | 8 (10.13) | [8] |
| Acne | 1 (3.70) | [1] | 2 (7.41) | [2] | 3 (12.00) | [3] | 6 (7.59) | [6] |
| Neurodermatitis | 1 (3.70) | [1] | 0 (0.00) | [0] | 0 (0.00) | [0] | 1 (1.27) | [1] |
| Vitiligo | 0 (0.00) | [0] | 1 (3.70) | [1] | 0 (0.00) | [0] | 1 (1.27) | [1] |
| Psychiatric disorders | 1 (3.70) | [1] | 2 (7.41) | [2] | 0 (0.00) | [0] | 3 (3.80) | [3] |
| Depression | 0 (0.00) | [0] | 1 (3.70) | [1] | 0 (0.00) | [0] | 1 (1.27) | [1] |
| Insomnia | 0 (0.00) | [0] | 1 (3.70) | [1] | 0 (0.00) | [0] | 1 (1.27) | [1] |
| Sleep disorder | 1 (3.70) | [1] | 0 (0.00) | [0] | 0 (0.00) | [0] | 1 (1.27) | [1] |
| Eye disorders | 0 (0.00) | [0] | 1 (3.70) | [1] | 1 (4.00) | [1] | 2 (2.53) | [2] |
| Retinal detachment | 0 (0.00) | [0] | 1 (3.70) | [1] | 0 (0.00) | [0] | 1 (1.27) | [1] |
| Rhegmatogenous retinal detachment | 0 (0.00) | [0] | 0 (0.00) | [0] | 1 (4.00) | [1] | 1 (1.27) | [1] |
| Metabolism and nutrition disorders | 0 (0.00) | [0] | 2 (7.41) | [2] | 0 (0.00) | [0] | 2 (2.53) | [2] |
| Gout | 0 (0.00) | [0] | 2 (7.41) | [2] | 0 (0.00) | [0] | 2 (2.53) | [2] |
| Endocrine disorders | 0 (0.00) | [0] | 1 (3.70) | [1] | 0 (0.00) | [0] | 1 (1.27) | [1] |
| Hypothyroidism | 0 (0.00) | [0] | 1 (3.70) | [1] | 0 (0.00) | [0] | 1 (1.27) | [1] |
| Gastrointestinal disorders | 1 (3.70) | [1] | 0 (0.00) | [0] | 0 (0.00) | [0] | 1 (1.27) | [1] |
| Gastroesophageal reflux disease | 1 (3.70) | [1] | 0 (0.00) | [0] | 0 (0.00) | [0] | 1 (1.27) | [1] |
| General disorders and administration site conditions | 0 (0.00) | [0] | 1 (3.70) | [1] | 0 (0.00) | [0] | 1 (1.27) | [1] |
| Oedema | 0 (0.00) | [0] | 1 (3.70) | [1] | 0 (0.00) | [0] | 1 (1.27) | [1] |
| Infections and infestations | 1 (3.70) | [1] | 0 (0.00) | [0] | 0 (0.00) | [0] | 1 (1.27) | [1] |
| Hordeolum | 1 (3.70) | [1] | 0 (0.00) | [0] | 0 (0.00) | [0] | 1 (1.27) | [1] |
| Renal and urinary disorders | 1 (3.70) | [1] | 0 (0.00) | [0] | 0 (0.00) | [0] | 1 (1.27) | [1] |
| IgA nephropathy | 1 (3.70) | [1] | 0 (0.00) | [0] | 0 (0.00) | [0] | 1 (1.27) | [1] |
| Vascular disorders | 0 (0.00) | [0] | 0 (0.00) | [0] | 1 (4.00) | [1] | 1 (1.27) | [1] |
| Hypertension | 0 (0.00) | [0] | 0 (0.00) | [0] | 1 (4.00) | [1] | 1 (1.27) | [1] |
| 1Percentage is calculated with the number of subjects in the respective treatment group as a denominator. 2Multiple concurrent diseases can be collected from a single subject. | | | | | | | | |

## Slide 25
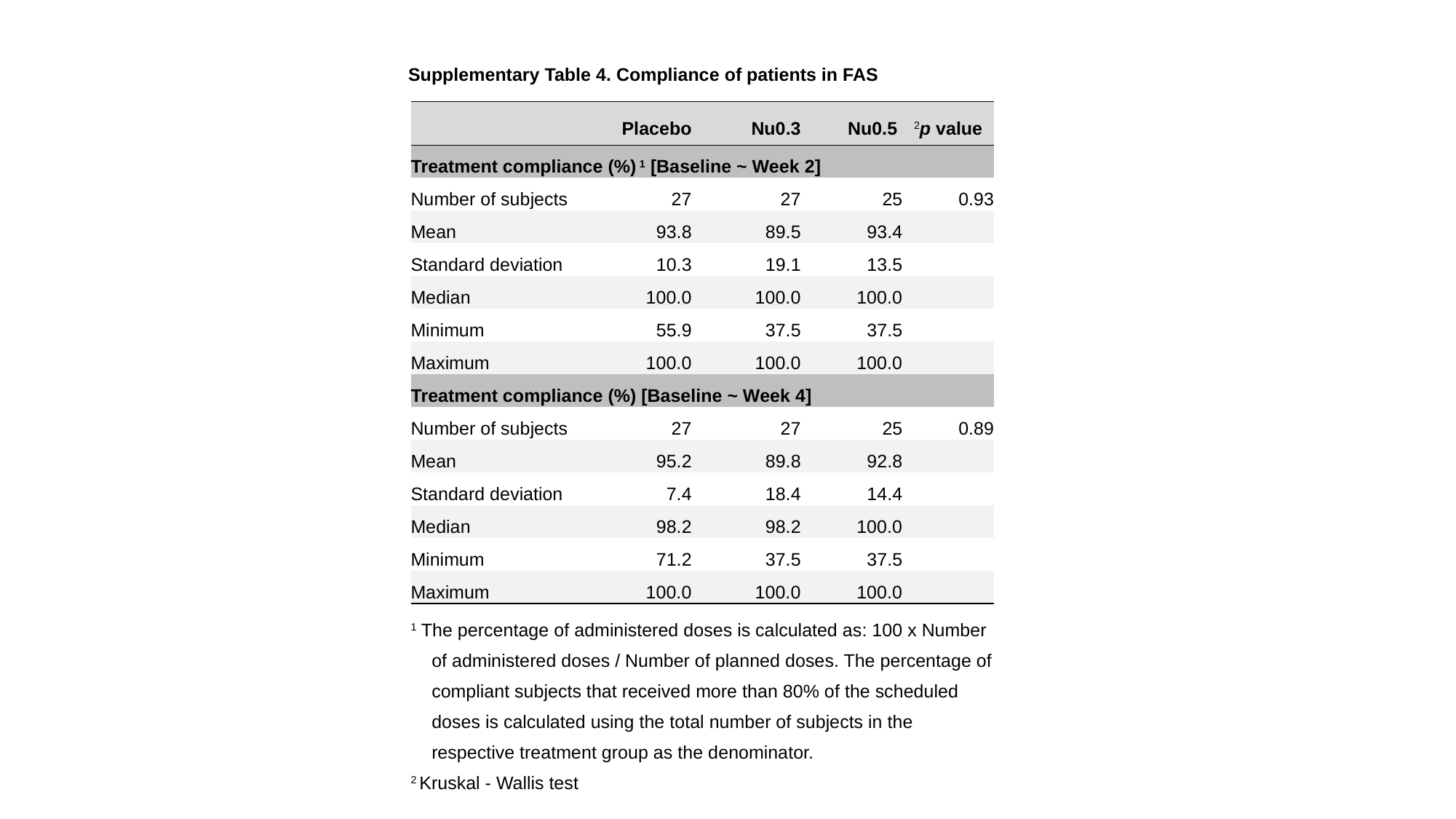

Supplementary Table 4. Compliance of patients in FAS
| | Placebo | Nu0.3 | Nu0.5 | 2p value |
| --- | --- | --- | --- | --- |
| Treatment compliance (%) 1 [Baseline ~ Week 2] | | | | |
| Number of subjects | 27 | 27 | 25 | 0.93 |
| Mean | 93.8 | 89.5 | 93.4 | |
| Standard deviation | 10.3 | 19.1 | 13.5 | |
| Median | 100.0 | 100.0 | 100.0 | |
| Minimum | 55.9 | 37.5 | 37.5 | |
| Maximum | 100.0 | 100.0 | 100.0 | |
| Treatment compliance (%) [Baseline ~ Week 4] | | | | |
| Number of subjects | 27 | 27 | 25 | 0.89 |
| Mean | 95.2 | 89.8 | 92.8 | |
| Standard deviation | 7.4 | 18.4 | 14.4 | |
| Median | 98.2 | 98.2 | 100.0 | |
| Minimum | 71.2 | 37.5 | 37.5 | |
| Maximum | 100.0 | 100.0 | 100.0 | |
| 1 The percentage of administered doses is calculated as: 100 x Number of administered doses / Number of planned doses. The percentage of compliant subjects that received more than 80% of the scheduled doses is calculated using the total number of subjects in the respective treatment group as the denominator. 2 Kruskal - Wallis test | | | | |

## Slide 26
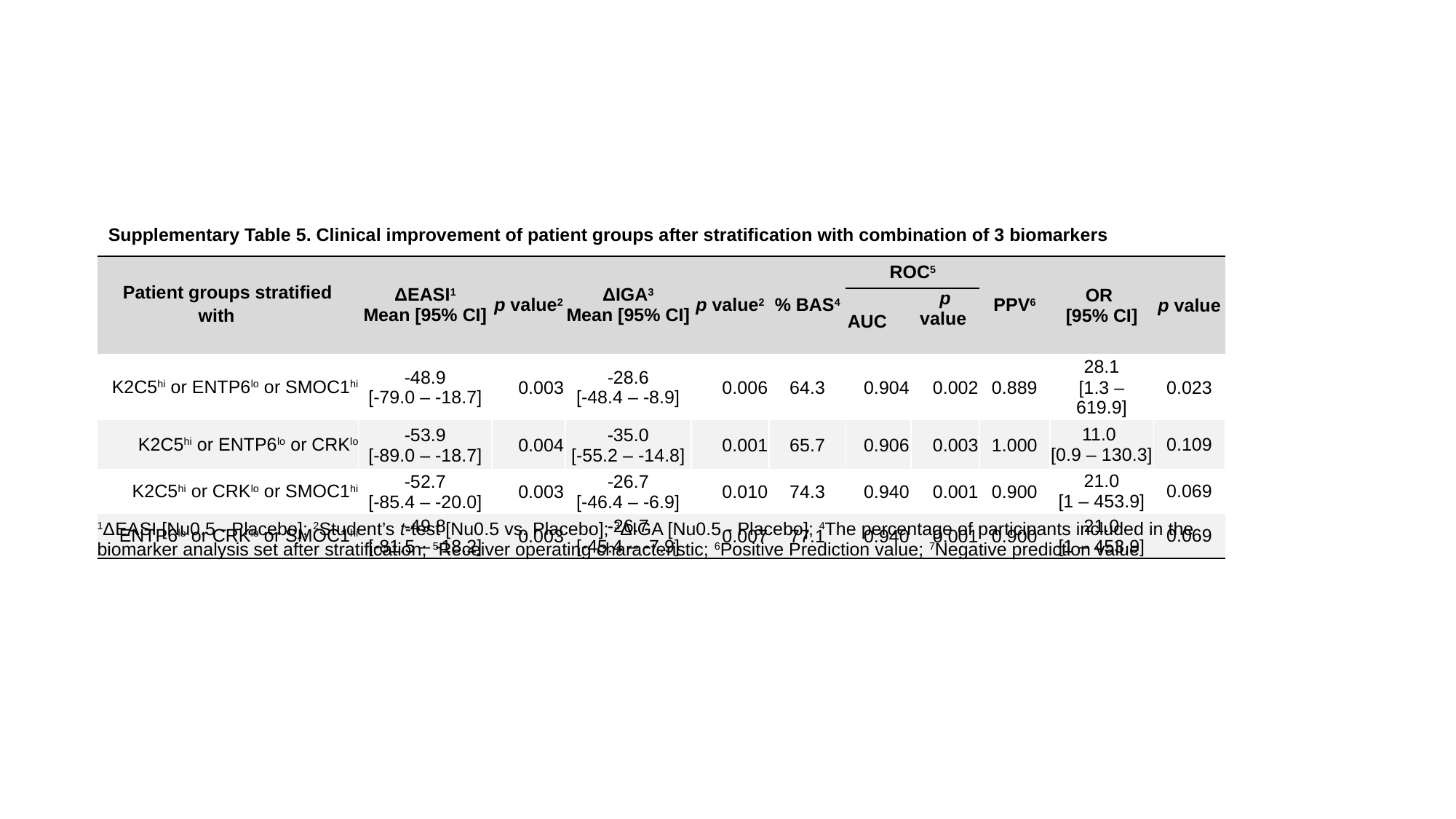

Supplementary Table 5. Clinical improvement of patient groups after stratification with combination of 3 biomarkers
| Patient groups stratified with | ΔEASI1 Mean [95% CI] | p value2 | ΔIGA3 Mean [95% CI] | p value2 | % BAS4 | ROC5 | | PPV6 | OR [95% CI] | p value |
| --- | --- | --- | --- | --- | --- | --- | --- | --- | --- | --- |
| | | | | | | AUC | p value | | | |
| K2C5hi or ENTP6lo or SMOC1hi | -48.9 [-79.0 – -18.7] | 0.003 | -28.6 [-48.4 – -8.9] | 0.006 | 64.3 | 0.904 | 0.002 | 0.889 | 28.1 [1.3 – 619.9] | 0.023 |
| K2C5hi or ENTP6lo or CRKlo | -53.9 [-89.0 – -18.7] | 0.004 | -35.0 [-55.2 – -14.8] | 0.001 | 65.7 | 0.906 | 0.003 | 1.000 | 11.0 [0.9 – 130.3] | 0.109 |
| K2C5hi or CRKlo or SMOC1hi | -52.7 [-85.4 – -20.0] | 0.003 | -26.7 [-46.4 – -6.9] | 0.010 | 74.3 | 0.940 | 0.001 | 0.900 | 21.0 [1 – 453.9] | 0.069 |
| ENTP6lo or CRKlo or SMOC1hi | -49.8 [-81.5 – -18.2] | 0.003 | -26.7 [-45.4 – -7.9] | 0.007 | 77.1 | 0.940 | 0.001 | 0.900 | 21.0 [1 – 453.9] | 0.069 |
| 1ΔEASI [Nu0.5 - Placebo]; 2Student’s t-test [Nu0.5 vs. Placebo]; 3ΔIGA [Nu0.5 - Placebo]; 4The percentage of participants included in the biomarker analysis set after stratification; 5Receiver operating characteristic; 6Positive Prediction value; 7Negative prediction value |
| --- |

## Slide 27
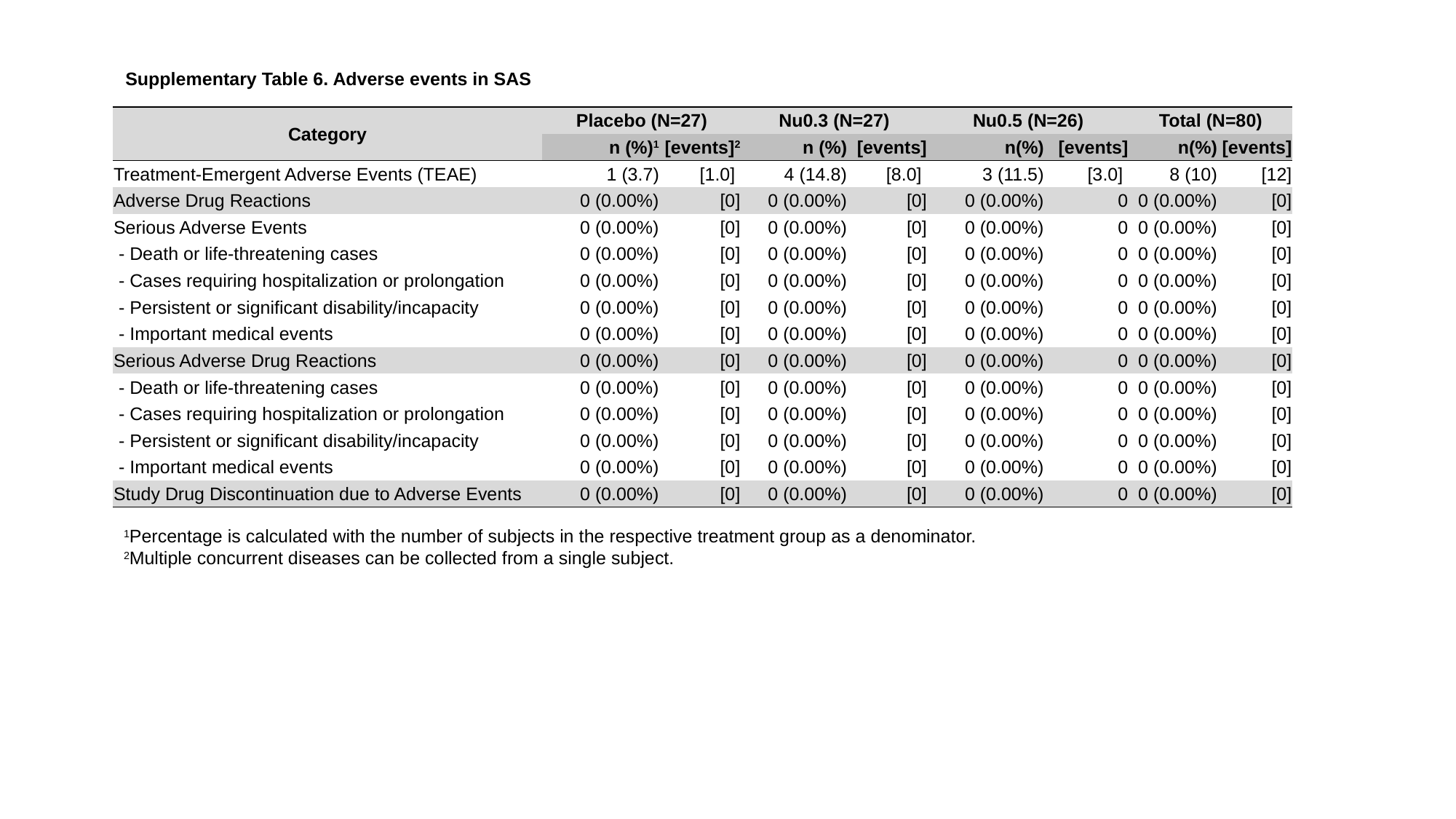

Supplementary Table 6. Adverse events in SAS
| Category | Placebo (N=27) | | Nu0.3 (N=27) | | Nu0.5 (N=26) | | Total (N=80) | |
| --- | --- | --- | --- | --- | --- | --- | --- | --- |
| | n (%)1 | [events]2 | n (%) | [events] | n(%) | [events] | n(%) | [events] |
| Treatment-Emergent Adverse Events (TEAE) | 1 (3.7) | [1.0] | 4 (14.8) | [8.0] | 3 (11.5) | [3.0] | 8 (10) | [12] |
| Adverse Drug Reactions | 0 (0.00%) | [0] | 0 (0.00%) | [0] | 0 (0.00%) | 0 | 0 (0.00%) | [0] |
| Serious Adverse Events | 0 (0.00%) | [0] | 0 (0.00%) | [0] | 0 (0.00%) | 0 | 0 (0.00%) | [0] |
| - Death or life-threatening cases | 0 (0.00%) | [0] | 0 (0.00%) | [0] | 0 (0.00%) | 0 | 0 (0.00%) | [0] |
| - Cases requiring hospitalization or prolongation | 0 (0.00%) | [0] | 0 (0.00%) | [0] | 0 (0.00%) | 0 | 0 (0.00%) | [0] |
| - Persistent or significant disability/incapacity | 0 (0.00%) | [0] | 0 (0.00%) | [0] | 0 (0.00%) | 0 | 0 (0.00%) | [0] |
| - Important medical events | 0 (0.00%) | [0] | 0 (0.00%) | [0] | 0 (0.00%) | 0 | 0 (0.00%) | [0] |
| Serious Adverse Drug Reactions | 0 (0.00%) | [0] | 0 (0.00%) | [0] | 0 (0.00%) | 0 | 0 (0.00%) | [0] |
| - Death or life-threatening cases | 0 (0.00%) | [0] | 0 (0.00%) | [0] | 0 (0.00%) | 0 | 0 (0.00%) | [0] |
| - Cases requiring hospitalization or prolongation | 0 (0.00%) | [0] | 0 (0.00%) | [0] | 0 (0.00%) | 0 | 0 (0.00%) | [0] |
| - Persistent or significant disability/incapacity | 0 (0.00%) | [0] | 0 (0.00%) | [0] | 0 (0.00%) | 0 | 0 (0.00%) | [0] |
| - Important medical events | 0 (0.00%) | [0] | 0 (0.00%) | [0] | 0 (0.00%) | 0 | 0 (0.00%) | [0] |
| Study Drug Discontinuation due to Adverse Events | 0 (0.00%) | [0] | 0 (0.00%) | [0] | 0 (0.00%) | 0 | 0 (0.00%) | [0] |
1Percentage is calculated with the number of subjects in the respective treatment group as a denominator.
2Multiple concurrent diseases can be collected from a single subject.
