# Supplementary figures and images for: A phase 2a double-blind, placebo-controlled, randomized clinical trial evaluating the efficacy and safety of NuGel, a novel topical GPCR19-mediated inflammasome inhibitor, in patients with mild to moderate atopic dermatitis: a proof-of-concept study with Post-hoc biomarker analysis
Source: Front Immunol. 2025 May 19;16:1560447. doi: 10.3389/fimmu.2025.1560447 (PMC12127193; doi:10.3389/fimmu.2025.1560447)

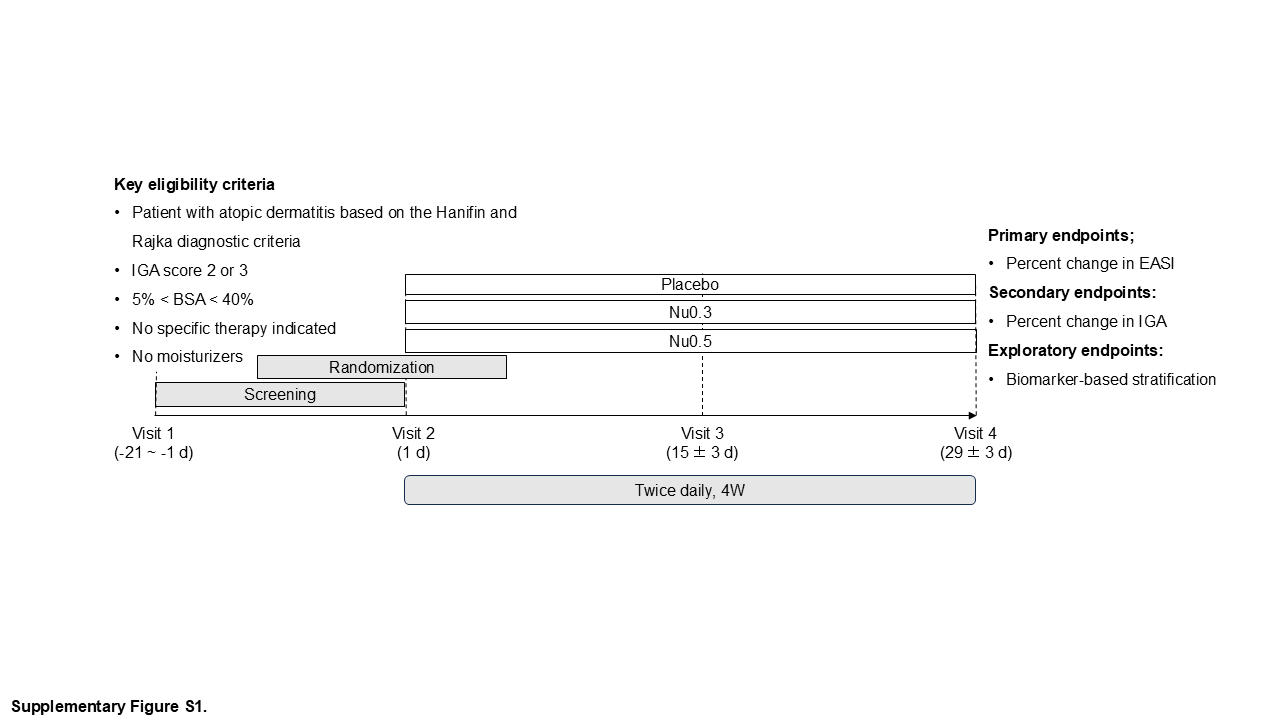

Supplement: Supplementary file 2 [file Presentation2.zip › 슬라이드15.TIF]

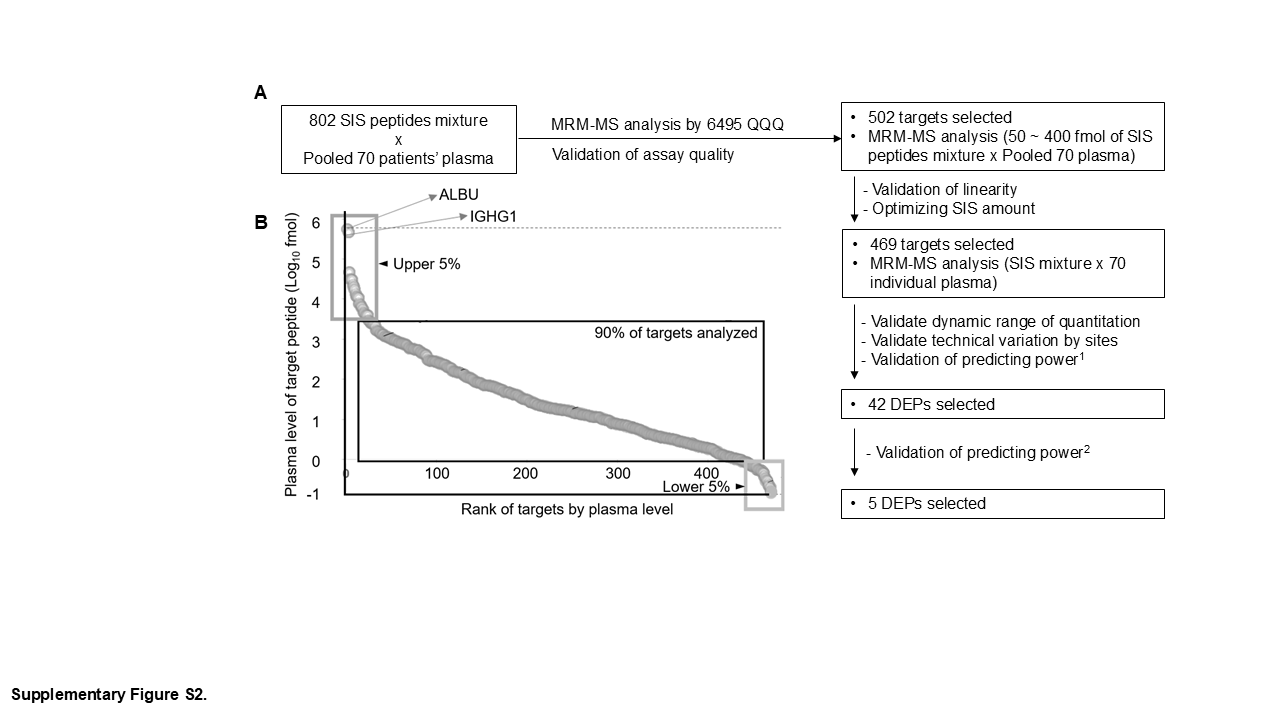

Supplement: Supplementary file 2 [file Presentation2.zip › 슬라이드16.TIF]

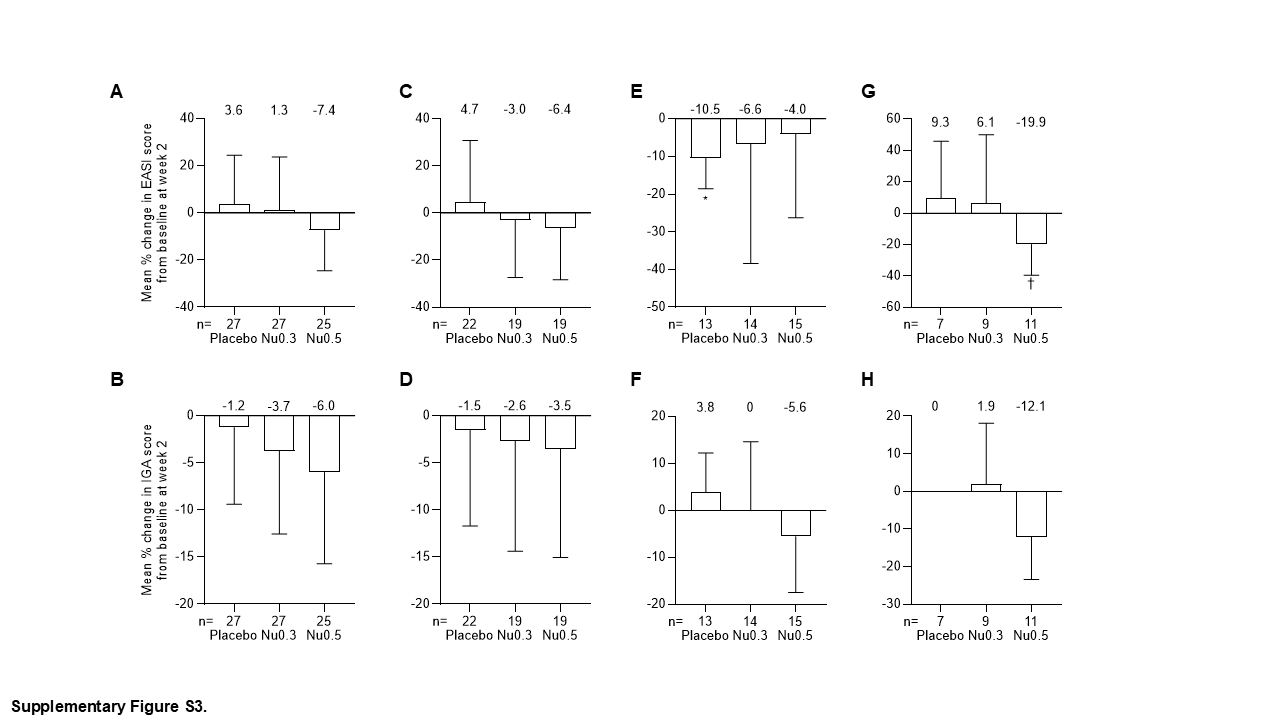

Supplement: Supplementary file 2 [file Presentation2.zip › 슬라이드17.TIF]

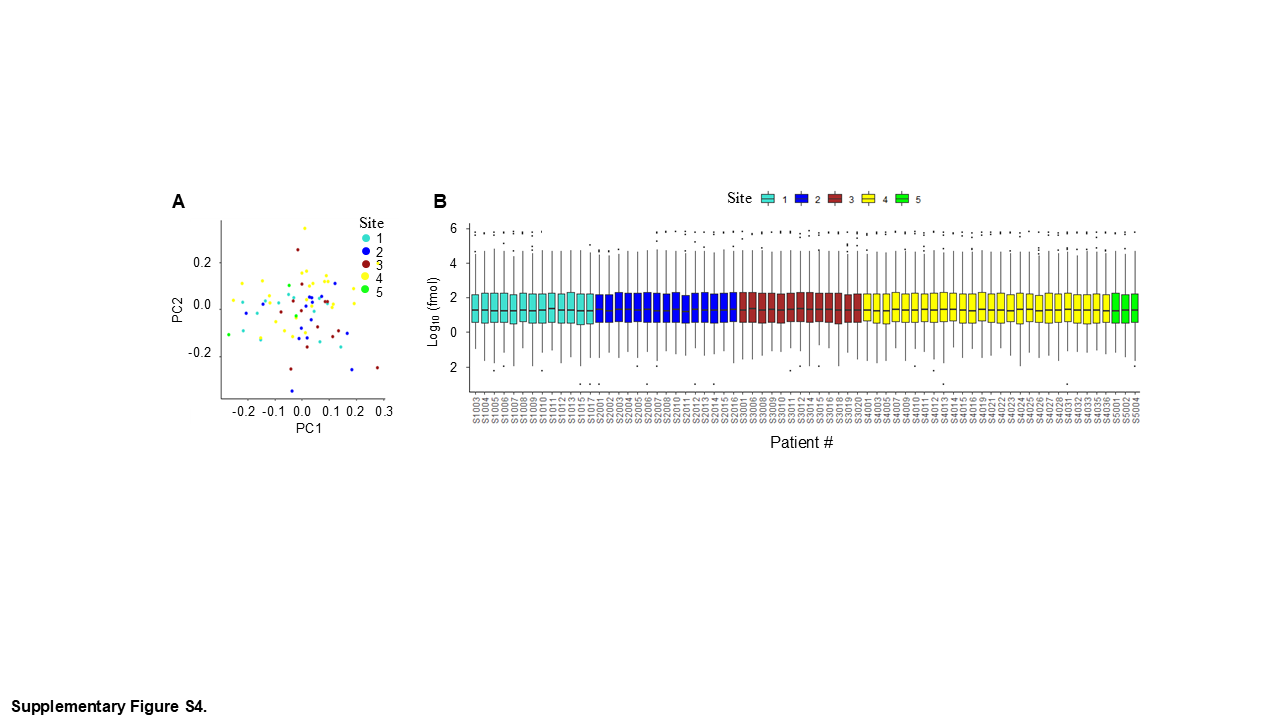

Supplement: Supplementary file 2 [file Presentation2.zip › 슬라이드18.TIF]

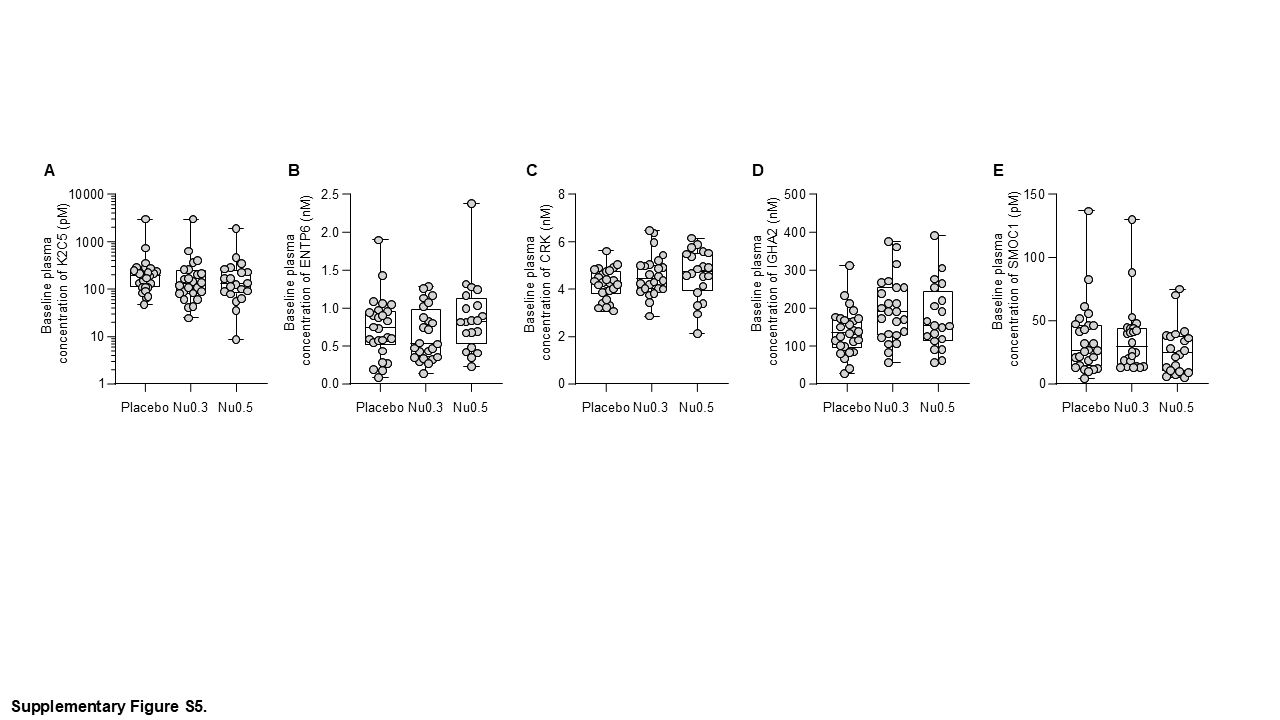

Supplement: Supplementary file 2 [file Presentation2.zip › 슬라이드19.TIF]

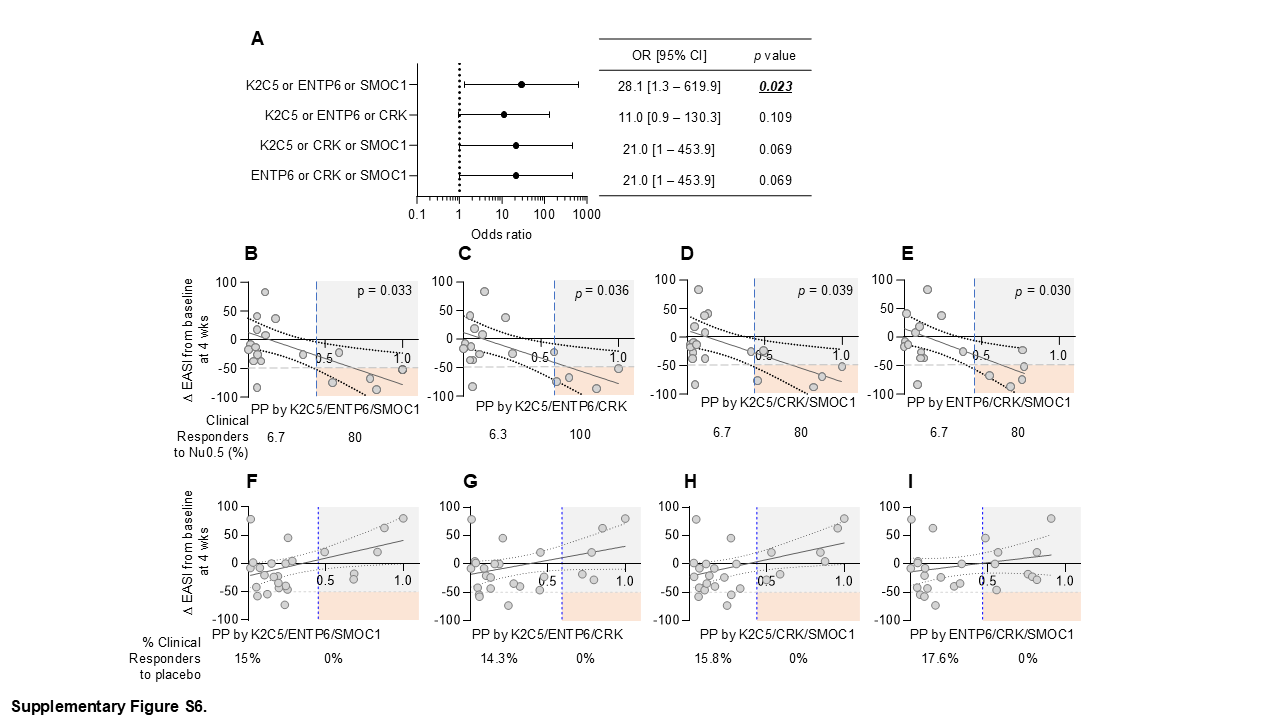

Supplement: Supplementary file 2 [file Presentation2.zip › 슬라이드20.TIF]
