# Supplementary material for: A phase 2a double-blind, placebo-controlled, randomized clinical trial evaluating the efficacy and safety of NuGel, a novel topical GPCR19-mediated inflammasome inhibitor, in patients with mild to moderate atopic dermatitis: a proof-of-concept study with Post-hoc biomarker analysis
Source: Front Immunol. 2025 May 19;16:1560447. doi: 10.3389/fimmu.2025.1560447 (PMC12127193; doi:10.3389/fimmu.2025.1560447)
Supplement: Supplementary file 3 [file Presentation3.pptx]

## Slide 1
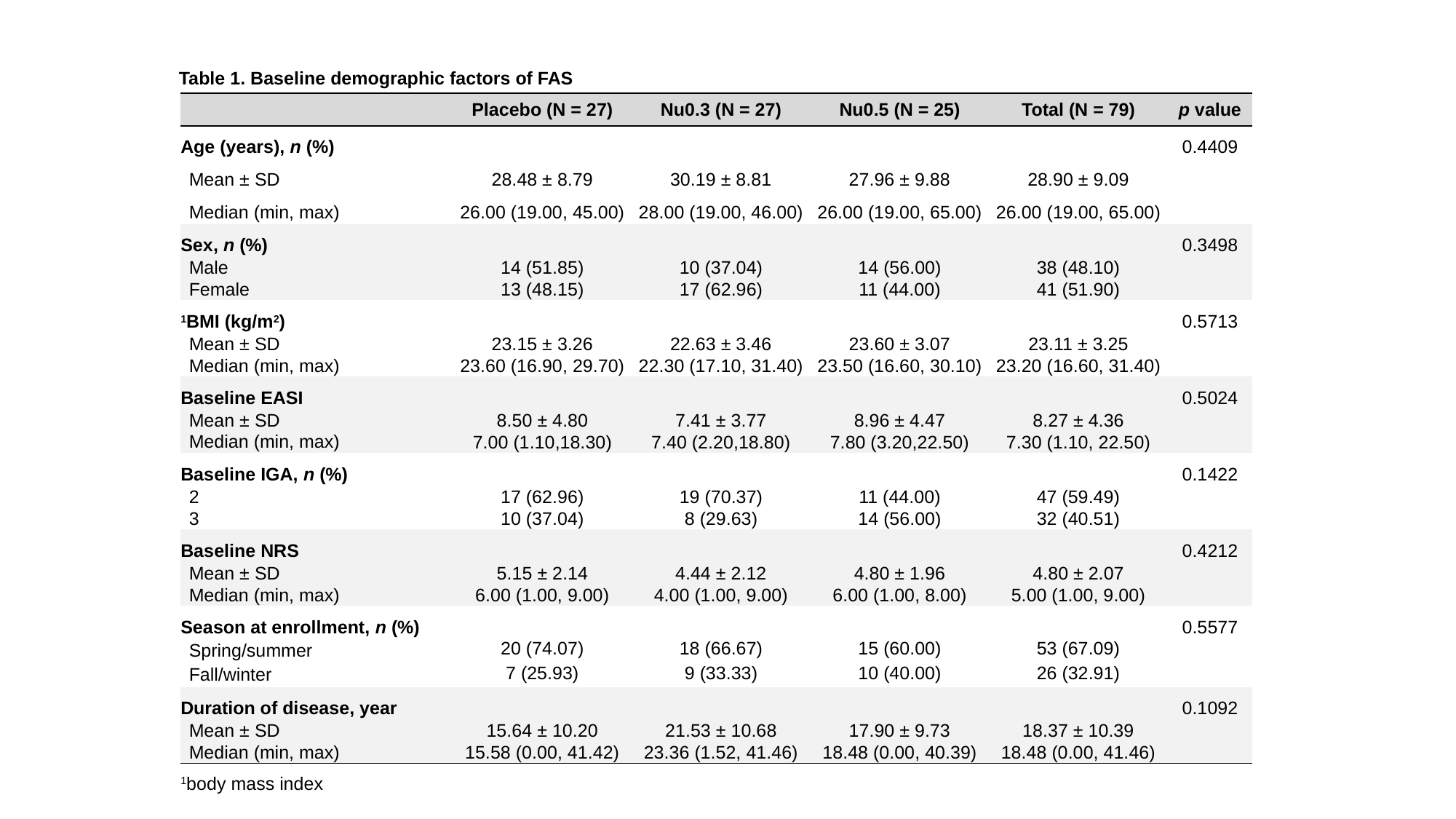

Table 1. Baseline demographic factors of FAS
| | Placebo (N = 27) | Nu0.3 (N = 27) | Nu0.5 (N = 25) | Total (N = 79) | p value |
| --- | --- | --- | --- | --- | --- |
| Age (years), n (%) | | | | | 0.4409 |
| Mean ± SD | 28.48 ± 8.79 | 30.19 ± 8.81 | 27.96 ± 9.88 | 28.90 ± 9.09 | |
| Median (min, max) | 26.00 (19.00, 45.00) | 28.00 (19.00, 46.00) | 26.00 (19.00, 65.00) | 26.00 (19.00, 65.00) | |
| Sex, n (%) | | | | | 0.3498 |
| Male | 14 (51.85) | 10 (37.04) | 14 (56.00) | 38 (48.10) | |
| Female | 13 (48.15) | 17 (62.96) | 11 (44.00) | 41 (51.90) | |
| 1BMI (kg/m2) | | | | | 0.5713 |
| Mean ± SD | 23.15 ± 3.26 | 22.63 ± 3.46 | 23.60 ± 3.07 | 23.11 ± 3.25 | |
| Median (min, max) | 23.60 (16.90, 29.70) | 22.30 (17.10, 31.40) | 23.50 (16.60, 30.10) | 23.20 (16.60, 31.40) | |
| Baseline EASI | | | | | 0.5024 |
| Mean ± SD | 8.50 ± 4.80 | 7.41 ± 3.77 | 8.96 ± 4.47 | 8.27 ± 4.36 | |
| Median (min, max) | 7.00 (1.10,18.30) | 7.40 (2.20,18.80) | 7.80 (3.20,22.50) | 7.30 (1.10, 22.50) | |
| Baseline IGA, n (%) | | | | | 0.1422 |
| 2 | 17 (62.96) | 19 (70.37) | 11 (44.00) | 47 (59.49) | |
| 3 | 10 (37.04) | 8 (29.63) | 14 (56.00) | 32 (40.51) | |
| Baseline NRS | | | | | 0.4212 |
| Mean ± SD | 5.15 ± 2.14 | 4.44 ± 2.12 | 4.80 ± 1.96 | 4.80 ± 2.07 | |
| Median (min, max) | 6.00 (1.00, 9.00) | 4.00 (1.00, 9.00) | 6.00 (1.00, 8.00) | 5.00 (1.00, 9.00) | |
| Season at enrollment, n (%) | | | | | 0.5577 |
| Spring/summer | 20 (74.07) | 18 (66.67) | 15 (60.00) | 53 (67.09) | |
| Fall/winter | 7 (25.93) | 9 (33.33) | 10 (40.00) | 26 (32.91) | |
| Duration of disease, year | | | | | 0.1092 |
| Mean ± SD | 15.64 ± 10.20 | 21.53 ± 10.68 | 17.90 ± 9.73 | 18.37 ± 10.39 | |
| Median (min, max) | 15.58 (0.00, 41.42) | 23.36 (1.52, 41.46) | 18.48 (0.00, 40.39) | 18.48 (0.00, 41.46) | |
| 1body mass index | | | | | |

## Slide 2
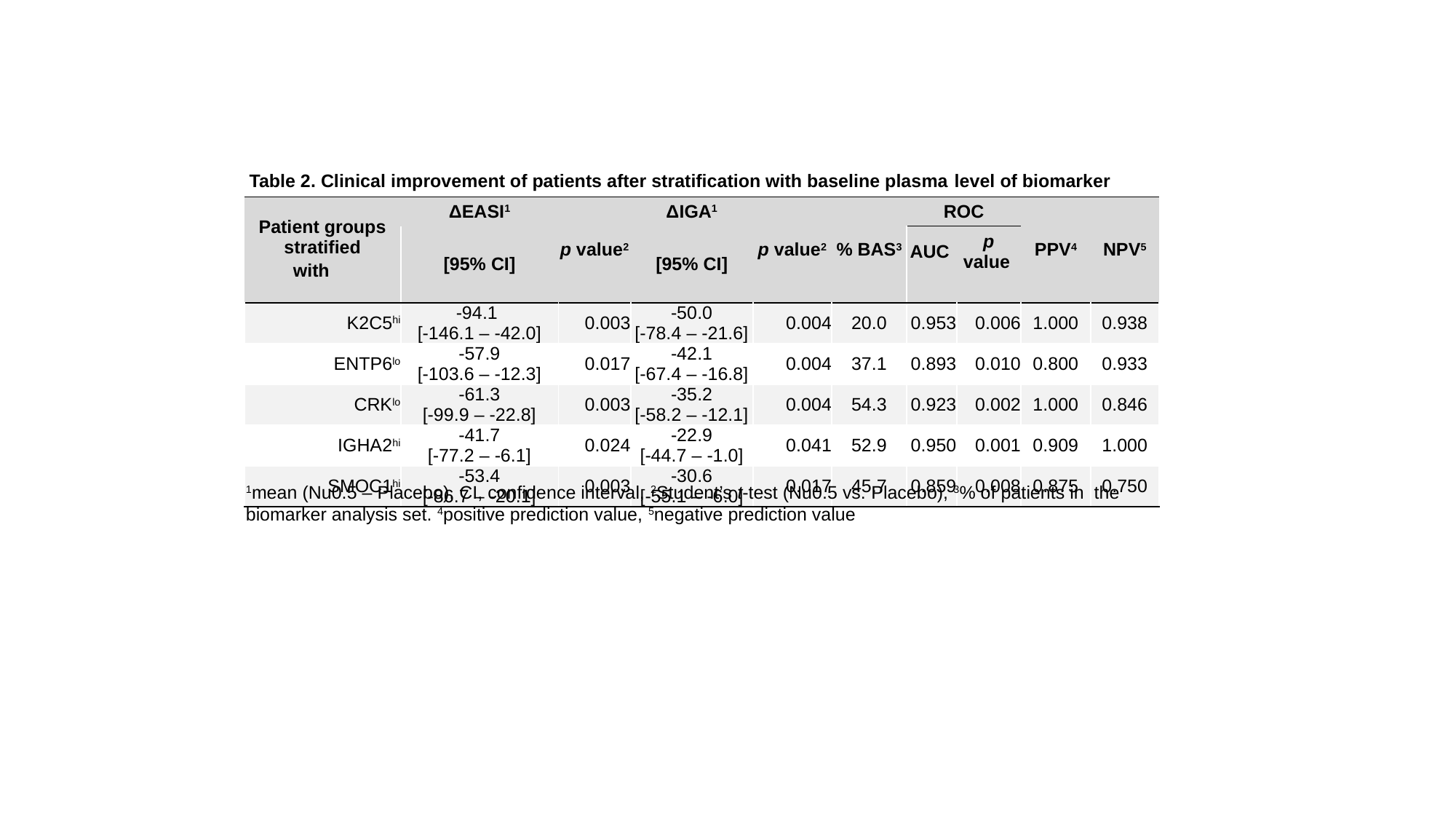

Table 2. Clinical improvement of patients after stratification with baseline plasma level of biomarker
| Patient groups stratified with | ΔEASI1 | p value2 | ΔIGA1 | p value2 | % BAS3 | ROC | | PPV4 | NPV5 |
| --- | --- | --- | --- | --- | --- | --- | --- | --- | --- |
| | [95% CI] | | [95% CI] | | | AUC | p value | | |
| K2C5hi | -94.1 [-146.1 – -42.0] | 0.003 | -50.0 [-78.4 – -21.6] | 0.004 | 20.0 | 0.953 | 0.006 | 1.000 | 0.938 |
| ENTP6lo | -57.9 [-103.6 – -12.3] | 0.017 | -42.1 [-67.4 – -16.8] | 0.004 | 37.1 | 0.893 | 0.010 | 0.800 | 0.933 |
| CRKlo | -61.3 [-99.9 – -22.8] | 0.003 | -35.2 [-58.2 – -12.1] | 0.004 | 54.3 | 0.923 | 0.002 | 1.000 | 0.846 |
| IGHA2hi | -41.7 [-77.2 – -6.1] | 0.024 | -22.9 [-44.7 – -1.0] | 0.041 | 52.9 | 0.950 | 0.001 | 0.909 | 1.000 |
| SMOC1hi | -53.4 [-86.7 – -20.1] | 0.003 | -30.6 [-55.1 – -6.0] | 0.017 | 45.7 | 0.859 | 0.008 | 0.875 | 0.750 |
1mean (Nu0.5 – Placebo), CI, confidence interval, 2Student’s t-test (Nu0.5 vs. Placebo), 3% of patients in the biomarker analysis set. 4positive prediction value, 5negative prediction value

## Slide 3
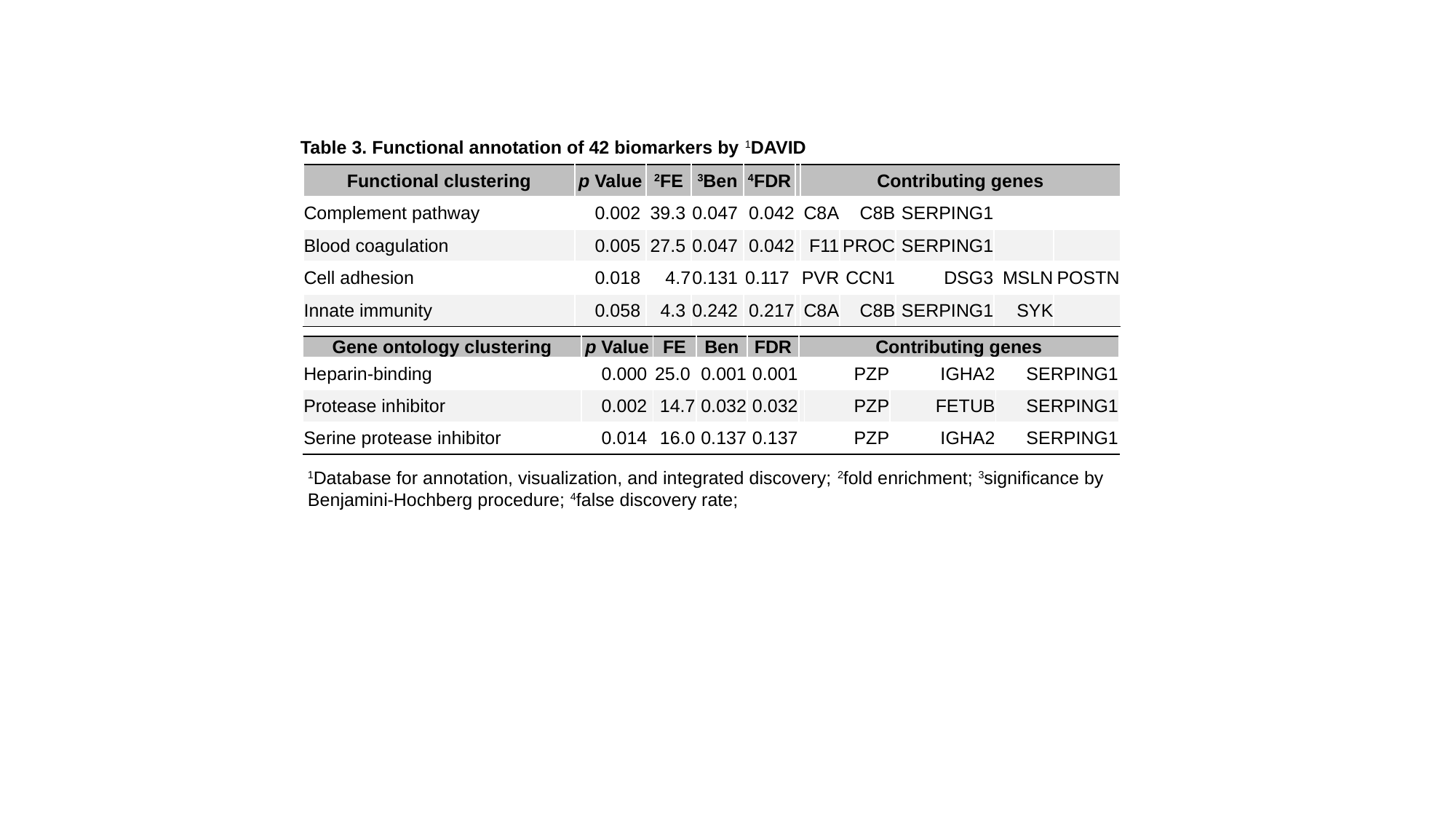

Table 3. Functional annotation of 42 biomarkers by 1DAVID
| Functional clustering | p Value | 2FE | 3Ben | 4FDR | | Contributing genes | | | | |
| --- | --- | --- | --- | --- | --- | --- | --- | --- | --- | --- |
| Complement pathway | 0.002 | 39.3 | 0.047 | 0.042 | | C8A | C8B | SERPING1 | | |
| Blood coagulation | 0.005 | 27.5 | 0.047 | 0.042 | | F11 | PROC | SERPING1 | | |
| Cell adhesion | 0.018 | 4.7 | 0.131 | 0.117 | | PVR | CCN1 | DSG3 | MSLN | POSTN |
| Innate immunity | 0.058 | 4.3 | 0.242 | 0.217 | | C8A | C8B | SERPING1 | SYK | |
| Gene ontology clustering | p Value | FE | Ben | FDR | Contributing genes | | | |
| --- | --- | --- | --- | --- | --- | --- | --- | --- |
| Heparin-binding | 0.000 | 25.0 | 0.001 | 0.001 | | PZP | IGHA2 | SERPING1 |
| Protease inhibitor | 0.002 | 14.7 | 0.032 | 0.032 | | PZP | FETUB | SERPING1 |
| Serine protease inhibitor | 0.014 | 16.0 | 0.137 | 0.137 | | PZP | IGHA2 | SERPING1 |
1Database for annotation, visualization, and integrated discovery; 2fold enrichment; 3significance by Benjamini-Hochberg procedure; 4false discovery rate;

## Slide 4
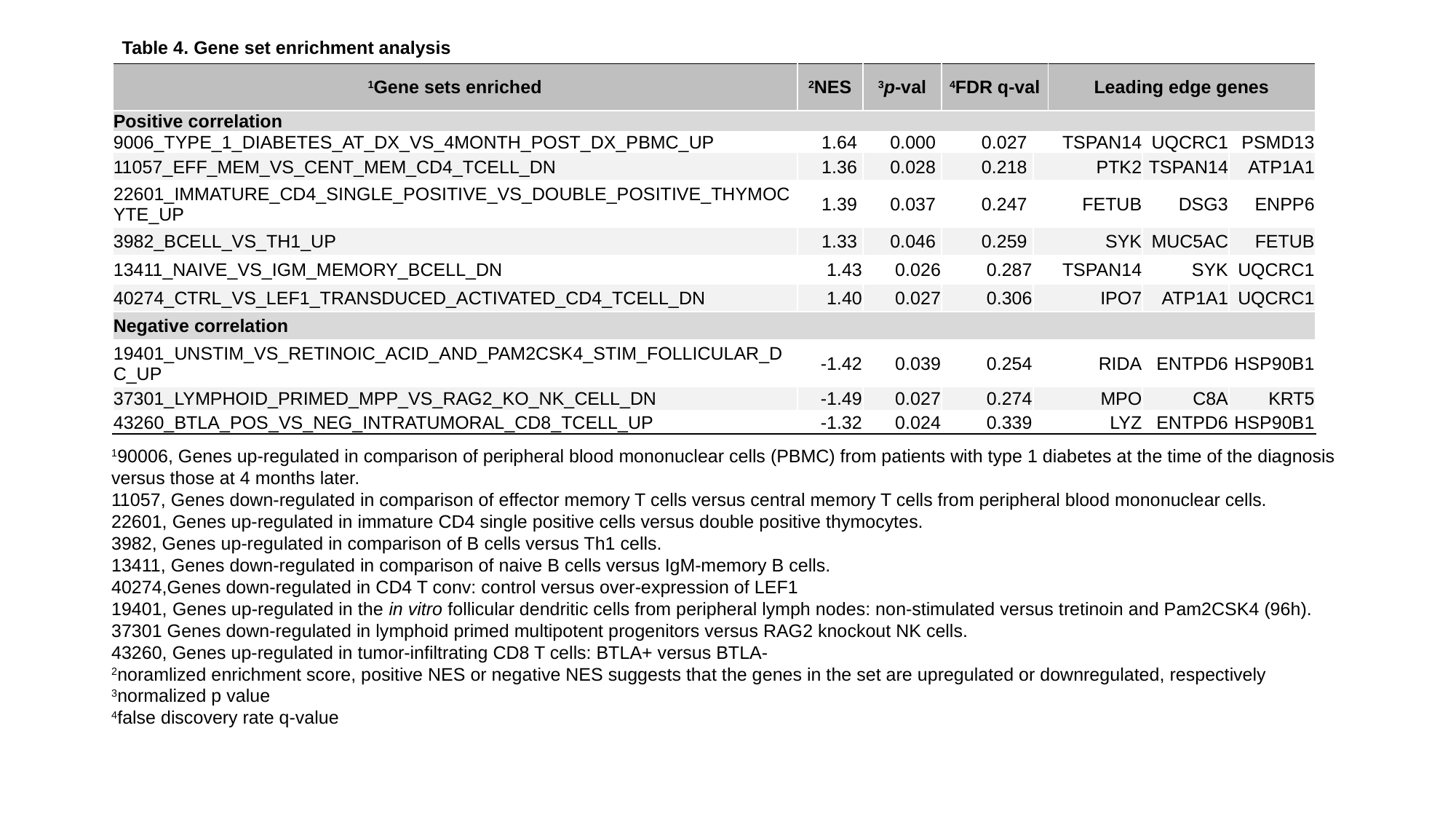

Table 4. Gene set enrichment analysis
| 1Gene sets enriched | 2NES | 3p-val | 4FDR q-val | Leading edge genes | Leading edge genes | | |
| --- | --- | --- | --- | --- | --- | --- | --- |
| Positive correlation | | | | | | | |
| 9006\_TYPE\_1\_DIABETES\_AT\_DX\_VS\_4MONTH\_POST\_DX\_PBMC\_UP | 1.64 | 0.000 | 0.027 | TSPAN14 | | UQCRC1 | PSMD13 |
| 11057\_EFF\_MEM\_VS\_CENT\_MEM\_CD4\_TCELL\_DN | 1.36 | 0.028 | 0.218 | PTK2 | | TSPAN14 | ATP1A1 |
| 22601\_IMMATURE\_CD4\_SINGLE\_POSITIVE\_VS\_DOUBLE\_POSITIVE\_THYMOCYTE\_UP | 1.39 | 0.037 | 0.247 | FETUB | | DSG3 | ENPP6 |
| 3982\_BCELL\_VS\_TH1\_UP | 1.33 | 0.046 | 0.259 | SYK | | MUC5AC | FETUB |
| 13411\_NAIVE\_VS\_IGM\_MEMORY\_BCELL\_DN | 1.43 | 0.026 | 0.287 | TSPAN14 | | SYK | UQCRC1 |
| 40274\_CTRL\_VS\_LEF1\_TRANSDUCED\_ACTIVATED\_CD4\_TCELL\_DN | 1.40 | 0.027 | 0.306 | IPO7 | | ATP1A1 | UQCRC1 |
| Negative correlation | | | | | | | |
| 19401\_UNSTIM\_VS\_RETINOIC\_ACID\_AND\_PAM2CSK4\_STIM\_FOLLICULAR\_DC\_UP | -1.42 | 0.039 | 0.254 | RIDA | | ENTPD6 | HSP90B1 |
| 37301\_LYMPHOID\_PRIMED\_MPP\_VS\_RAG2\_KO\_NK\_CELL\_DN | -1.49 | 0.027 | 0.274 | MPO | | C8A | KRT5 |
| 43260\_BTLA\_POS\_VS\_NEG\_INTRATUMORAL\_CD8\_TCELL\_UP | -1.32 | 0.024 | 0.339 | LYZ | | ENTPD6 | HSP90B1 |
190006, Genes up-regulated in comparison of peripheral blood mononuclear cells (PBMC) from patients with type 1 diabetes at the time of the diagnosis versus those at 4 months later.
11057, Genes down-regulated in comparison of effector memory T cells versus central memory T cells from peripheral blood mononuclear cells.
22601, Genes up-regulated in immature CD4 single positive cells versus double positive thymocytes.
3982, Genes up-regulated in comparison of B cells versus Th1 cells.
13411, Genes down-regulated in comparison of naive B cells versus IgM-memory B cells.
40274,Genes down-regulated in CD4 T conv: control versus over-expression of LEF1
19401, Genes up-regulated in the in vitro follicular dendritic cells from peripheral lymph nodes: non-stimulated versus tretinoin and Pam2CSK4 (96h).
37301 Genes down-regulated in lymphoid primed multipotent progenitors versus RAG2 knockout NK cells.
43260, Genes up-regulated in tumor-infiltrating CD8 T cells: BTLA+ versus BTLA-
2noramlized enrichment score, positive NES or negative NES suggests that the genes in the set are upregulated or downregulated, respectively
3normalized p value
4false discovery rate q-value

## Slide 5
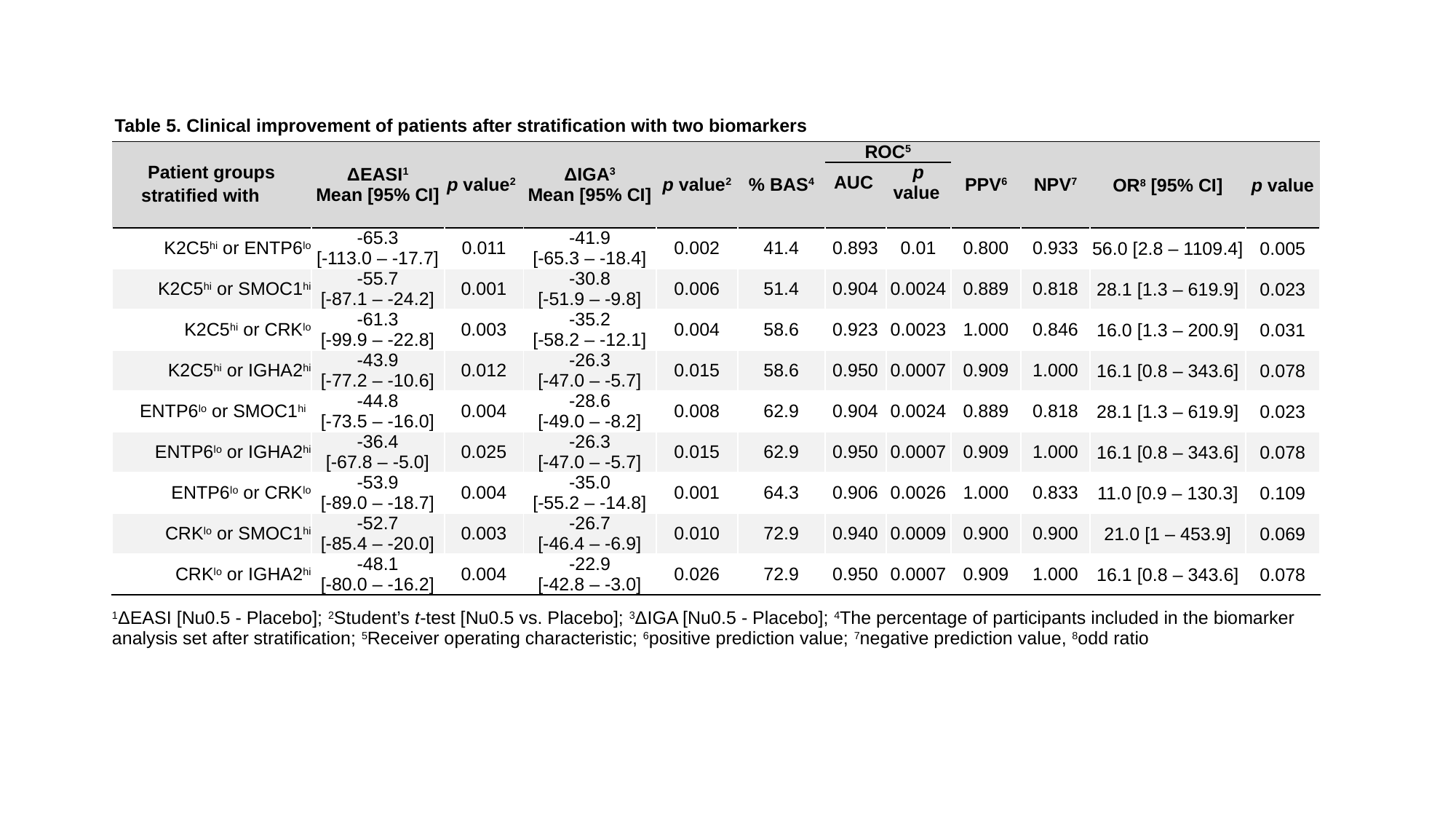

Table 5. Clinical improvement of patients after stratification with two biomarkers
| Patient groups stratified with | ΔEASI1 Mean [95% CI] | p value2 | ΔIGA3 Mean [95% CI] | p value2 | % BAS4 | ROC5 | | PPV6 | NPV7 | OR8 [95% CI] | p value |
| --- | --- | --- | --- | --- | --- | --- | --- | --- | --- | --- | --- |
| | | | | | | AUC | p value | | | | |
| K2C5hi or ENTP6lo | -65.3 [-113.0 – -17.7] | 0.011 | -41.9 [-65.3 – -18.4] | 0.002 | 41.4 | 0.893 | 0.01 | 0.800 | 0.933 | 56.0 [2.8 – 1109.4] | 0.005 |
| K2C5hi or SMOC1hi | -55.7 [-87.1 – -24.2] | 0.001 | -30.8 [-51.9 – -9.8] | 0.006 | 51.4 | 0.904 | 0.0024 | 0.889 | 0.818 | 28.1 [1.3 – 619.9] | 0.023 |
| K2C5hi or CRKlo | -61.3 [-99.9 – -22.8] | 0.003 | -35.2 [-58.2 – -12.1] | 0.004 | 58.6 | 0.923 | 0.0023 | 1.000 | 0.846 | 16.0 [1.3 – 200.9] | 0.031 |
| K2C5hi or IGHA2hi | -43.9 [-77.2 – -10.6] | 0.012 | -26.3 [-47.0 – -5.7] | 0.015 | 58.6 | 0.950 | 0.0007 | 0.909 | 1.000 | 16.1 [0.8 – 343.6] | 0.078 |
| ENTP6lo or SMOC1hi | -44.8 [-73.5 – -16.0] | 0.004 | -28.6 [-49.0 – -8.2] | 0.008 | 62.9 | 0.904 | 0.0024 | 0.889 | 0.818 | 28.1 [1.3 – 619.9] | 0.023 |
| ENTP6lo or IGHA2hi | -36.4 [-67.8 – -5.0] | 0.025 | -26.3 [-47.0 – -5.7] | 0.015 | 62.9 | 0.950 | 0.0007 | 0.909 | 1.000 | 16.1 [0.8 – 343.6] | 0.078 |
| ENTP6lo or CRKlo | -53.9 [-89.0 – -18.7] | 0.004 | -35.0 [-55.2 – -14.8] | 0.001 | 64.3 | 0.906 | 0.0026 | 1.000 | 0.833 | 11.0 [0.9 – 130.3] | 0.109 |
| CRKlo or SMOC1hi | -52.7 [-85.4 – -20.0] | 0.003 | -26.7 [-46.4 – -6.9] | 0.010 | 72.9 | 0.940 | 0.0009 | 0.900 | 0.900 | 21.0 [1 – 453.9] | 0.069 |
| CRKlo or IGHA2hi | -48.1 [-80.0 – -16.2] | 0.004 | -22.9 [-42.8 – -3.0] | 0.026 | 72.9 | 0.950 | 0.0007 | 0.909 | 1.000 | 16.1 [0.8 – 343.6] | 0.078 |
| 1ΔEASI [Nu0.5 - Placebo]; 2Student’s t-test [Nu0.5 vs. Placebo]; 3ΔIGA [Nu0.5 - Placebo]; 4The percentage of participants included in the biomarker analysis set after stratification; 5Receiver operating characteristic; 6positive prediction value; 7negative prediction value, 8odd ratio |
| --- |

## Slide 6
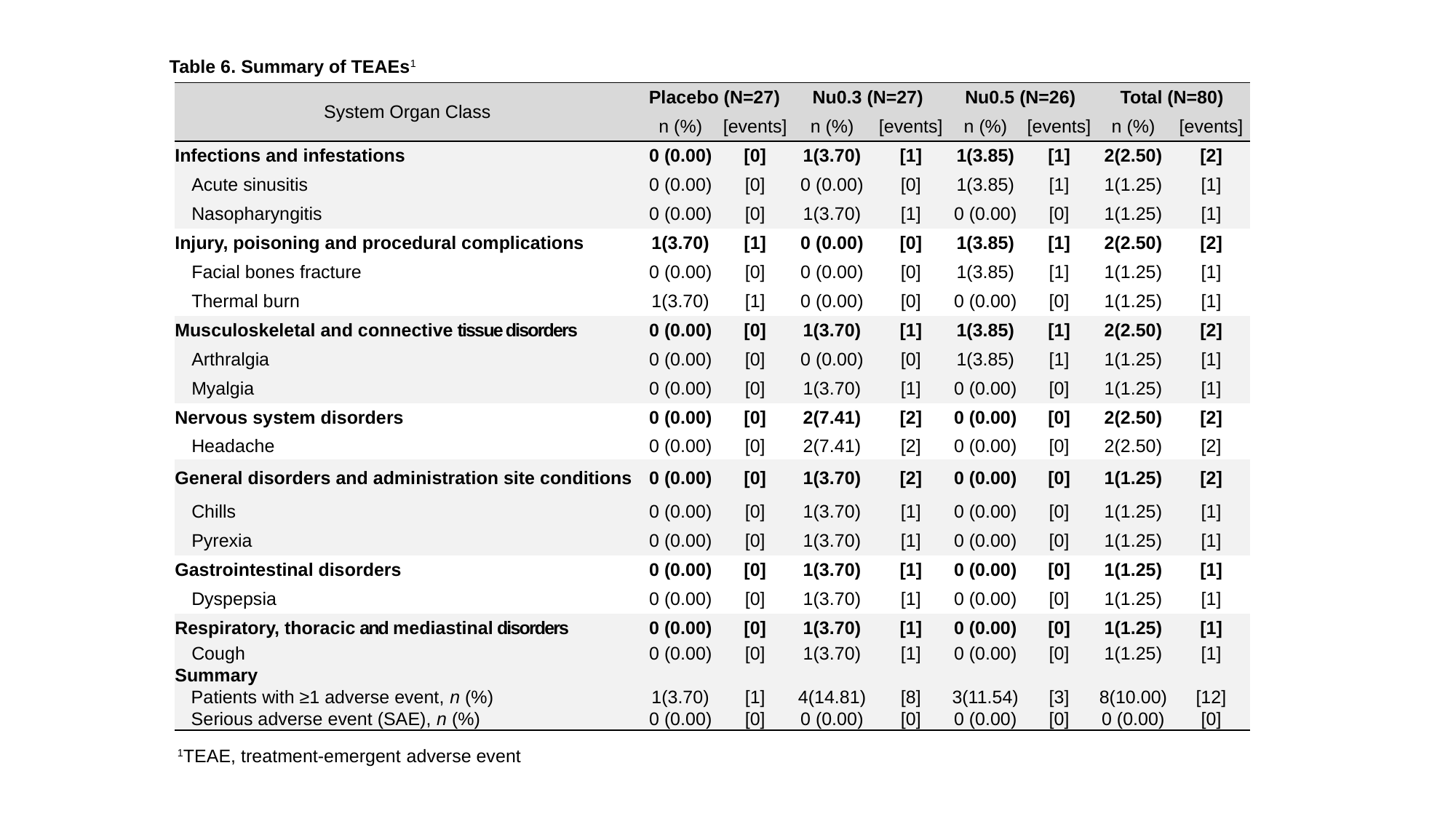

Table 6. Summary of TEAEs1
| System Organ Class | Placebo (N=27) | | Nu0.3 (N=27) | | Nu0.5 (N=26) | | Total (N=80) | |
| --- | --- | --- | --- | --- | --- | --- | --- | --- |
| | n (%) | [events] | n (%) | [events] | n (%) | [events] | n (%) | [events] |
| Infections and infestations | 0 (0.00) | [0] | 1(3.70) | [1] | 1(3.85) | [1] | 2(2.50) | [2] |
| Acute sinusitis | 0 (0.00) | [0] | 0 (0.00) | [0] | 1(3.85) | [1] | 1(1.25) | [1] |
| Nasopharyngitis | 0 (0.00) | [0] | 1(3.70) | [1] | 0 (0.00) | [0] | 1(1.25) | [1] |
| Injury, poisoning and procedural complications | 1(3.70) | [1] | 0 (0.00) | [0] | 1(3.85) | [1] | 2(2.50) | [2] |
| Facial bones fracture | 0 (0.00) | [0] | 0 (0.00) | [0] | 1(3.85) | [1] | 1(1.25) | [1] |
| Thermal burn | 1(3.70) | [1] | 0 (0.00) | [0] | 0 (0.00) | [0] | 1(1.25) | [1] |
| Musculoskeletal and connective tissue disorders | 0 (0.00) | [0] | 1(3.70) | [1] | 1(3.85) | [1] | 2(2.50) | [2] |
| Arthralgia | 0 (0.00) | [0] | 0 (0.00) | [0] | 1(3.85) | [1] | 1(1.25) | [1] |
| Myalgia | 0 (0.00) | [0] | 1(3.70) | [1] | 0 (0.00) | [0] | 1(1.25) | [1] |
| Nervous system disorders | 0 (0.00) | [0] | 2(7.41) | [2] | 0 (0.00) | [0] | 2(2.50) | [2] |
| Headache | 0 (0.00) | [0] | 2(7.41) | [2] | 0 (0.00) | [0] | 2(2.50) | [2] |
| General disorders and administration site conditions | 0 (0.00) | [0] | 1(3.70) | [2] | 0 (0.00) | [0] | 1(1.25) | [2] |
| Chills | 0 (0.00) | [0] | 1(3.70) | [1] | 0 (0.00) | [0] | 1(1.25) | [1] |
| Pyrexia | 0 (0.00) | [0] | 1(3.70) | [1] | 0 (0.00) | [0] | 1(1.25) | [1] |
| Gastrointestinal disorders | 0 (0.00) | [0] | 1(3.70) | [1] | 0 (0.00) | [0] | 1(1.25) | [1] |
| Dyspepsia | 0 (0.00) | [0] | 1(3.70) | [1] | 0 (0.00) | [0] | 1(1.25) | [1] |
| Respiratory, thoracic and mediastinal disorders | 0 (0.00) | [0] | 1(3.70) | [1] | 0 (0.00) | [0] | 1(1.25) | [1] |
| Cough | 0 (0.00) | [0] | 1(3.70) | [1] | 0 (0.00) | [0] | 1(1.25) | [1] |
| Summary | | | | | | | | |
| Patients with ≥1 adverse event, n (%) | 1(3.70) | [1] | 4(14.81) | [8] | 3(11.54) | [3] | 8(10.00) | [12] |
| Serious adverse event (SAE), n (%) | 0 (0.00) | [0] | 0 (0.00) | [0] | 0 (0.00) | [0] | 0 (0.00) | [0] |
1TEAE, treatment-emergent adverse event

## Slide 7
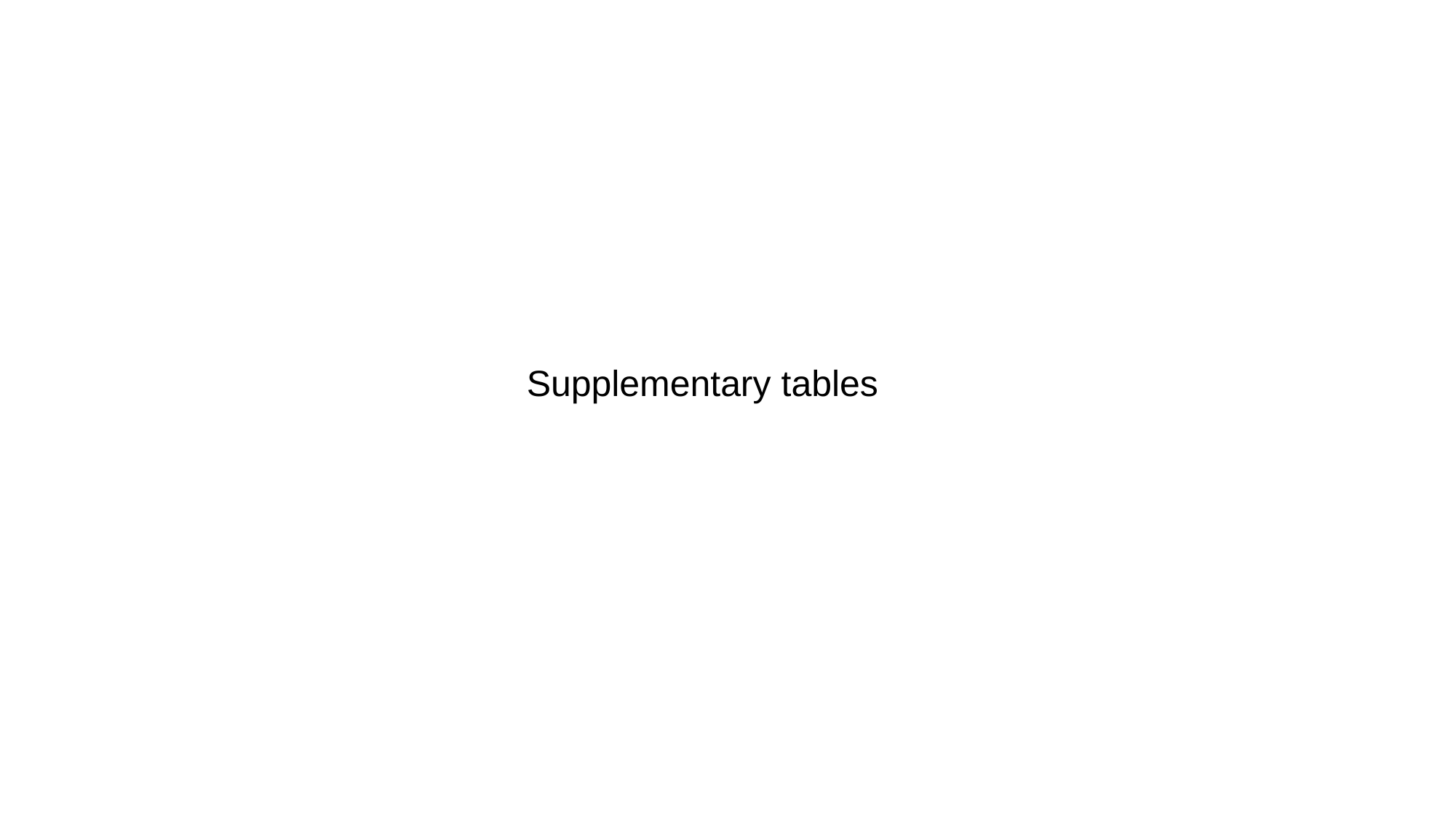

Supplementary tables

## Slide 8
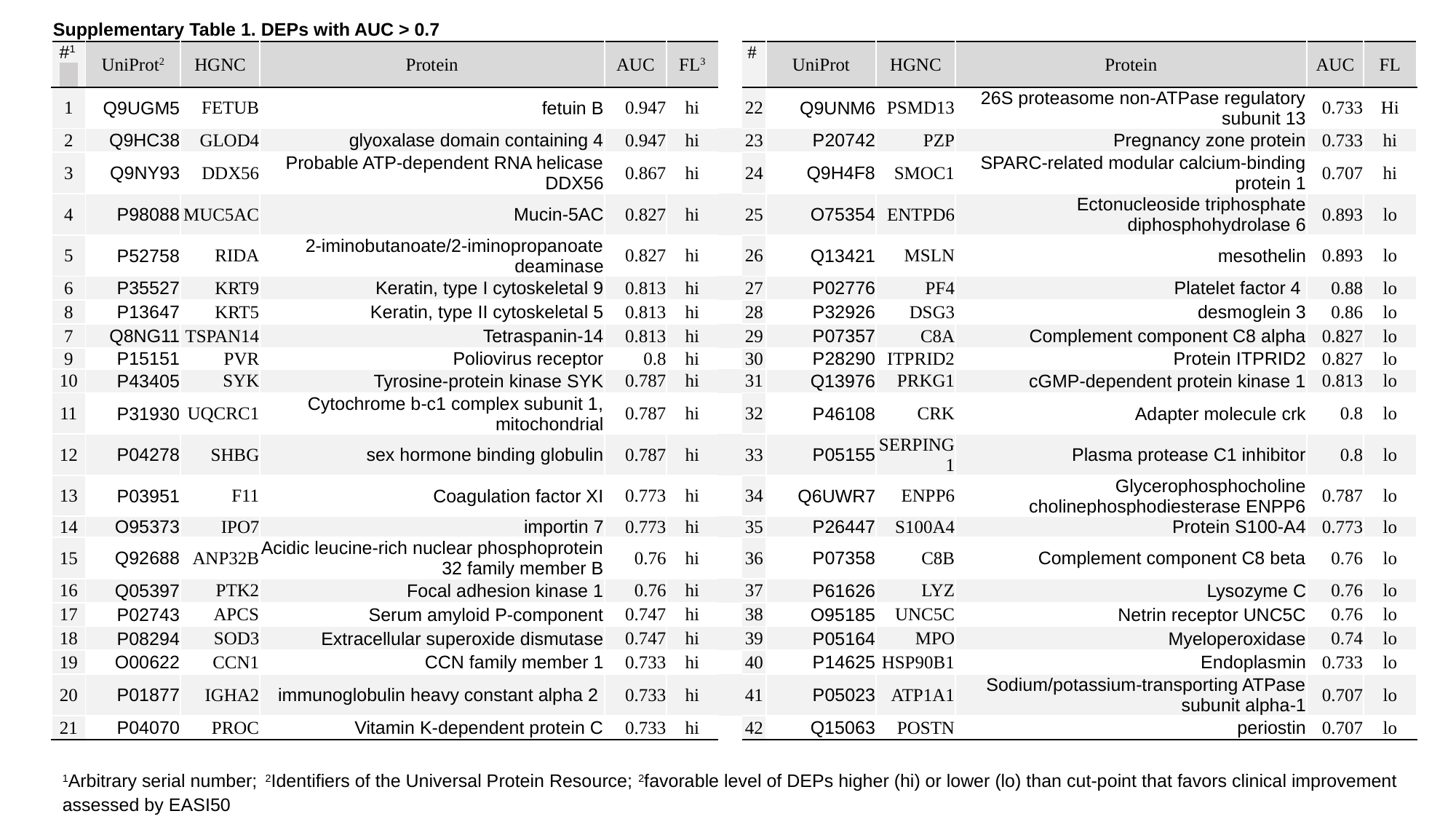

Supplementary Table 1. DEPs with AUC > 0.7
| #1 | UniProt2 | HGNC | Protein | AUC | FL3 | | # | UniProt | HGNC | Protein | AUC | FL |
| --- | --- | --- | --- | --- | --- | --- | --- | --- | --- | --- | --- | --- |
| 1 | Q9UGM5 | FETUB | fetuin B | 0.947 | hi | | 22 | Q9UNM6 | PSMD13 | 26S proteasome non-ATPase regulatory subunit 13 | 0.733 | Hi |
| 2 | Q9HC38 | GLOD4 | glyoxalase domain containing 4 | 0.947 | hi | | 23 | P20742 | PZP | Pregnancy zone protein | 0.733 | hi |
| 3 | Q9NY93 | DDX56 | Probable ATP-dependent RNA helicase DDX56 | 0.867 | hi | | 24 | Q9H4F8 | SMOC1 | SPARC-related modular calcium-binding protein 1 | 0.707 | hi |
| 4 | P98088 | MUC5AC | Mucin-5AC | 0.827 | hi | | 25 | O75354 | ENTPD6 | Ectonucleoside triphosphate diphosphohydrolase 6 | 0.893 | lo |
| 5 | P52758 | RIDA | 2-iminobutanoate/2-iminopropanoate deaminase | 0.827 | hi | | 26 | Q13421 | MSLN | mesothelin | 0.893 | lo |
| 6 | P35527 | KRT9 | Keratin, type I cytoskeletal 9 | 0.813 | hi | | 27 | P02776 | PF4 | Platelet factor 4 | 0.88 | lo |
| 8 | P13647 | KRT5 | Keratin, type II cytoskeletal 5 | 0.813 | hi | | 28 | P32926 | DSG3 | desmoglein 3 | 0.86 | lo |
| 7 | Q8NG11 | TSPAN14 | Tetraspanin-14 | 0.813 | hi | | 29 | P07357 | C8A | Complement component C8 alpha | 0.827 | lo |
| 9 | P15151 | PVR | Poliovirus receptor | 0.8 | hi | | 30 | P28290 | ITPRID2 | Protein ITPRID2 | 0.827 | lo |
| 10 | P43405 | SYK | Tyrosine-protein kinase SYK | 0.787 | hi | | 31 | Q13976 | PRKG1 | cGMP-dependent protein kinase 1 | 0.813 | lo |
| 11 | P31930 | UQCRC1 | Cytochrome b-c1 complex subunit 1, mitochondrial | 0.787 | hi | | 32 | P46108 | CRK | Adapter molecule crk | 0.8 | lo |
| 12 | P04278 | SHBG | sex hormone binding globulin | 0.787 | hi | | 33 | P05155 | SERPING1 | Plasma protease C1 inhibitor | 0.8 | lo |
| 13 | P03951 | F11 | Coagulation factor XI | 0.773 | hi | | 34 | Q6UWR7 | ENPP6 | Glycerophosphocholine cholinephosphodiesterase ENPP6 | 0.787 | lo |
| 14 | O95373 | IPO7 | importin 7 | 0.773 | hi | | 35 | P26447 | S100A4 | Protein S100-A4 | 0.773 | lo |
| 15 | Q92688 | ANP32B | Acidic leucine-rich nuclear phosphoprotein 32 family member B | 0.76 | hi | | 36 | P07358 | C8B | Complement component C8 beta | 0.76 | lo |
| 16 | Q05397 | PTK2 | Focal adhesion kinase 1 | 0.76 | hi | | 37 | P61626 | LYZ | Lysozyme C | 0.76 | lo |
| 17 | P02743 | APCS | Serum amyloid P-component | 0.747 | hi | | 38 | O95185 | UNC5C | Netrin receptor UNC5C | 0.76 | lo |
| 18 | P08294 | SOD3 | Extracellular superoxide dismutase | 0.747 | hi | | 39 | P05164 | MPO | Myeloperoxidase | 0.74 | lo |
| 19 | O00622 | CCN1 | CCN family member 1 | 0.733 | hi | | 40 | P14625 | HSP90B1 | Endoplasmin | 0.733 | lo |
| 20 | P01877 | IGHA2 | immunoglobulin heavy constant alpha 2 | 0.733 | hi | | 41 | P05023 | ATP1A1 | Sodium/potassium-transporting ATPase subunit alpha-1 | 0.707 | lo |
| 21 | P04070 | PROC | Vitamin K-dependent protein C | 0.733 | hi | | 42 | Q15063 | POSTN | periostin | 0.707 | lo |
1Arbitrary serial number; 2Identifiers of the Universal Protein Resource; 2favorable level of DEPs higher (hi) or lower (lo) than cut-point that favors clinical improvement assessed by EASI50

## Slide 9
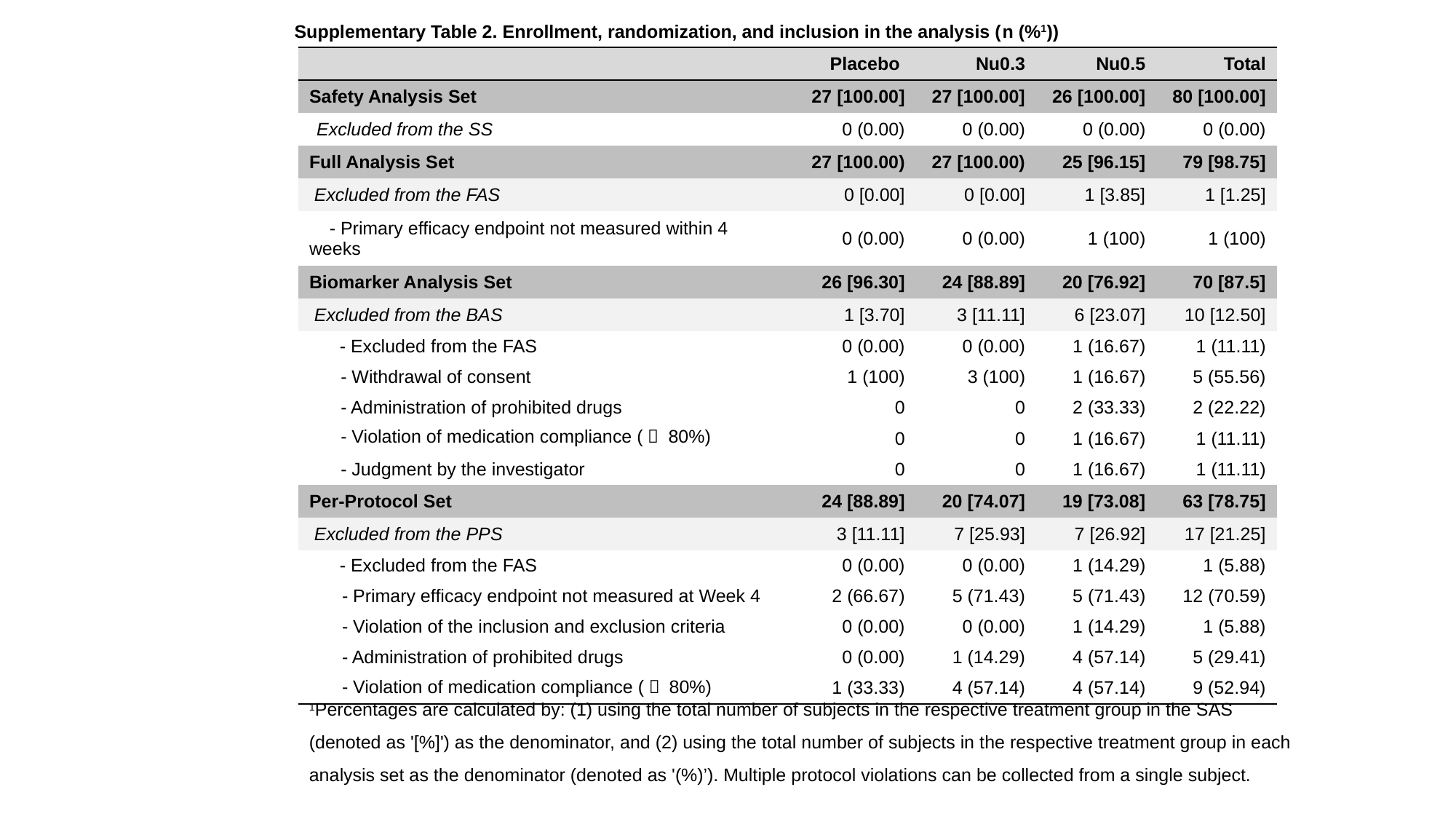

Supplementary Table 2. Enrollment, randomization, and inclusion in the analysis (n (%1))
| | Placebo | Nu0.3 | Nu0.5 | Total |
| --- | --- | --- | --- | --- |
| Safety Analysis Set | 27 [100.00] | 27 [100.00] | 26 [100.00] | 80 [100.00] |
| Excluded from the SS | 0 (0.00) | 0 (0.00) | 0 (0.00) | 0 (0.00) |
| Full Analysis Set | 27 [100.00) | 27 [100.00) | 25 [96.15] | 79 [98.75] |
| Excluded from the FAS | 0 [0.00] | 0 [0.00] | 1 [3.85] | 1 [1.25] |
| - Primary efficacy endpoint not measured within 4 weeks | 0 (0.00) | 0 (0.00) | 1 (100) | 1 (100) |
| Biomarker Analysis Set | 26 [96.30] | 24 [88.89] | 20 [76.92] | 70 [87.5] |
| Excluded from the BAS | 1 [3.70] | 3 [11.11] | 6 [23.07] | 10 [12.50] |
| - Excluded from the FAS | 0 (0.00) | 0 (0.00) | 1 (16.67) | 1 (11.11) |
| - Withdrawal of consent | 1 (100) | 3 (100) | 1 (16.67) | 5 (55.56) |
| - Administration of prohibited drugs | 0 | 0 | 2 (33.33) | 2 (22.22) |
| - Violation of medication compliance (＜ 80%) | 0 | 0 | 1 (16.67) | 1 (11.11) |
| - Judgment by the investigator | 0 | 0 | 1 (16.67) | 1 (11.11) |
| Per-Protocol Set | 24 [88.89] | 20 [74.07] | 19 [73.08] | 63 [78.75] |
| Excluded from the PPS | 3 [11.11] | 7 [25.93] | 7 [26.92] | 17 [21.25] |
| - Excluded from the FAS | 0 (0.00) | 0 (0.00) | 1 (14.29) | 1 (5.88) |
| - Primary efficacy endpoint not measured at Week 4 | 2 (66.67) | 5 (71.43) | 5 (71.43) | 12 (70.59) |
| - Violation of the inclusion and exclusion criteria | 0 (0.00) | 0 (0.00) | 1 (14.29) | 1 (5.88) |
| - Administration of prohibited drugs | 0 (0.00) | 1 (14.29) | 4 (57.14) | 5 (29.41) |
| - Violation of medication compliance (＜ 80%) | 1 (33.33) | 4 (57.14) | 4 (57.14) | 9 (52.94) |
1Percentages are calculated by: (1) using the total number of subjects in the respective treatment group in the SAS (denoted as '[%]') as the denominator, and (2) using the total number of subjects in the respective treatment group in each analysis set as the denominator (denoted as '(%)’). Multiple protocol violations can be collected from a single subject.

## Slide 10
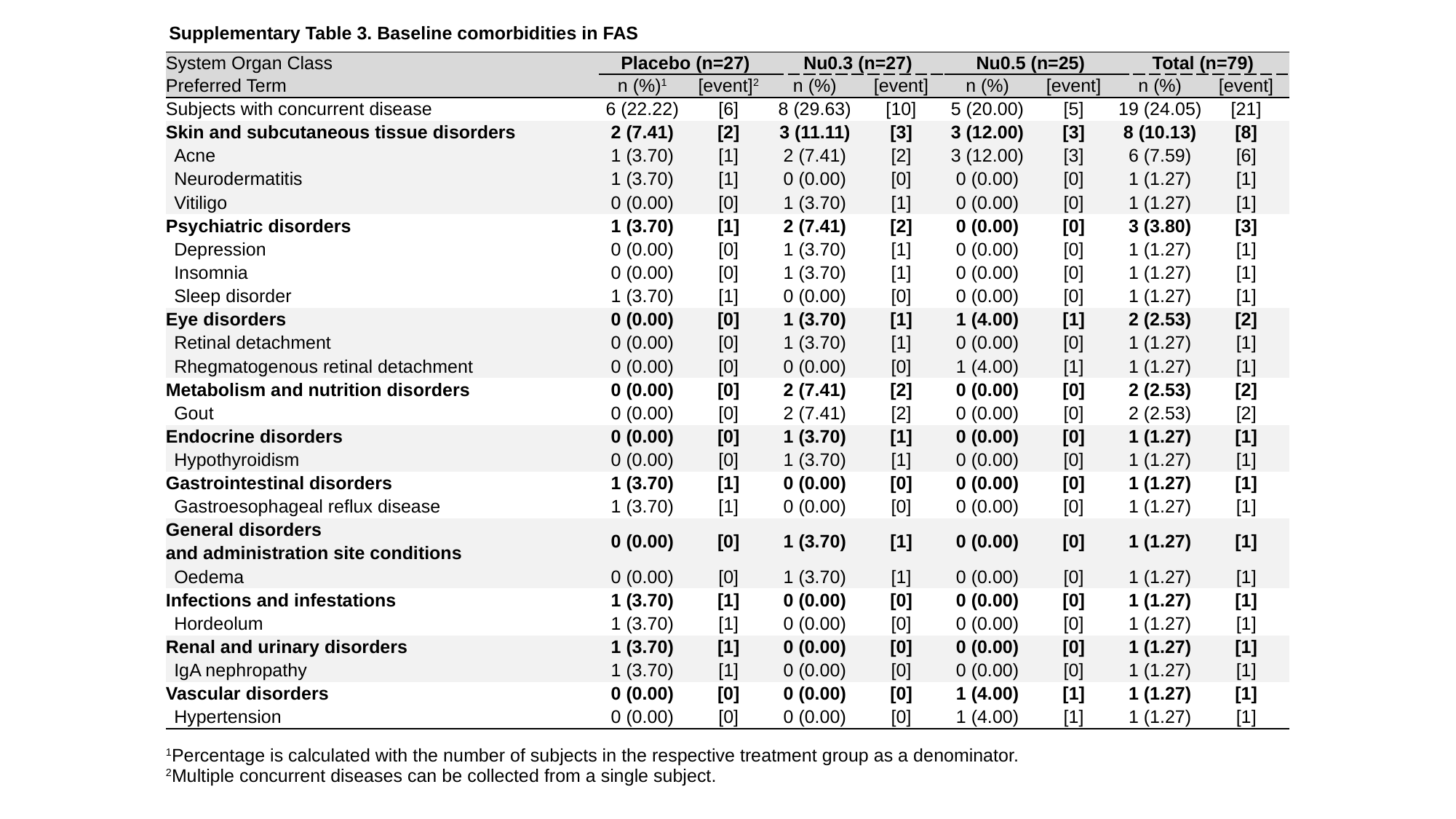

Supplementary Table 3. Baseline comorbidities in FAS
| System Organ Class | Placebo (n=27) | | Nu0.3 (n=27) | | Nu0.5 (n=25) | | Total (n=79) | |
| --- | --- | --- | --- | --- | --- | --- | --- | --- |
| Preferred Term | n (%)1 | [event]2 | n (%) | [event] | n (%) | [event] | n (%) | [event] |
| Subjects with concurrent disease | 6 (22.22) | [6] | 8 (29.63) | [10] | 5 (20.00) | [5] | 19 (24.05) | [21] |
| Skin and subcutaneous tissue disorders | 2 (7.41) | [2] | 3 (11.11) | [3] | 3 (12.00) | [3] | 8 (10.13) | [8] |
| Acne | 1 (3.70) | [1] | 2 (7.41) | [2] | 3 (12.00) | [3] | 6 (7.59) | [6] |
| Neurodermatitis | 1 (3.70) | [1] | 0 (0.00) | [0] | 0 (0.00) | [0] | 1 (1.27) | [1] |
| Vitiligo | 0 (0.00) | [0] | 1 (3.70) | [1] | 0 (0.00) | [0] | 1 (1.27) | [1] |
| Psychiatric disorders | 1 (3.70) | [1] | 2 (7.41) | [2] | 0 (0.00) | [0] | 3 (3.80) | [3] |
| Depression | 0 (0.00) | [0] | 1 (3.70) | [1] | 0 (0.00) | [0] | 1 (1.27) | [1] |
| Insomnia | 0 (0.00) | [0] | 1 (3.70) | [1] | 0 (0.00) | [0] | 1 (1.27) | [1] |
| Sleep disorder | 1 (3.70) | [1] | 0 (0.00) | [0] | 0 (0.00) | [0] | 1 (1.27) | [1] |
| Eye disorders | 0 (0.00) | [0] | 1 (3.70) | [1] | 1 (4.00) | [1] | 2 (2.53) | [2] |
| Retinal detachment | 0 (0.00) | [0] | 1 (3.70) | [1] | 0 (0.00) | [0] | 1 (1.27) | [1] |
| Rhegmatogenous retinal detachment | 0 (0.00) | [0] | 0 (0.00) | [0] | 1 (4.00) | [1] | 1 (1.27) | [1] |
| Metabolism and nutrition disorders | 0 (0.00) | [0] | 2 (7.41) | [2] | 0 (0.00) | [0] | 2 (2.53) | [2] |
| Gout | 0 (0.00) | [0] | 2 (7.41) | [2] | 0 (0.00) | [0] | 2 (2.53) | [2] |
| Endocrine disorders | 0 (0.00) | [0] | 1 (3.70) | [1] | 0 (0.00) | [0] | 1 (1.27) | [1] |
| Hypothyroidism | 0 (0.00) | [0] | 1 (3.70) | [1] | 0 (0.00) | [0] | 1 (1.27) | [1] |
| Gastrointestinal disorders | 1 (3.70) | [1] | 0 (0.00) | [0] | 0 (0.00) | [0] | 1 (1.27) | [1] |
| Gastroesophageal reflux disease | 1 (3.70) | [1] | 0 (0.00) | [0] | 0 (0.00) | [0] | 1 (1.27) | [1] |
| General disorders and administration site conditions | 0 (0.00) | [0] | 1 (3.70) | [1] | 0 (0.00) | [0] | 1 (1.27) | [1] |
| Oedema | 0 (0.00) | [0] | 1 (3.70) | [1] | 0 (0.00) | [0] | 1 (1.27) | [1] |
| Infections and infestations | 1 (3.70) | [1] | 0 (0.00) | [0] | 0 (0.00) | [0] | 1 (1.27) | [1] |
| Hordeolum | 1 (3.70) | [1] | 0 (0.00) | [0] | 0 (0.00) | [0] | 1 (1.27) | [1] |
| Renal and urinary disorders | 1 (3.70) | [1] | 0 (0.00) | [0] | 0 (0.00) | [0] | 1 (1.27) | [1] |
| IgA nephropathy | 1 (3.70) | [1] | 0 (0.00) | [0] | 0 (0.00) | [0] | 1 (1.27) | [1] |
| Vascular disorders | 0 (0.00) | [0] | 0 (0.00) | [0] | 1 (4.00) | [1] | 1 (1.27) | [1] |
| Hypertension | 0 (0.00) | [0] | 0 (0.00) | [0] | 1 (4.00) | [1] | 1 (1.27) | [1] |
| 1Percentage is calculated with the number of subjects in the respective treatment group as a denominator. 2Multiple concurrent diseases can be collected from a single subject. | | | | | | | | |

## Slide 11
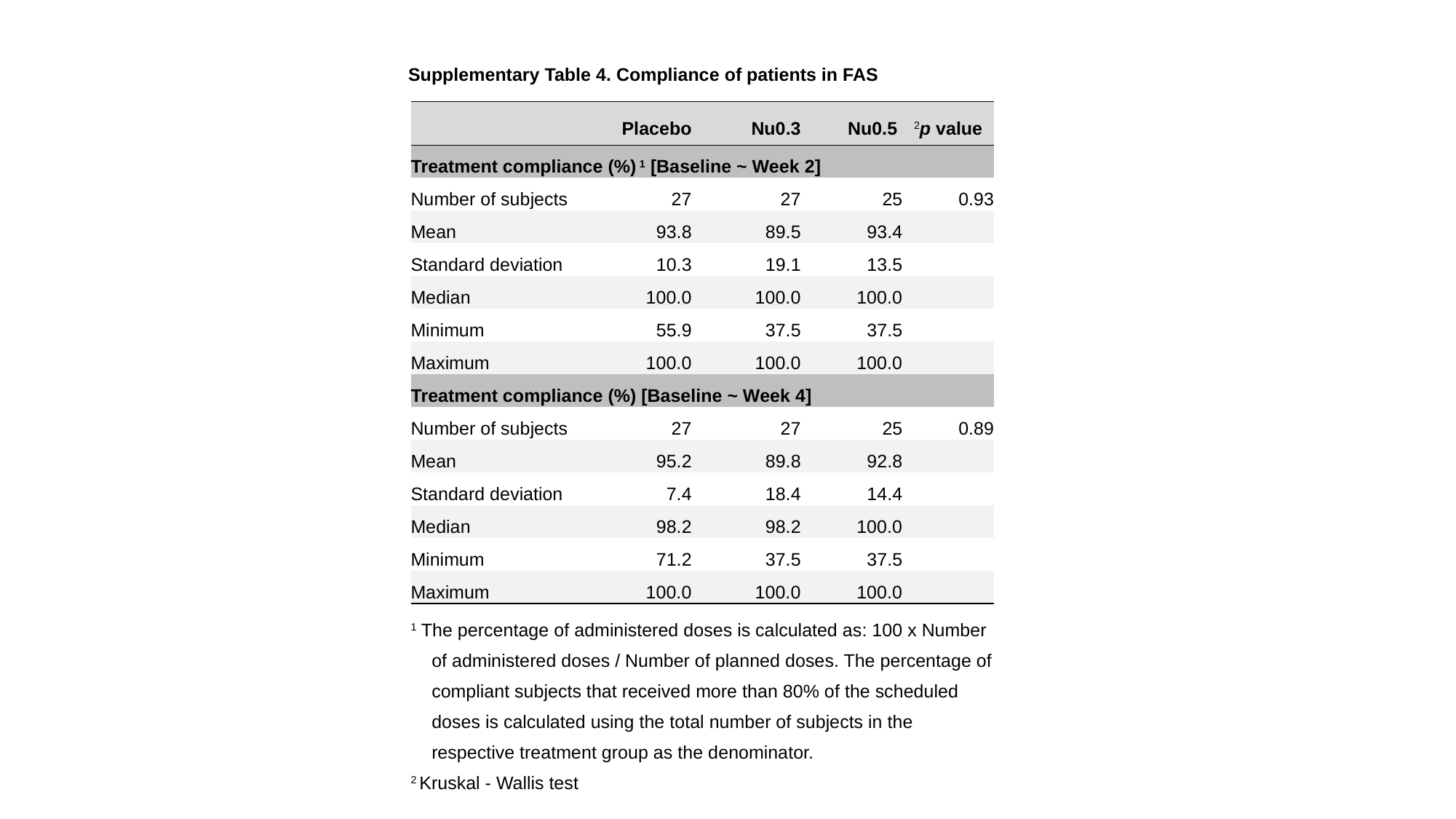

Supplementary Table 4. Compliance of patients in FAS
| | Placebo | Nu0.3 | Nu0.5 | 2p value |
| --- | --- | --- | --- | --- |
| Treatment compliance (%) 1 [Baseline ~ Week 2] | | | | |
| Number of subjects | 27 | 27 | 25 | 0.93 |
| Mean | 93.8 | 89.5 | 93.4 | |
| Standard deviation | 10.3 | 19.1 | 13.5 | |
| Median | 100.0 | 100.0 | 100.0 | |
| Minimum | 55.9 | 37.5 | 37.5 | |
| Maximum | 100.0 | 100.0 | 100.0 | |
| Treatment compliance (%) [Baseline ~ Week 4] | | | | |
| Number of subjects | 27 | 27 | 25 | 0.89 |
| Mean | 95.2 | 89.8 | 92.8 | |
| Standard deviation | 7.4 | 18.4 | 14.4 | |
| Median | 98.2 | 98.2 | 100.0 | |
| Minimum | 71.2 | 37.5 | 37.5 | |
| Maximum | 100.0 | 100.0 | 100.0 | |
| 1 The percentage of administered doses is calculated as: 100 x Number of administered doses / Number of planned doses. The percentage of compliant subjects that received more than 80% of the scheduled doses is calculated using the total number of subjects in the respective treatment group as the denominator. 2 Kruskal - Wallis test | | | | |

## Slide 12
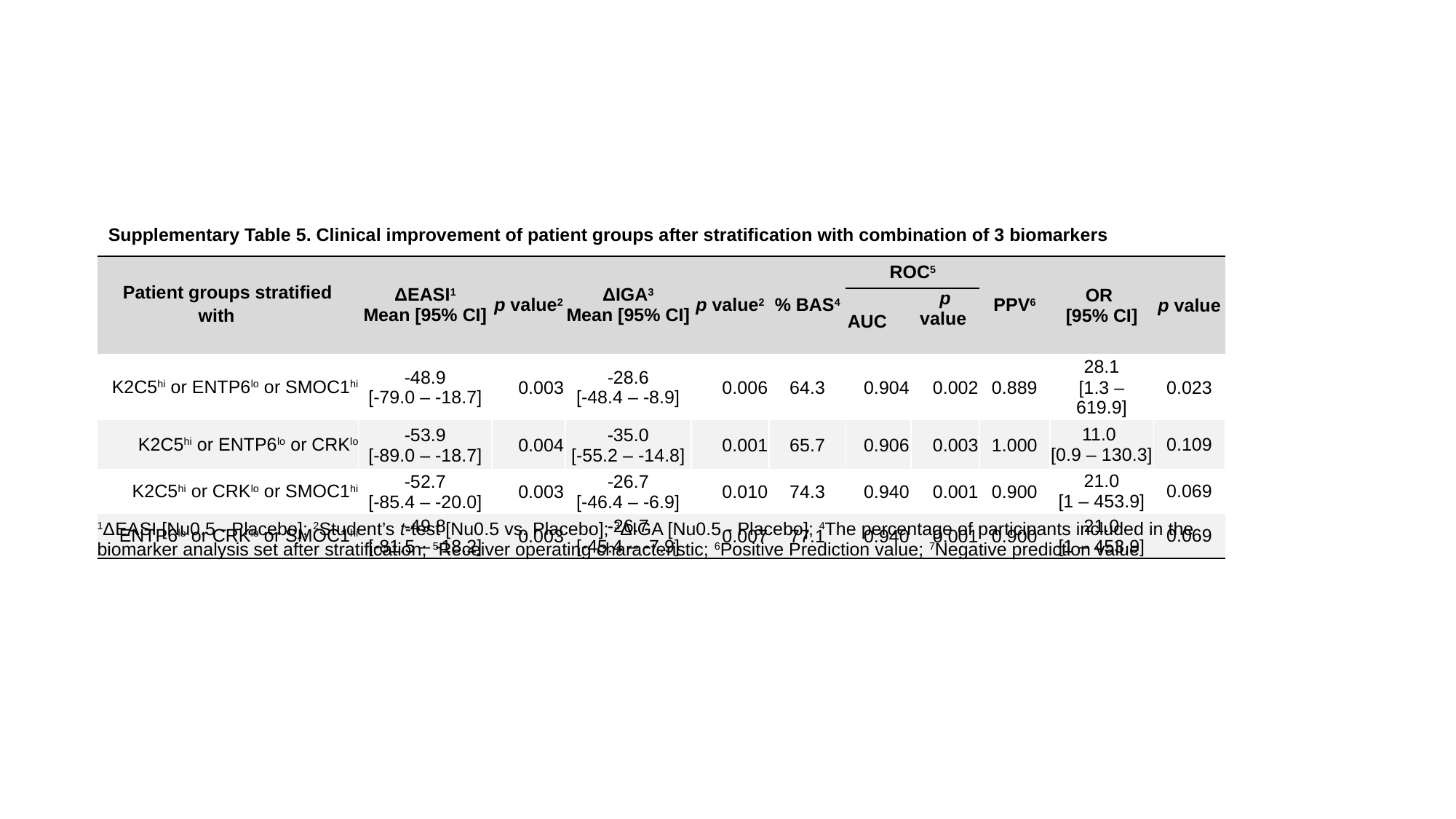

Supplementary Table 5. Clinical improvement of patient groups after stratification with combination of 3 biomarkers
| Patient groups stratified with | ΔEASI1 Mean [95% CI] | p value2 | ΔIGA3 Mean [95% CI] | p value2 | % BAS4 | ROC5 | | PPV6 | OR [95% CI] | p value |
| --- | --- | --- | --- | --- | --- | --- | --- | --- | --- | --- |
| | | | | | | AUC | p value | | | |
| K2C5hi or ENTP6lo or SMOC1hi | -48.9 [-79.0 – -18.7] | 0.003 | -28.6 [-48.4 – -8.9] | 0.006 | 64.3 | 0.904 | 0.002 | 0.889 | 28.1 [1.3 – 619.9] | 0.023 |
| K2C5hi or ENTP6lo or CRKlo | -53.9 [-89.0 – -18.7] | 0.004 | -35.0 [-55.2 – -14.8] | 0.001 | 65.7 | 0.906 | 0.003 | 1.000 | 11.0 [0.9 – 130.3] | 0.109 |
| K2C5hi or CRKlo or SMOC1hi | -52.7 [-85.4 – -20.0] | 0.003 | -26.7 [-46.4 – -6.9] | 0.010 | 74.3 | 0.940 | 0.001 | 0.900 | 21.0 [1 – 453.9] | 0.069 |
| ENTP6lo or CRKlo or SMOC1hi | -49.8 [-81.5 – -18.2] | 0.003 | -26.7 [-45.4 – -7.9] | 0.007 | 77.1 | 0.940 | 0.001 | 0.900 | 21.0 [1 – 453.9] | 0.069 |
| 1ΔEASI [Nu0.5 - Placebo]; 2Student’s t-test [Nu0.5 vs. Placebo]; 3ΔIGA [Nu0.5 - Placebo]; 4The percentage of participants included in the biomarker analysis set after stratification; 5Receiver operating characteristic; 6Positive Prediction value; 7Negative prediction value |
| --- |

## Slide 13
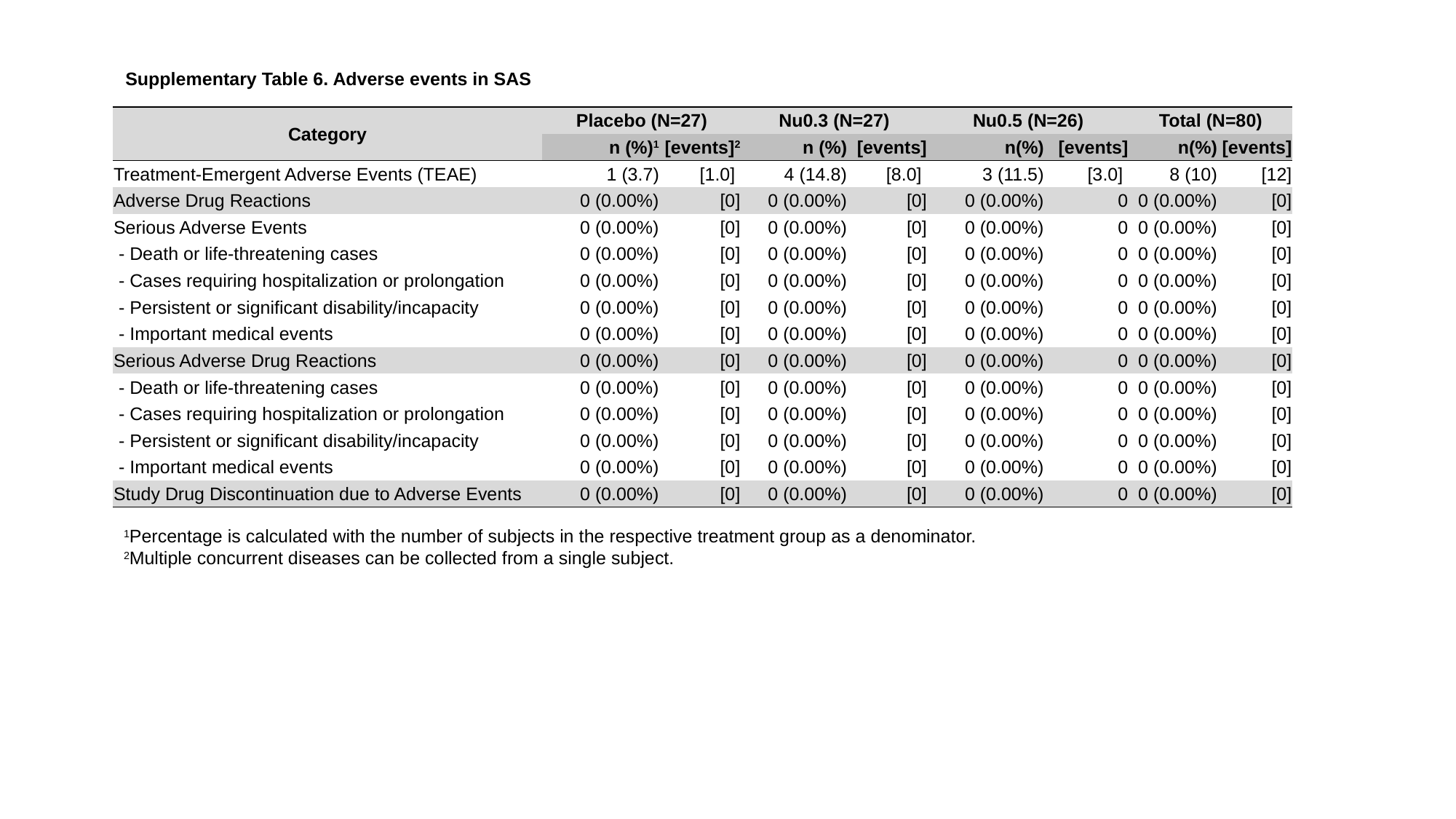

Supplementary Table 6. Adverse events in SAS
| Category | Placebo (N=27) | | Nu0.3 (N=27) | | Nu0.5 (N=26) | | Total (N=80) | |
| --- | --- | --- | --- | --- | --- | --- | --- | --- |
| | n (%)1 | [events]2 | n (%) | [events] | n(%) | [events] | n(%) | [events] |
| Treatment-Emergent Adverse Events (TEAE) | 1 (3.7) | [1.0] | 4 (14.8) | [8.0] | 3 (11.5) | [3.0] | 8 (10) | [12] |
| Adverse Drug Reactions | 0 (0.00%) | [0] | 0 (0.00%) | [0] | 0 (0.00%) | 0 | 0 (0.00%) | [0] |
| Serious Adverse Events | 0 (0.00%) | [0] | 0 (0.00%) | [0] | 0 (0.00%) | 0 | 0 (0.00%) | [0] |
| - Death or life-threatening cases | 0 (0.00%) | [0] | 0 (0.00%) | [0] | 0 (0.00%) | 0 | 0 (0.00%) | [0] |
| - Cases requiring hospitalization or prolongation | 0 (0.00%) | [0] | 0 (0.00%) | [0] | 0 (0.00%) | 0 | 0 (0.00%) | [0] |
| - Persistent or significant disability/incapacity | 0 (0.00%) | [0] | 0 (0.00%) | [0] | 0 (0.00%) | 0 | 0 (0.00%) | [0] |
| - Important medical events | 0 (0.00%) | [0] | 0 (0.00%) | [0] | 0 (0.00%) | 0 | 0 (0.00%) | [0] |
| Serious Adverse Drug Reactions | 0 (0.00%) | [0] | 0 (0.00%) | [0] | 0 (0.00%) | 0 | 0 (0.00%) | [0] |
| - Death or life-threatening cases | 0 (0.00%) | [0] | 0 (0.00%) | [0] | 0 (0.00%) | 0 | 0 (0.00%) | [0] |
| - Cases requiring hospitalization or prolongation | 0 (0.00%) | [0] | 0 (0.00%) | [0] | 0 (0.00%) | 0 | 0 (0.00%) | [0] |
| - Persistent or significant disability/incapacity | 0 (0.00%) | [0] | 0 (0.00%) | [0] | 0 (0.00%) | 0 | 0 (0.00%) | [0] |
| - Important medical events | 0 (0.00%) | [0] | 0 (0.00%) | [0] | 0 (0.00%) | 0 | 0 (0.00%) | [0] |
| Study Drug Discontinuation due to Adverse Events | 0 (0.00%) | [0] | 0 (0.00%) | [0] | 0 (0.00%) | 0 | 0 (0.00%) | [0] |
1Percentage is calculated with the number of subjects in the respective treatment group as a denominator.
2Multiple concurrent diseases can be collected from a single subject.
