# Supplementary material for: A phase 2a double-blind, placebo-controlled, randomized clinical trial evaluating the efficacy and safety of NuGel, a novel topical GPCR19-mediated inflammasome inhibitor, in patients with mild to moderate atopic dermatitis: a proof-of-concept study with Post-hoc biomarker analysis
Source: Front Immunol. 2025 May 19;16:1560447. doi: 10.3389/fimmu.2025.1560447 (PMC12127193; doi:10.3389/fimmu.2025.1560447)
Supplement: Supplementary file 5 [file DataSheet2.pdf]

Frontiers in Immunology,
Section: Autoimmune and Autoinflammatory Disorders: Autoinflammatory Disorders
Research Topic: Immunoregulation and treatment of allergic diseases

Title of Manuscript: "A Phase 2a Placebo-Controlled Randomized Study of a Topical Inflammasome Inhibitor (NuGel) for Patients with Mild to Moderate Atopic Dermatitis: Post-Hoc Biomarker Analysis"

10th Feb. 2025

Dear Editor;

I am pleased to submit our manuscript titled "A Phase 2a Placebo-Controlled Randomized Study of a Topical Inflammasome Inhibitor (NuGel) for Patients with Mild to Moderate Atopic Dermatitis: Post-Hoc Biomarker Analysis" for consideration in Frontiers in Immunology, under the Research Topic: Immunoregulation and Treatment of Allergic Diseases.

Over the past several years, our research has centered on inflammation and its regulation, building on the DAMPs model we proposed in Nature Reviews Immunology (2004; 4(6):469–78). Through our investigations into the interactions between DAMPs and pattern recognition receptors, we identified a novel pathway involving GPCR19-P2X7R-NLRP3 inflammasome. Notably, we demonstrated that a GPCR19 agonist regulates NLRP3 inflammasome activation, effectively alleviating atopic symptoms in preclinical models, including mice and dogs. This current study examines the personalized therapeutic potential of inflammasome inhibitors for managing atopic dermatitis, a condition characterized by significant clinical heterogeneity.

We evaluated the efficacy and safety of the GPCR19 agonist and conducted a biomarker-driven analysis of data from phase 2 clinical trial to identify predictors of treatment response. Our findings highlight the potential of specific baseline plasma biomarkers to predict clinical outcomes.

This manuscript is original, has not been published or submitted elsewhere, and has been approved by all authors. Furthermore, any competing interests have been fully disclosed. This manuscript meets Consolidation Standards of Reporting Trials (CONSORT) and includes required flowcharts and attached a checklist. We believe this study provides meaningful insights into the immunological mechanisms underlying atopic dermatitis and advances the field of personalized medicine in allergic diseases.

We look forward to your feedback and would be happy to provide additional information or clarification if needed.

Sincerely,

Seung-Yong Seong, M.D., Ph.D., Professor
Seoul National university College of Medicine
seongsy@snu.ac.kr

Jung-Im Na, M.D., Ph.D., Professor
Seoul National university College of Medicine
jina1@snu.ac.kr

[Response letter to Resubmission request #1]

20th Jan. 2025

Q1. Source data (raw data, original data, individual data points) for information presented in your tables and figures. This should be in a generally readable format and Excel files are preferred.

A1. We attached them as a “PeerReviewOnly.zip” file.

Q2. If statistical programs (such as R, SAS, SPSS, MATLAB) were used for the data analysis or figure creation in your manuscript, please provide all the relevant code/script files (such as .R or .SPS) and data sheets to replicate your analyses or figures.

A2. GraphPad prism files are included in a “PeerReviewOnly.zip” file.

Q3. Full scans of any entire, original gels displayed in your manuscript, the original files for any microscopy images or flow cytometry data (.fcs) included in your figures, and gating strategies for any flow experiments with clear labels to differentiate separate experiments.

A3. We don’t have gel images.

Q4. We also note that the manuscript was previously rejected at a Frontiers journal, but the resubmission provided does not address all concerns by the previous editor.

A4. I was confused. This manuscript has not been rejected from Frontiers in Immunology.

[Declaration as a topic editor of Frontiers in Immunology]

- **Exploring the Biology of Inflammasomes: Mechanisms, Disorders, and Therapeutic Potential**

I got acceptance letter as a topic editor after submitting this manuscript as follows;

Dear Dr. Seong and Dr. Magupalli,

I'm pleased to inform you that the Chief Editor of our Inflammation section, Prof. Pietro Ghezzi, has accepted your Research Topic "" for launch in *Frontiers in Immunology*.

Thank you for all the hard work you've put into this project so far.

Your Research Topic is now live on our journal website at the following link: https://www.frontiersin.org/research-topics/69318/exploring-the-biology-of-inflammasomes-mechanisms-disorders-and-therapeutic-potential

You can access the Research Topic Management platform for your Research Topic here (please ensure you are logged into your Loop account on the browser in order to access): https://research-topic-management-app.frontiersin.org/manage/69318/contributor

As a next step in the process now that the Research Topic has launched, it would be good if we could schedule a quick call so I can help you set up your Research Topic for success? During this call, I will explain:

1. What to expect following your topic's launch

2. How to use our Research Topic platform

3. How to send an impactful call for papers and how we can promote your collection together

This is also a great opportunity to meet the rest of your team if you haven't done so already.

Please vote for all the times that might work for your schedule. I will then schedule a meeting in the time slot that you can both attend.

To set your Research Topic up for success, please draft your Call for Participation invitation. This is a personalized invitation that is sent by you through our Research Topic Management platform asking your Potential Authors to contribute an article to your collection. I have attached a template for you to review and complete. We can then send this to your Potential Authors together. Another option could be to reach out to your direct network individually, and we can send the standard call for participation to the rest of your potential contributors. Could you please let me know your thoughts on the attached template and send me any additional drafts by the date of our call?

I've attached our Step-by-Step Guide to give you a head-start, and I look forward to speaking with you soon.

Kind Regards,

Aidan

Aidan Casey, PhD

Frontiers in Immunology

Frontiers | Editorial Office – Publishing Development

Senior Journal Manager: Ross McGlennon

Senior Journal Specialist: Aidan Casey

www.frontiersin.org

12 Moorgate

London EC2R 6DA – United Kingdom

T +41 (0) 21 510 17 44

[Response letter to Resubmission request #2]

10th Feb. 2025

Q. Please ensure your manuscript meets Consolidation Standards of Reporting Trials (CONSORT) reporting standards and includes any required flowcharts and/or checklists in the figures and/or supplementary material.

A. We attached a checklist file “Consort-2010-Checklist250210.doc”.

[Our publications related to inflammasome inhibitors]

Safety, Tolerability and Pharmacokinetics of Intravenous Sodium Taurodeoxycholate, HY209, a GPCR19 Agonist Inhibiting Inflammasomal Activation. *Drug Des Devel Ther. 2024 Dec 8;18:5853-5861.*

Oral Administration of Taurodeoxycholate, A GPCR19 Agonist, Effectively Ameliorates Atopic Dermatitis in A Mouse Model. *Exp Dermatol. 2024 Oct;33(10):1-7.*

Taurodeoxycholate ameliorates DSS-induced colitis in mice.
Int Immunopharmacol. 2023 Sep;122:110628.

Taurodeoxycholate, a GPCR19 agonist, ameliorates atopic dermatitis in Balb/c mice.
Eur J Immunol. 2023 May;53(5):e2250048.

GPCR19 Regulates P2X7R-Mediated NLRP3 Inflammasomal Activation of Microglia by Amyloid β in a Mouse Model of Alzheimer's Disease.
Front Immunol. 2022 Apr 6;13:766919.

Editorial: DAMPs Across the Tree of Life.
Front Immunol. 2022 Feb 1;12:844315.

Role of inflammasomes in neuroinflammation after ischemic stroke.
Encephalitis. 2021 Oct;1(4):89-97.

Evaluation of acute and subacute toxicity of sodium taurodeoxycholate in rats.
Drug Chem Toxicol. 2021 May;44(3):268-276.

Nonclinical toxicology studies with sodium taurodeoxycholate: acute and subacute toxicity in dogs.
Drug Chem Toxicol. 2021 Mar;44(2):161-169.

Taurodeoxycholate Increases the Number of Myeloid-Derived Suppressor Cells That Ameliorate Sepsis in Mice.
Front Immunol. 2018 Sep 18;9:1984.
